# Use of a modified GreenScreen tool to conduct a screening-level comparative hazard assessment of conventional silver and two forms of nanosilver

**DOI:** 10.1186/s12940-016-0188-y

**Published:** 2016-11-08

**Authors:** Jennifer Sass, Lauren Heine, Nina Hwang

**Affiliations:** 1 Natural Resources Defense Council and George Washington University Milken Institute School of Public Health, 1152 15th St NW, Suite 300, Washington DC, 20005 USA; 2 Northwest Green Chemistry and Lauren Heine Group LLC, Spokane, WA USA; 3 Green Seal, Washington, DC USA

**Keywords:** Nanomaterials, Alternative assessment, Hazard assessment, Toxic chemicals, Antimicrobials, Nanosilver, Silver, Nanotechnology

## Abstract

**Background:**

Increased concern for potential health and environmental impacts of chemicals, including nanomaterials, in consumer products is driving demand for greater transparency regarding potential risks. Chemical hazard assessment is a powerful tool to inform product design, development and procurement and has been integrated into alternative assessment frameworks. The extent to which assessment methods originally designed for conventionally-sized materials can be used for nanomaterials, which have size-dependent physical and chemical properties, have not been well established. We contracted with a certified GreenScreen profiler to conduct three GreenScreen hazard assessments, for conventional silver and two forms of nanosilver. The contractor summarized publicly available literature, and used defined GreenScreen hazard criteria and expert judgment to assign and report hazard classification levels, along with indications of confidence in those assignments. Where data were not available, a data gap (DG) was assigned. Using the individual endpoint scores, an aggregated benchmark score (BM) was applied.

**Results:**

Conventional silver and low-soluble nanosilver were assigned the highest possible hazard score and a silica-silver nanocomposite called AGS-20 could not be scored due to data gaps. AGS-20 is approved for use as antimicrobials by the US Environmental Protection Agency.

**Conclusions:**

An existing method for chemical hazard assessment and communication can be used – with minor adaptations– to compare hazards across conventional and nano forms of a substance. The differences in data gaps and in hazard profiles support the argument that each silver form should be considered unique and subjected to hazard assessment to inform regulatory decisions and decisions about product design and development. A critical limitation of hazard assessments for nanomaterials is the lack of nano-specific hazard data – where data are available, we demonstrate that existing hazard assessment systems can work. The work is relevant for risk assessors and regulators. We recommend that regulatory agencies and others require more robust data sets on each novel nanomaterial before granting market approval.

**Electronic supplementary material:**

The online version of this article (doi:10.1186/s12940-016-0188-y) contains supplementary material, which is available to authorized users.

## Background

Nanomaterials are generally defined as engineered structures having at least one dimension in the nanoscale size range of 1–100 nm (nm) [1]. For reference, the width of a piece of paper or human hair is about 100,000 nm. Nanotechnology is often touted as one of the next industrial revolutions and an increasing number of products are being created and brought to market with a wide range of applications. For example, in consumer products: nanosilver in bedsheets and sports clothing to make them resistant to bad odors from bacteria and fungi; silica nanoparticles in rubber tires for reducing rolling resistance and in personal care products such as toothpaste for abrasiveness; carbon nanotubes add strength and decrease weight in golf clubs, kayaks, and archery arrows; nanoclays increase the bounce of tennis and golf balls; and, carbon nanotubes decrease resistance and increase grip on road racing tires [2, 3]. Nanosilver is the most common nanomaterial in consumer products, including toothpaste, faces creams, cosmetics, medical bandages, disinfectants, kids' plush toys, baby blankets, towels, socks, kitchen utensils and insecticides [2, 4]. There is awareness and concern among consumers, retailers, manufacturers, researchers and regulators about the sustainability of nanomaterials used in consumer products including potential ecological and human health risks [5, 6].

Despite the prevalence of nanomaterials in consumer products, the nanomaterials going into the products that we wear, play with, store our food in, and apply as cosmetics and skin creams have undergone only limited testing for potential adverse human health or ecosystem effects. In most cases no tests have been conducted for chronic health endpoints such as cancer or neurodevelopmental effects [7]. Consumers want to make informed purchasing decisions and many are seeking greater levels of transparency about the potential health and environmental impacts of chemicals added to products. In order to do so, information is needed about what is in these products, whether it is hazardous, and how it compares to ingredients in other similar products. In response, retailers and manufacturers are increasingly turning to their supply chain for this information [8].

Chemical hazard assessment is a powerful tool to support informed decision making for product design, development and procurement [9, 10]. It is being incorporated into corporate policies, internal product design and development protocols and ecolabels and standards. The systematic gathering, classification and visual communication of hazard information helps to communicate quickly what is known and not known about the hazards associated with a particular substance and its potential transformation products.

Here, we demonstrate the use of an existing transparent and systematic method for chemical hazard assessment – GreenScreen® for Safer Chemicals (GreenScreen) - to assess and communicate hazard information and identify critical data gaps to help manufacturers and their supply chain make informed decisions when selecting materials that are non-hazardous or less hazardous than ones being replaced. This report is the first known application of GreenScreen for assessing the inherent hazards of nanomaterials.

This project provides support for the use of an existing method for chemical hazard assessment and communication – with minor adaptations described in this paper – to compare hazards across conventional silver and two kinds of nanosilver. Nanosilver forms used in textiles were considered to be well-suited to these first nanomaterial GreenScreen assessments because of the relatively robust hazard data set available in the public literature, and their widespread use in consumer clothing and other textiles that can lead to direct human exposures and environmental releases. We did not generate any new data, but relied on publicly available existing data.

While conducting chemical hazard assessments can be a complicated process for all chemicals, it can be particularly difficult for nanomaterials. Nanomaterials have the same chemical composition as their conventionally-sized counterparts, but can exhibit unique and commercially desirable properties, as well as unique and sometimes unwelcome hazards, at the nanoscale [11–13]. As the size of a particle decreases, there is a corresponding increase in the surface area per mass [12]. A material that is chemically or electrically inert at conventional scale may be more reactive at nanoscale on a per mass basis, due to the increased surface area available for chemical interactions [12]. All these size-dependent characteristics may influence the risk associated with the material [13]. In addition to enhanced reactivity, certain nanomaterials may be able to more easily penetrate bodily tissues including organs such as the brain and fetal circulation [11, 14]. Beyond differences in potential human health hazards, these size-dependent properties of nanomaterials may also affect aquatic and terrestrial ecosystems differently than conventional chemicals.

Surface coatings and other modifications can further affect the potential hazard of a nanomaterial in different media, such as soil or water [11]. So while the hazard endpoints associated with the conventional bulk chemical form remain relevant (i.e. carcinogenicity, developmental toxicity, acute toxicity), nanomaterials present challenges to existing toxicity prediction methods that are solely based on chemical composition and mass. This has stimulated governments to tackle the problem, notably the Organisation for Economic Co-operation and Development (OECD)’s Working Party on Manufactured Nanomaterials, which hosts an international body of experts focused on the human health and environmental safety implications of engineered nanoscale chemicals, including toxicity testing methods and risk assessment approaches [15].

Both conventional silver and nanosilver are approved by the U.S. Environmental Protection Agency (US EPA) Office of Pesticide Products (OPP) for use as antibacterial agents. They can both effectively kill harmful or odor-causing bacteria and appear in products including “antibacterial sheets and pillowcases” that kill bacteria and fungi, “antibacterial tableware and kitchen tools” that prevent diseases including dysentery and hepatitis, and a baby plush toy “with the additive of silver nanoparticles … to fight against harmful bacteria, molds and mites”[16–18]. Their non-specific antimicrobial properties inhibit the growth of microbes, thought to be due to a steady release of toxic silver ions (Ag^+^) or nanosilver from the surface of the molecule [14, 19]. Some low-soluble forms of nanoscale silver can be incorporated into textiles, plastics, and other materials as a polymer coating or directly imbedded into synthetic polymer fibers.

Colloidal silver is a suspension of intentionally produced nanosilver particles ranging in size from 10 to 1000 nm. It has been used for about a century by people claiming it has health benefits, despite a 1999 the U.S. Food and Drug Administration determination that colloidal silver products were neither safe nor effective [20]. Colloidal silver products marketed with medical or health benefits are now considered “misbranded” under the law, although they are still sold as homeopathic remedies and dietary supplements [20].

These antimicrobial properties also make nanosilver highly toxic to beneficial organisms, including fish and other aquatic life. Nanosilver indiscriminately kills beneficial fungi, algae, and many aquatic organisms along with the undesirable bacteria and fungi. The OECD assessed relevant studies of nanosilver, and based on the Globally Harmonized System (GHS), assigned a Category 1 toxicity score for daphnia and algae, and a Category 2 for fish (see nanosilver GreenScreen in Additional file 2). This was based on an average 96 h median lethal concentration (LC50) for nanosilver in fish (Oryzias latipes) of 1.8 mg/L, in Daphnia a median effective concentrations (EC50s) of 0.012 mg/L and in algae (Raphidocelis subcapitata) a 72 h EC50 (concentration at which a 50 % inhibition of the growth rate is observed) of 0.74 mg/L (Additional file 2, GreenScreen for nanosilver). The very high aquatic toxicity of nanosilver has raised concern about nanosilver entering the wastewater stream during washing and laundering of treated textiles, with some studies showing that as much as half of imbedded nanosilver can be lost from treated textiles during a single wash cycle [21, 22]. Toxicity to indicator species such as Daphnia suggests the possibility of adverse impacts to related aquatic organisms that comprise a healthy aquatic ecosystem, and to beneficial bacteria required to treat sewage sludge in wastewater treatment facilities. Widespread use of products containing nanosilver in homes could also lead to the proliferation of resistant microbes [23].

Human health concerns regarding ingestion, dermal contact, and especially inhalation of nanosilver particles are based on results of laboratory studies in cells and whole animals (reviewed in [24]). Whole animal studies of rats exposed to nanosilver via inhalation for 9 days reported compromised lung function and lung inflammation, as well as cellular changes in the kidney and liver [24–30]. The inhaled nanosilver released silver ions that entered the bloodstream and was then distributed to all major organs and tissues including the kidney, liver, and brain [24, 27, 31, 32]. Once in organs and tissues, in vitro cellular studies report that nanosilver causes DNA damage, genotoxicity and oxidative stress leading to apoptotic cell death [24, 33, 34]. Scientists have also identified adverse impacts to the beneficial human skin microbiome as a potential concern [35].

Because of the health concerns associated with uses of nanosilver with direct human contact, the U.S. National Institutes of Health has initiated a clinical trial to examine the potential impacts of nanosilver inhalation on human lung function [36]. The study is designed to address cleaning products and clothing that contain nanosilver, as well as solutions marketed specifically to be inhaled as purported immune system boosters. Laboratory analyses will include measuring penetration of silver nanoparticles into the blood stream, circulation through the body, and potential changes to the lung microbiome.

A draft National Institute for Occupational Safety and Health (NIOSH) Current Intelligence Bulletin on the Health Effects of Occupational Exposure to Silver Nanomaterials, which is a comprehensive review of all available published studies, concludes that there are risks of lung and liver effects including lung inflammation associated with exposure silver nanoparticles in the range of 15–20 nm [24]. NIOSH used results from rodent subchronic inhalation studies to model an estimated range of exposures from 0.19 to 195 micrograms per cubic meter (μg/m^3^) over a 45-year working lifetime. NIOSH estimated that these exposure are low enough that they would not be expected to result in adverse lung or liver effects [24, 28, 29]. The current workplace NIOSH recommended exposure limit (REL) and the enforceable Occupational Safety and Health Administration (OSHA) permissible exposure limit (PEL) for silver metal dust and soluble silver are both 10 μg/m^3^ as an 8-h time-weighted average airborne concentration [24]. While this value is expected to be protective, NIOSH has also noted that the currently available data specific to nanoscale silver are too limited to develop an REL with confidence, and thus effective workplace controls to avoid exposures and minimize risks should be put into place, along with medical monitoring of workers.

US EPA has approved two nanosilver pesticide active ingredients – both as antimicrobials for use on textiles – one called AGS-20 that is included the GreenScreen evaluation and subsequently a second called Nanosilva. There are other products on the market with nanosilver, but they have not gone through the legally-required registration and approval process. AGS-20 is a silica-silver nanocomposite. HeiQ, the registrant for AGS-20, submitted toxicity information for the oral, dermal, and inhalation route of exposure as well as eye irritation data to US EPA as part of the registration process. Due to challenges such as material characterization, data gaps, and relevancy of current toxicity testing methods for nanomaterials, US EPA sought advice from its independent Scientific Advisory Panel [14]. The Panel advised US EPA against extrapolation from silver or other forms of nanosilver, such as using read-across information from ionic and metallic silver to inform its nanosilver assessment. Disregarding the Panel’s recommendations, US EPA approved AGS-20 and subsequently Nanosilva by filling data gaps with hazard data from other nanosilver materials [37, 38]. The US EPA risk assessment relied on a 90-day rat inhalation study of uncoated nanosilver particles with an average diameter of 18 nm and immunotoxicity effects reported in a 28-day mouse oral study with uncoated nanosilver particles with an average diameter of 42 nm [28, 39]. No studies longer than 90 days (i.e. no chronic toxicity studies) were available; US EPA identified data gaps or data deficiencies for neurotoxicity, developmental and reproductive toxicity, and mutagenicity endpoints. The US EPA risk assessment applied a 10-fold uncertainty factor to adjust for these data gaps and deficiencies, resulting in a conclusion of ‘no risk’ for consumers and workers [35]. US EPA granted the nanosilver products a conditional registration approval, permitting market access immediately on the condition that registrants submit additional data over several years. The information that US EPA requested included a study of leaching from textiles, a 90-day inhalation study, a dermal toxicity study, a reproductive/developmental toxicity test, and an in vitro micronucleus assay [35].

## Methods

GreenScreen, developed by the Clean Production Action, allows users to screen and compare chemicals based on inherent hazards. GreenScreen integrates aspects of US EPA’s Design for Environment (DfE) Alternatives Assessment Criteria for Hazard Evaluation and the Globally Harmonized System (GHS) of Classification and Labelling of Chemicals to help users identify hazards associated with chemicals, to optimize product development and to identify suitable replacements [40, 41]. Assessments can be used to guide product design and materials procurement, as well as to comply with certain regulatory assessments. GreenScreen is also increasingly being incorporated into environmental scorecards and standards. For example, the assessments are now acceptable for earning LEED v4 credits and Cradle-to-Cradle® certification [42, 43].

At the most detailed level, a GreenScreen report provides a summary of the publicly available literature, test data, and modeling results on the chemical of interest and chemical analogs used to assess and classify it for a suite of 18 hazard endpoints. As part of the GreenScreen method, potential environmental transformation products are also considered across the lifecycle, including product manufacture, consumer use, and end-of-life. Based on the data, GreenScreen hazard criteria and expert judgment are used to assign and report hazard classification levels, along with indications of confidence in those assignments. All the endpoint scores are summarized in a hazard summary table to allow for ease of visualization. Where data are not available, a data gap (DG) is assigned. Using the individual endpoint scores, an aggregated benchmark score (BM) is applied ranging from Benchmark 1 (BM 1) to Benchmark 4 (BM 4). A BM 1 chemical has attributes of a substance of very high concern as defined by U.S., Canadian and European regulatory bodies. A BM 4 chemical has a complete data set suggesting low hazards to humans and the environment. BM U (unspecified) is assigned where data are insufficient to assign a benchmark score based on GreenScreen guidance.

### Modifications to GreenScreen to accommodate nanomaterials

The authors, in collaboration with expert advisors and consulting toxicologists, determined what modifications should be made to the method to accommodate nanomaterials. There was consensus that for all human and ecotoxicity endpoints, the hazard classifications should be based on data generated for the nanomaterials and not extrapolated from conventional forms of the same chemical, consistent with recommendations from the US EPA Scientific Advisory Panel (2010) that reviewed nanosilver and noted that its toxicity profile may be different from conventional silver or other forms of nanosilver [14]. The standardized GreenScreen methodology was followed. However, a number of physical property parameters were added to the reporting template to help better characterize the nanomaterials [44]. These include:

1. agglomeration and/or aggregation
2. chemical composition
3. purity
4. shape – spherical unless stated otherwise
5. surface area
6. surface charge
7. surface chemistry (including composition and reactivity). Any of the following stabilizers or capping agents are in scope: PVP (polyvinylpyrrolidone), CMC (carboxymethylcellulose), citrate, carbonate, and starch. The following strong ligands/stabilizing agents are out of scope: cysteine, anything “mercapto-”, or “thiol”, serum albumin, “passivated” nanoparticles (nanoparticles surface-derivatized and surface-functionalized with strong ligands);surfactants (without controls for inherent toxicity); nanocomposites
8. whether any characterization was conducted in the relevant experimental media.

### AGS-20 nanosilver-silica composite – material definition

AGS-20 is a nanosilver-silica composite made by a Swiss-based company called HeiQ. AGS-20 is defined as a nanocomposite consisting of particulate metallic silver (1–10 nm) and silicon dioxide having external aggregate dimensions of approximately 1 μm (μm). The US EPA approved AGS-20 for use as an antimicrobial fabric treatment on adult clothing sleeping bags, adult sportswear and other textile products. The US EPA docket for this product contains publicly available US EPA summaries of the toxicology and other studies HeiQ submitted to support the registration [45]. For this GreenScreen assessment, only studies specifically on AGS-20 were included.

### Low-soluble nanosilver– material definition and exclusion criteria

Since GreenScreen was developed for individual chemicals, a fundamental challenge encountered at the outset was conducting an assessment for nanosilver, which encompasses a class of diverse nanomaterials. Nanosilver materials can be synthesized using an array of different reducing agents, and are stabilized with diverse types of capping agents and dispersants; differences that may influence the outcome of hazard and fate studies. For these reasons, the silver forms within the scope of the assessment were carefully defined, and studies were restricted to those that adequately characterized the test substance.

The “nanosilver” GreenScreen was conducted on nanoscale silver chloride (CAS # 7783-90-6) and nanoscale silver (CAS # 7440-22-4), dispersed spherical metallic silver with external dimensions in the range of approximately 1 to 100 nm. To account for differences in synthesis, only studies that clearly identified the chemical composition, including stabilizing agents and particle size distributions were included in the assessment. Nanoparticles prepared from silver chloride were included in the assessment, because they are often used in textiles and are anticipated to have similar properties to elemental silver. Other forms of nanoscale silver used for textile applications were considered too chemically dissimilar from this study’s definition of nanosilver to be used. Nanosilver stabilized by nonionic polymers (e.g., poly(vinyl pyrrolidone) (PVP)) or surfactants (e.g., Polysorbate 80) were included, while those stabilized by inherently toxic ionic surfactants were generally excluded. Also excluded were nanosilver particles prepared with strong ligands or stabilizing agents to reduce the release of silver ions, as they are unlikely to confer the commercially desired antimicrobial properties.

In addition to metallic nanosilver, nanosilver chloride, and AGS-20, other forms of silver used for textile applications include nanoscale silver zeolite (crystalline, hydrated alkali-aluminum silicates) and silver nitrate, which may be used in the synthetic preparation of silver nanomaterials. These other forms are sufficiently chemically dissimilar from the selected forms and were specifically excluded from the scope of this study. Therefore, data for ionic silver, highly soluble silver compounds (e.g. silver nitrate), and moderately soluble organic silver salts (e.g. silver acetate) were excluded.

### Conventional silver– material definition and exclusion criteria

The GreenScreen conducted on “conventional silver” followed standard methods for GreenScreen without adaptions. Conventional silver was defined as inorganic low-solubility dispersed metallic silver and silver salts having dimensions in the range of 300 nm to 5 μm. Only studies on poorly soluble forms of silver were assessed in order to reflect the low-solubility of the two nanoscale silvers.

### Data search strategy

In accordance to the GreenScreen method, a literature search was conducted to identify publicly available data for the three silver compounds. Only studies published prior to the latest update (October 2015) are included in this report. Typically, studies are identified using the chemical name and Chemical Abstract Service (CAS) Registry Number. However, due to the unique size restrictions related to nanoscale materials, CAS numbers were either unavailable or insufficient to conduct an inclusive search and adjustments were necessary to search for nanosilver and AGS-20. For nanosilver, a modifying search term was used in combination with the CAS Registry Number on the Toxline database. Results were supplemented with data from the International Council on Nanotechnology (ICON) Environmental Health and Safety (EHS) Virtual Journal and US EPA’s State of the Science Literature Review on Nanosilver. The only AGS-20 specific data was located in the US EPA Docket for AGS-20 to support its registration as an antimicrobial pesticide [46].

Relevant publications from the literature search were reviewed. To evaluate the adequacy, quality, and relevance in terms of textile applications, the eight characterization parameters stated above and standard Klimisch criteria of study design and quality were considered. Publications that failed to identify the chemical composition (including capping or stabilizing agents), particle size distribution, and shape were excluded due to the influence those characteristics likely have on hazard.

### Screening level hazard assessment

The GreenScreen assessments encompassed the following 18 hazard endpoints: cancer, mutagenicity, reproductive toxicity, developmental toxicity, endocrine activity, acute mammalian toxicity, systemic toxicity (single and repeated exposure), neurotoxicity (single and repeated exposure), skin sensitization, respiratory sensitization, skin irritation, eye irritation, acute aquatic toxicity, chronic aquatic toxicity, environmental persistence, bioaccumulation, reactivity, and flammability. Exposure routes considered were limited to oral, dermal and inhalation. A hazard classification score for each endpoint was determined using GreenScreen criteria with possible scores, including very low, low, moderate, high, very high, or data gap [47]. Summaries of the data used to assess each material are available in the respective GreenScreen reports in the Additional files 1, 2 and 3.

### Applying the GreenScreen benchmarking process

The GreenScreen version 1.2 Benchmark (BM) system was used to provide an overall hazard score for silver and the nanoscale silver substances. One of four possible BM scores was assigned by applying a pre-defined set of algorithms to the hazard endpoint classifications. The BM levels are: BM 1 - Avoid - chemical of high concern, corresponds with high or very high concerns for key human and/or environmental endpoints; BM 2 - Use - but search for safer substitutes, corresponds with moderate to high environmental and human health concerns; BM 3 - Use - but still opportunity for improvement, has some environmental and/or human health concerns; BM 4 - Prefer - safer chemical, has low hazards across all 18 endpoints and no data gaps.

There are several triggers for a BM 1 score that do not require a comprehensive data set, however BM 2 and higher designations have increasingly stringent requirements for data completeness. If the substance is not designated BM 1 and has not been sufficiently tested to achieve the minimum data requirements for BM 2, 3, or 4, it may be assigned a “BM U” for Unspecified hazard. Detailed information about how BM scores are assigned is in the GreenScreen Procedure Guide [47].

## Results

Conventional silver was assigned a BM 1 based on very high persistence and very high aquatic toxicity as determined by standardized tests. Significant data gaps for human health hazards were present, including carcinogenicity, reproductive and developmental toxicity, endocrine disruption, both acute and chronic systemic toxicity and neurotoxicity, and respiratory sensitization.

Nanosilver was assigned a BM 1 based on very high persistence, high repeat dose systemic toxicity and very high ecotoxicity. The high repeated dose systemic toxicity is based on animal studies where exposure occurred via inhalation. Nanosilver received a moderate (low confidence) hazard score for repeated dose neurotoxicity, based on animal studies with non-standard routes of exposure (intragastric, intranasal, and subcutaneous). Data gaps existed for carcinogenicity, endocrine disruption, single dose systemic toxicity and neurotoxicity, and respiratory sensitization.

**Fig. 1:**
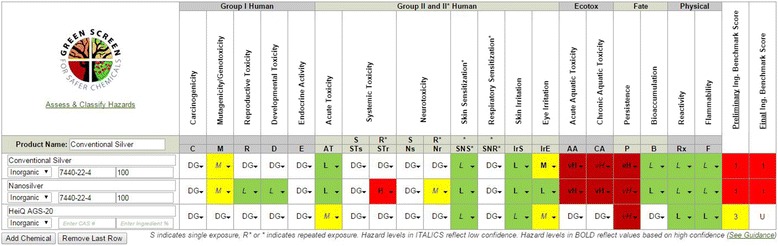
Summary-level hazard tables for Conventional Silver, Nanosilver, and AGS-20. S indicates single exposure, R* or * indicates repeated exposure. Hazard levels in italics reflect low confidence and hazard levels in bold reflect values based on high confidence

AGS-20 lacked data that would have triggered a BM 1, such as carcinogenicity, and had too many data gaps to be assigned a BM 2, 3, or 4. It received a score of moderate hazard (low confidence) for acute toxicity via inhalation and eye irritation – adverse effects that are possibly due to the silica portion of its composition. As a result of insufficient data, AGS-20 received a BM U (Fig. 1).

## Discussion

For this project, we decided *a priori* that all of the hazard assessments, including that for AGS-20, would be based only on data for the specific nanomaterial. By following the US EPA Scientific Advisory Panel’s recommendation against extrapolating between nanomaterials, given how little is understood, we identified many data gaps, including for all the chronic human health endpoints [14]. US EPA approved the material for use in consumer textiles despite lacking any subchronic or chronic effects data specific for AGS-20. Instead, US EPA OPP used several silver-based nanomaterials as analogs for fulfilling AGS-20 data requirements for subchronic toxicity, reproductive and developmental toxicity, or genetic toxicity. Those analogs were pure nanosilver particles (18–19 nm), pure nanosilver particles (60 nm), nanosilver (size not disclosed), and nanosilver coated wound dressing (no further info on size or auxiliary ingredients was reported) [28, 31, 33, 48–50]. It is unclear whether US EPA’s approach of using analog data from different nanosilver materials is an appropriate hazard predictor or not, although it ran counter to the recommendations of its Scientific Advisory Panel (2010) [14]. In addition, transformation products of AGS-20 are unknown – adding to the uncertainty associated with the use of this material [51].

GreenScreen builds on GHS Classification and Labelling and the US EPA Design for the Environment Alternatives Assessment Criteria for Hazard Evaluation, which were both developed primarily for conventionally-sized chemicals [40, 52]. While this limitation must be acknowledged, we believe it can be addressed for many nanomaterials with a thoughtful and flexible application of the tool, and careful documentation of modifications, etc.

Data gaps are typically a challenge for generating comprehensive hazard profiles for all chemicals. However, data gaps are likely to be more extensive for nanomaterials and other emerging chemicals. Aside from the specified modifications to the method, GreenScreen guidance was applied as written for this project, resulting in classifying hazards when data were available and identifying data gaps when data were not available. Even different forms of the same conventionally-sized substance can result in different hazards. For example, consider of the example of silica dust reviewed by the International Agency for Research on Cancer (IARC); crystalline silica (in the form of quartz or cristobalite) causes lung cancer (IARC Group 1, known to cause cancer in humans), but studies of amorphous silica have reported that it is not linked to cancer (IARC Group 3, unable to be classified) [53]. A recent Opinion of the Scientific Committee on Consumer Safety (SCCS) that reviewed the nano form of silica in cosmetic products concluded that the available data was “inadequate and insufficient” to draw any firm conclusions regarding safety or risks [54]. Hazard assessment of nanomaterials is an extension of these size form-specific hazard assessment challenges, and something GreenScreen can address. Future development of GreenScreen could explore the use of high throughput data and non-standardized test methods for filling data gaps.

One limitation of the method has to do with the use of data from Safer Data Sheets (SDSs) to classify hazards associated with flammability and reactivity. The standard GreenScreen method, initially developed for conventional organic chemicals, allows for the use of data from SDSs to classify hazards associated with flammability and reactivity, but are not allowed for the classification of any of the other hazard endpoints. We believe that secondary data are not sufficiently specific to nanomaterials and may lead to inaccurate hazard classifications. For example, combustible dusts include most solid organic materials (such as sugar, flour, grain, wood, etc.), many metals, and some nonmetallic inorganic materials. Some of these materials are not normally combustible, but can burn or explode if the particles are small enough and accumulated at a high enough concentration. This raises a reasonable concern that nanosilver may act more like a dust or powder than a liquid and therefore present a risk for flammability and/or explosion. This may also be the case for carbon nanomaterials [55]. An SDS for silver powder states that, “this material, like most materials in powder form, is capable of creating a dust explosion” [56]. Whether or how to address the potential became a difference of opinion between the clients (the authors of this paper) and the contractors hired to conduct the GreenScreen assessments. The nanosilver GreenScreen report produced by the contractors notes that “silver powder (particle size was 90 % > 0.5 μm) was determined to be nonflammable according to the EU Method A.10 (Flammability (Solids)” and that “mechanical impact on powders may result in explosion, although bulk silver is not explosive.”

We recommend that GreenScreen practitioners not use information for conventionally sized materials when evaluating nanomaterials for any GreenScreen hazard endpoints, including flammability and reactivity, unless there are adequate data to justify it.

GreenScreen has a built-in preference for regulatory studies, often sponsored by industry, over hypothesis-driven studies more often conducted in academic settings. This is because GreenScreen prefers data conducted according to internationally harmonized test methods (OECD Test Guidelines or equivalent) and performed in laboratories certified for Good Laboratory Practices (GLP) – both criteria required for studies conducted to support chemical registrations, but not required for academic research.^1^ Internationally harmonized test methods (OECD Test Guidelines or equivalent) are designed from a regulatory standpoint to promote consistency. Research for regulatory purposes typically follows standardized guidelines, due to the regulatory requirements, in laboratories that are GLP certified. Academic research, on the other hand, is typically curiosity-driven and thus diverges from standardized methods [57]. However, consistent data should not be conflated with the assumption that the best available tools are used and that the appropriate questions are being asked; regulatory study guidelines may not reflect the current science, most sensitive disease endpoints, chronic disease outcomes, or novel materials such as nanomaterials. In contrast, OSHA has issued Guidance on Data Evaluation for Weight of Evidence Determination stating that a hazard classification can be based on a single test showing adverse effects if it is conducted according to “good scientific principles” without ever mentioning GLP. We suggest that GreenScreen consider updating its guidance similarly.

GreenScreen has a preference for studies which receive a high Klimisch score - a tool used widely to score the reliability of toxicological studies [58], which has recognized value for regulatory research, but may not be as appropriate for hypothesis-driven academic research [59].

GreenScreen has no requirements for reporting on study sponsorship. We suggest that GreenScreen require reporting of author sponsorship if known, when reporting study results to provide a measure of transparency regarding potential conflicts between study sponsorship and study results [59–63].

The preference for regulatory studies could be amplified by GreenScreen’s deferral to hazard listings of regulatory bodies, such as US EPA, which can be heavily influenced by the regulated community, [64–67]. For example, US EPA used only industry-supplied data to assess all the hazard endpoints for AGS-20 in its registration and approval process. GreenScreen procedures specify that a profiler can only override an authoritative body if there are new data not included in the authoritative body’s assessment. This can be a very significant limitation that appears to prevent challenging regulatory assessments. We recommend that GreenScreen be applied in a flexible way – such as what we have done for this project – as long as all modifications are accurately and transparently documented.

Given the unique physical and chemical characteristics of nanomaterials, it is likely that existing alternatives assessment frameworks and associated methods will need to be adapted. For that reason, others are also exploring methods to advance alternatives assessments for the evaluation of nanomaterials. For example, the NanoRiskCat is a stepwise tiered decision support tool developed by the Technical University of Denmark with public funding from the Danish Environmental Protection Agency and Denmark National Research Centre for the Working Environment [68]. NanoRiskCat incorporates both exposure and hazard information to provide screening-level information on nanomaterials in consumer products in an easy-to-read color system to score results. In addition to including most of the same hazard endpoints as GreenScreen, NanoRiskCat also considers how readily the material is dispersed and how novel it is. A report on the NanoRiskCat screening tool is publicly available from the Danish Environmental Protection Agency’s website [69].

## Conclusions

This project demonstrates that a comprehensive and credible comparative chemical hazard assessment can be performed on nanomaterials. The GreenScreen method was used with minor modifications to better characterize form-specific properties. Several additional modifications to the GreenScreen method are recommended for future applications. This includes a recommendation to disallow the use of MSDS/SDS data for flammability and reactivity on a nanomaterial unless there are sufficient data on the nanomaterial to justify it. In addition, a modified GreenScreen for use in assessing human and ecotoxicity data should not be heavily weighted toward the use of test data based on GLP, and instead allow for expert interpretation of scientific studies as the understanding of nanotoxicology continues to become developed and refined. Continued efforts to include new research and testing approaches to assess hazards should also be evaluated for inclusion.

A screening level hazard assessment like the GreenScreen method is not a risk assessment. Rather it provides relatively robust, rapid, and cost-effective information about what hazards concerns may exist about a chemical or product, where hazard data are absent, and how this compares to alternate chemicals or products that could be used instead. If desired, exposure considerations can be incorporated with the hazard information into a robust risk or alternatives assessment. GreenScreen can be used to inform decisions about hazards relevant to specific uses, but it cannot be used to eliminate risk - calling a material ‘safe’ or ‘non-toxic’ should require a much more rigorous threshold of evidence and consideration of how it is used. Dr. Melnick, retired career National Institute of Environmental Health Science (NIEHS) scientist, stated that “declaring a chemical as not hazardous, or reducing a level of health protection, should require validation, not speculation” [70].

Based on this work, we demonstrate that it is possible to assess and communicate hazards associated with nanomaterials as unique materials using GHS-based hazard frameworks such as GreenScreen. Because it is feasible to do so, we recommend that the U.S. adopt requirements for nanomaterials to be assessed as new chemicals to ensure greater accuracy of hazard and risk assessments. There is precedent for this recommendation. For example, the European Biocides Regulation No. 528/2012 which replaced the older Biocidal Product Directive in 2013 explicitly requires products containing nanomaterials to be assessed and labelled as such. Chemical hazard assessment methods such as GreenScreen support both quality hazard characterization and hazard communication which can in turn support transparency in governance.

## Additional files

Additional file 1: GreenScreen for Conventional Silver 2015. (DOCX 141 kb)
Additional file 2: GreenScreen for Nanosilver 2015. (DOCX 180 kb)
Additional file 3: GreenScreen for HeiQ AGS20 2015. (DOCX 93 kb)

## Abbreviations

BM: GreenScreen benchmark
BM U: GreenScreen benchmark unspecified hazard
GHS: Globally harmonized system of classification and labelling of chemicals
GLP: Good laboratory practice
GS: GreenScreen ®
MSDS: Material safety data sheets
NIEHS: National Institute of Environmental Health Science
NIOSH: National Institute for Occupational Safety and Health
nm: Nanometer
OECD: Organisation for Economic Co-operation and Development
OPP: United States Environmental Protection Agency’s Office of Pesticide Programs
OSHA: US Occupational Safety and Health Administration
REL: Recommended exposure limit
SDS: Safety data sheets
US EPA: US Environmental Protection Agency
μg/m^3^: Microgram per cubic meter
μm: Micron

## Footnotes

1 The GreenScreen Guidance states that a hazard endpoint will be classified as low confidence if it is determined using a single non-GLP or non-guideline study (GS Section 11.2.3). GreenScreen is harmonized to the Globally Harmonized System of Classification and Labelling of Chemicals (GHS), whose Guidance states that when assessing data quality, “the precise use of the available data will be described in the relevant section but, as a general rule, data generated to standard international guidelines and to GLP is to be preferred over other types of data” (GHS 4th Rev 2011; Section A9.2.6.1).
