# Supplementary material for: Use of a modified GreenScreen tool to conduct a screening-level comparative hazard assessment of conventional silver and two forms of nanosilver
Source: Environ Health. 2016 Nov 8;15:105. doi: 10.1186/s12940-016-0188-y (PMC5101654; doi:10.1186/s12940-016-0188-y)
Supplement: Additional file 1: — GreenScreen for Conventional Silver 2015. (DOCX 141 kb) [file 12940_2016_188_MOESM1_ESM.docx]

**Chemical Hazard Assessment for *low-solubility, non-nanoscale1) silver (CAS # 7440-22-4)***

**Modified from GreenScreen^®^ Version 1.2^[[1]](#footnote-1)^**

| **Initial Modified GreenScreen^®^ Assessment Prepared By:** | **Initial Modified GreenScreen^®^ Assessment Quality Control Performed By:** |
| --- | --- |
| Name: Nancy Linde; Toxicologist  Teresa McGrath, Supervising Toxicologist | Name: Caroline English, Ph.D., D.A.B.T., |
| Title: | Title: Senior Toxicologist |
| Organization: NSF International | Organization: NSF International |
| Date: January 10, 2013  Revised: December 13, 2013  Revised: February 19, 2015 | Date: January 10, 2013  Revised: December 13, 2013  Revised: February 19, 2015 |
|  | **Updated by:** Eric Rosenblum, Ph.D., D.A.B.T  Organization: Rosenblum Environmental LLC  Date: October 31, 2015 |

**Confirm application of the *Disclosure and Assessment Rules and Best Practice*^[[2]](#footnote-2)^:** (List disclosure threshold and any deviations)

because this review is based on generic silver, not a particular manufacturer’s product, the de minimus rule is not applicable.

**Chemical Name (CAS #):** Silver, CAS # 7440-22-4, restricted to the non-nanoscale particulate silver (> ~100 nm and up to respirable particle sizes).

**Suitable analogs or moieties of chemicals used in this assessment (CAS #’s):**

Silver Chloride (> ~100nm) (CAS # 7783-90-6) and Silver (II) Oxide (> ~100nm) (CAS # 1301-96-8)

**Chemical Structure(s):**

*Note: Include chemical structure(s) of all surrogates, analogs (and /or moieties) used in the assessment.

Ag (see description of properties below)

AgCl (silver chloride, non-nano)

AgO (silver oxide, non-nano)

**Justification for Chemical Surrogates:**

To the extent that the mammalian toxicity of conventional silver is a function of dissolved, soluble silver, toxicity should be similar among various silver compounds due to the ubiquitous presence of chloride ion in physiological systems. The dissolved, bioaccessable silver was determined for silver metal, disilver oxide, and silver nitrate, incubated with various artificial physiological media (ECHA, 2012). For these three compounds, with solubility characteristics spanning insoluble silver metal to soluble silver nitrate, the dissolved concentrations of silver were very similar and independent of the original silver compound (silver metal, disilver oxide, silver nitrate). The authors hypothesized that the complex ionic environment and the likely formation of poorly soluble silver chloride leads to very similar equilibrium concentrations of dissolved silver, independent of the originating substance (ECHA, 2012).

Likewise, the ecotoxicity hazard of conventional silver depends upon silver bioavailability, which is recognized to be a function of water chemistry. The Ecotoxicity Hazard Score derived in this Green Screen is considered to be theoretical and based on the results of standardized tests that do not necessarily represent the dynamic environmental conditions experienced in the field. The Biotic Ligand Model is a metal bioavailability model that was developed to incorporate metal speciation and the protective effects of competing cations into predictions of metal toxicity (WHO, 2002). Use of the Biotic Ligand Model may therefore be advisable in predicting a more pragmatic estimate of ecotoxicity hazard than what is conservatively provided in this Green Screen.

Poorly soluble forms of conventional silver were selected for this assessment for the following reasons: 1) To enable a direct comparison with poorly soluble forms of nanoscale silver in a companion assessment and 2) due to the rapid dissociation of silver nitrate in aqueous media, it can deliver soluble silver at a high rate, potentially causing excessive toxicity before equilibrium conditions prevail .

Data for ionic silver, highly soluble silver compounds (e.g. silver nitrate), and moderately soluble organic silver salts (e.g. silver acetate) was not used in this report to fulfill datagaps, primarily because soluble silver was determined to be outside of the scope of this GreenScreen Assessment. NSF acknowledges however that since more soluble forms of silver generally result in greater toxicity due to greater ion release, it would not be unreasonable to expand the scope in a future review to include data on the more soluble forms for fulfilling data gaps for metallic silver. Where applicable, this GreenScreen report does indicate where data on the soluble salts were available and not considered.

**Notes related to production specific attributes^[[3]](#footnote-3)^:**

**For Inorganic Chemicals and relevant particulate organics (*if not relevant, list NA*)**

**Define Properties:**

The following inorganic chemical characteristics were examined in each study and reported where available as part of the assessment of study quality and relevance:

1. Particle size: mean or median > ~100 nm and up to the respirable range if exposure is by inhalation.
2. Structure
3. Mobility (e.g. Water solubility, volatility).
4. Bioavailability
5. Chemical composition
6. Purity
7. Whether any characterization was conducted in the relevant experimental media.

**Identify Applications/Functional Uses:**

**(e.g., Cleaning product, TV casing)**

1. Textile applications as an antimicrobial fabric protector (e.g. silver coated fibers).

**GreenScreen Benchmark Score and Hazard Summary Table:^[[4]](#footnote-4),^^[[5]](#footnote-5),^^[[6]](#footnote-6),^^[[7]](#footnote-7)^ Conventional Silver** was assigned a Benchmark Score of **1 based on combined very high persistence coupled with very high aquatic toxicity, as determined in standardized tests.** Note, this particular combination of hazards is a trigger for Benchmark 1, and this score could not improve with fewer data gaps. It is possible, however, that application of the Biotic Ligand Model to account for metal bioavailability under environmental conditions, could improve the very High Ecotoxicity score due to the protective effects of competing cations. For more information, refer to the U.S.EPA website regarding use of the Biotic Ligand Model and WHO (2002).

As a word of caution, a data gap should not be interpreted as implying hazard or safety, but rather there was insufficient data to characterize the hazard as low, moderate, or high.

Note: Hazard levels (Very High (vH), High (H), Moderate (M), Low (L), Very Low (vL)) in *italics* reflect estimated values, authoritative B lists, screening lists, weak analogues, and lower confidence. Hazard levels in **BOLD** font are used with good quality data, authoritative A lists, or strong analogues. Group II Human Health endpoints differ from Group II* Human Health endpoints in that they have four hazard scores (i.e., vH, H, M and L) instead of three (i.e., H, M and L), and are based on single exposures instead of repeated exposures.

**Environmental Transformation Products and Ratings^[[8]](#footnote-8)^:**

**Identify feasible and relevant environmental transformation products** **(i.e., dissociation products, transformation products, valence states)** **and/or moieties of concern**^[[9]](#footnote-9)^

| **Life Cycle Stage** | **Transformation Pathway** | **Transformation Products** | **CAS #** | **On CPA Red List^^[[10]](#footnote-10)^^?** | **GreenScreen™Rating^^[[11]](#footnote-11)^^** |
| --- | --- | --- | --- | --- | --- |
| Textile Production (masterbatch, fiber and fabric preparation, fabric padding process) | Silver ion release | Silver ion (Ag+) | 14701-21-4 | No |  |
| Consumer use (release from textiles during wear and washing) |  |  |  |  |  |
| End of life (release, dissolution, aggregation, precipitation) |  |  |  |  |  |
| Textile Production (masterbatch, fiber and fabric preparation, fabric padding process) | In the presence of hypochlorite, elemental silver may oxidize and convert into silver chloride (EPA-HQ-OPP-2009-1012-0015.pdf, EPA, 2010). | Silver chloride (AgCl) | 7783-90-6 | No |  |
| Consumer use (release from textiles during wear and washing) |  |  |  |  |  |
| End of life (release, dissolution, aggregation, precipitation) |  |  |  |  |  |
| End of life (release, dissolution, aggregation, precipitation) |  |  |  |  |  |
| End of life (release, dissolution, aggregation, precipitation) | Release / dissolution / aggregation / precipitation | Silver sulfide (Ag_2_S) | 21548-73-2 | No |  |
| End of life (release, dissolution, aggregation, precipitation) | Release / dissolution / aggregation / precipitation | Silver thiosulfate (Ag_2_H_2_O_3_S_2_) | 23149-52-2 | No |  |

**Introduction:**

Silver is an EPA registered active ingredient in numerous pesticide products, including antimicrobial treated textiles. NSF recently performed a GreenScreen™ assessment to characterize the hazards of inorganic, low solubility nanosilver in textiles. In the present report, NSF performs a GreenScreen™ assessment on conventional (low-solubility, non-nano) silver for comparison.

**Hazard Classification Summary Section:**

**For all hazard endpoints:**

- **Search all GreenScreen specified lists. Report relevant results either in each hazard endpoint section or attach to the end of the report.**
- **Always indicate if suitable analogs or models were used.**
- **Attach modeling results (See Appendix C).**
- **Include all references either in each hazard endpoint section or at the end of the report.**

**Group I Human Health Effects (Group I Human)**

**Carcinogenicity (C) Score (H, M or L):** DG

Conventional silver was assigned a score of **Data Gap** for carcinogenicity based on lack of data.

- Authoritative and Screening Lists
  - *Authoritative:*
    - *US EPA - IRIS Carcinogens - (1986) Group D - Not classifiable as to human carcinogenicity*
  - *Screening: Not present on any screening lists*
- No cancer studies of silver or silver compounds were found for inhalation, oral, or dermal exposure in humans or animals (ATSDR, 1990).
- **EPA’s Cancer Classification for Silver is Group D – Not Classifiable as to Human Carcinogenicity.** The basis for the classification is inadequate evidence. EPA notes that while local sarcomas have been induced after implantation of films and disks of silver, the interpretation of these findings has been questioned due to the phenomenon of solid-state carcinogenesis in which even insoluble solids such as plastic have been shown to result in local fibrosarcomas (IRIS, 2003). It should be noted this designation is not specific to any particular chemical or physical form of silver.
- The Japanese NITE classification for carcinogenicity is “classification not possible”. This was based on descriptions that carcinogenicity was not observed in the test in which powder was intramuscularly injected to rats as reported in Patty’s Industrial Hygiene (5^th^, 2001), there was no carcinogenic evidence to humans as reported in Patty’s Industrial Hygiene (5^th^, 2001) and HSDB (2003), and there is also no information of classification evaluation from organizations such as IARC (NITE, 2006).

**Mutagenicity/Genotoxicity (M) Score (H, M or L):** *M*

Conventional silver was assigned a score of ***Moderate*** (low confidence) for mutagenicity based on limited or marginal evidence of mutagenicity in *in vitro* genotoxicity studies using bacteria and nonhuman mammalian cell cultures. As stated in the 1990 ATSDR profile for silver “existing data on mutagenicity are inconsistent, but data on genotoxicity suggest that the silver ion is genotoxic. From the results of in vitro genotoxicity studies using bacteria and nonhuman mammalian cell cultures it is evident that the silver ion does bind with DNA in solution in vitro, and that it can interact with DNA in ways that cause DNA strand breaks and affect the fidelity of DNA replication. However, silver has not been found to be mutagenic in bacteria. The low confidence is assigned as the score is based on results only observed within *in vitro* studies.

Authoritative and Screening Lists

- - *Authoritative: Not on any authoritative lists*
  - *Screening: Not on any screening lists*
- The Japanese NITE classification for germ cell mutagenicity is “classification not possible” based on lack of data (NITE, 2006).

*In vivo -* mammalian

- Groups of Crl:CD-1 (ICR) BR mice/dose were administered silver chloride (>99.5% a.i.) in a homogenous suspension of corn oil by i.p. injection at 31.25, 62.4, and 125 mg/kg, in accordance with OECD guideline 474. In a previous range finding study, 125 mg/kg was determined to be the maximum tolerated dose. Bone marrow was harvested at 24 hours from 6 animals/dose. At 48 hours, 12 additional animals were sacrificed, 6 for vehicle controls, and 6 for the 125 mg/kg dose level. Three additional animals were also dosed at 125 mg/kg to ensure survival of 6 animals for bone marrow extraction. Corn oil was the solvent control, and cyclophosphamide was the positive control. The test article induced signs of clinical toxicity as rough hair coat and/or hunched posture at 62.5 and 125 mg/kg. **The test article did not induce statistically significant increases in micronucleated polychromatic erythrocytes (PCEs) when compared to vehicle control responses.** There was a statistically significant decrease in PCE:NCE (normochromatic erythrocytes) ratio for the 125 mg/kg dose group, thus confirming that the test article reached and was cytotoxic to the bone marrow (Erexson, 2004).
- Balb/c mice were given 2.5 g of 13 nm silver nanoparticles or **2-3.5 µm silver microparticles**, directly into the stomach, and the livers were examined 3 days later for histopathological analysis. [Authors did not report the number of mice or the number of exposures, the latter is presumably one]. Nano- and micro-silver exposed mice livers **demonstrated lymphocyte infiltration, suggestive of inflammation.** From a microarray analysis of the RNA from the livers, the **expression of genes related to apoptosis and inflammation were confirmed to be altered,** and these gene expression changes may lead to phenotypical changes resulting in increased apoptosis and inflammation. **However, there was almost no reduction in mitochondrial activity** for either the nano- or micro-silver. While DNA contents were decreased up to 18% for nano-particle exposed livers, and up to 10% for micro-particle exposed livers, the nano-group had no dose-dependency, the macro-group had only weak dose-dependency, and **neither group expressed increases in glutathione production** which is normally associated with increased oxidative stress. The authors also reported *in vitro* exposure of human hepatoma cells (Huh-7) to the silver nano- and micro-particles, and noted the mitochondrial activity and glutathione production was not appreciably affected with DNA contents decreased by 15% in the nano-particle treated cells, and 10% in the micro-particle treated cells [further details were not provided] (Cha et al., 2008). [This is not a recognized assay, genotoxic significance is not established].

*In vitro* and/or non-mammalian

- A Comet assay and analysis for bioaccumulation was performed in the polychaete, *Nereis diversicolor.* Groups of 3-5 worms were exposed to nominal concentrations of nanosilver (<100 nm, 99.5% metals basis, coated by 0.2 wt% PVP), **micro-silver** (2-3.5 µm, ≥99.9% trace metal analysis), and ionic silver (AgNO_3_) at 0, 1, 5, 10, 25, and 50 µg/Ag/g dry weight (dw) sediment for 10 days. There was one control worm per group, plus one positive control worm with cells extracted and exposed to UV light, and PVP-controls to examine if the coating was responsible for the genotoxicity of the nano-sized particles. **DNA damage, measured as tail moment and tail DNA intensities, was dependent on dose and silver-form. Damage was significantly higher at 25 and 50** µg/**g dw in nano- and micro-Ag treatments**, and at 50 µg/g dw for the ionic silver, compared to controls. The presence of highly crystalline material was observed in nanosilver, suggesting the presence of large silver particles (aggregates, 20-200 nm, average 162 nm). **For micro-silver, 5-10% of non-crystalline material was observed, suggesting it was not as pure as described by the manufacturer (i.e. <99.9% purity), and had both micro- and nano-sized particles (8nm – 3 µm).** Reported silver body burdens for the nano-, micro-, and ionic-silver treatments were 8.56, 6.92, and 9.86 µg/g dw, respectively. These values correspond to BAF factors of 0.17, 0.14, and 0.20, respectively (Cong et al., 2011). [This Comet assay did not include controls for apoptosis, the relevance of the measured DNA strand breaks to heritable genetic damage is uncertain, and the relevance of this species to human health is not known].
- Lead (99.9% purity, 10 µm), Bismuth (99.9%, 10 µm), Indium (99.9%, 45 µm), Silver (99.9%, 10-20 µm) , and Antimony (99.9%, 10 µm) were tested for genotoxicity using a reverse mutation assay and a chromosome aberration test. **Test substances were suspended in DMSO. Micro- silver** in DMSO, **was negative in the reverse mutation assay in *Salmonella typhimurium* strains TA100, TA1535, TA98, and TA1537, and in *Escherichia coli* WP2uvrA/pKM101**, with and without metabolic activation at doses ranging from 313 µg/plate to 5,000 µg/plate, based on less than a two-fold increase in the mean number of revertant colonies compared to the negative controls. Microbial toxicity was not observed in any of the tester strains with or without S9 mix, although precipitates were found at silver doses of 2,500 µg/plate and higher. In the **chromosome aberration test**, the test substances were suspended in 1% sodium carboxymethylcellulose (CMC-Na), and exposed to **Chinese hamster liver cells** (CHL/IU cell line). Evaluations were performed for growth inhibition and chromosome aberrations, both structural and numerical. Micro-scale Silver was dosed at up to 5,000 µg/mL, with and without S9 mix. **Results were negative based on < 5% of either type of aberration at any dose, in the presence and absence of S9. The IC50 for growth inhibition was >5,000 µg/mL** with and without S9, suggesting the **Silver was not cytotoxic** to the CHL cells (Asakura et al., 2009). [Results for the lead, bismuth, indium and antimony are not summarized here because they are outside the scope of this paper]**.**
- Kanematsu et al. (1980) tested 127 metal compounds, including silver chloride, silver nitrate, and silver sulfate, for DNA damage in *Bacillus subtilis*. Criteria for a positive finding consisted of a more pronounced inhibition of cellular growth with the recombination-repair-deficient (*rec^-^*) than with wild bacteria (*rec^+^*), indicative of DNA damage. Samples were exposed at 0.005-0.5M of each test substance and exposed per a cold incubation method. All three silver forms were reported as **negative** is this assay (Kanematsu et al., 1980). [Specific results and statistical analyses were not reported].
- Mutagenicity data for Silver sulfadiazine (AgSu) was found, however, not considered within the scope of this GreenScreen assessment (McCoy and Rosenkranz, 1978).
- Robison et al. (1982) examined DNA strand breaks in **Chinese hamster ovary (CHO) cells**. The cells were exposed *in vitro* to crystalline (1-4 µm) nickel sulfide, cobalt sulfide, cadmium sulfide, **silver sulfide**, copper sulfide and trinickel disulfide at **10 µg/mL** for 24 hours. DNA strand breaks were determined by analysis of the number average molecular weight of DNA compared to controls. All the insoluble crystalline sulfides **induced considerable reductions in the MW of the DNA**. Authors refer to a secondary reference (Costa et al., draft paper) which postulates that these compounds phagocytosed by the CHO cells in response to the DNA breakage. Authors further suggest the strand breaks

**Reproductive Toxicity (R) Score (H, M, or L):** DG

Conventional silver was assigned a score of **Data Gap** for reproductive toxicity based on lack of mammalian data. While one study reports no decrease in fertility in male rats it was poorly reported and therefore cannot be used to determine a hazard score with adequate confidence.

- Authoritative and Screening Lists
  - *Authoritative: Not on any authoritative lists*
  - *Screening: Not on any screening lists*
- ATSDR (1990) reported no studies of **reproductive** toxicity from exposure to silver or silver c**ompounds** were found for inhalation, oral, or dermal exposure.
- The Japanese NITE classification for reproductive toxicity is “classification not possible” based on no data (NITE, **2006).**
- There was no decrease of fertility in male rats exposed for life to drinking water containing 635-660 mg silver/day as silver chloride (Olcott, 1948).

**Developmental Toxicity incl. Developmental Neurotoxicity (D) Score (H, M or L):** DG

Conventional silver was assigned a score of Data Gap for developmental toxicity based on lack of mammalian data.

- Authoritative and Screening Lists
  - *Authoritative:*
    - *MAK - Pregnancy Risk Group D*
  - *Screening: Not on any screening lists*
- ATSDR (1990) reported no studies of developmental toxicity from exposure to silver or silver compounds were found for inhalation, oral, or dermal expo**s**ure.
- EPA (2009) evaluated developmental toxicity data in rats exposed to silver acetate (Danscher, 1981), however silver acetate is outside the scope of this paper.
- EPA (1993) evaluated developmental toxicity data in rats exposed to silver lactate monohydrate (Rungby et al., 1987), however silver lactate is outside the scope of this paper.

Non-mammalian data:

- The developmental toxicity of silver nano- and micro-sized particles was investigated in Drosophila egg development. Drosophila were exposed to 10 ppm of silver with particles sizes in the range of 20-30 nm, 100 nm, and 500-1200 nm. Dynamic light scattering and transmission electron microscope data indicated agglomeration of the nano-scale particles in water, such that the agglomerate sizes were significantly larger than the sizes reported for the primary particles.

**Analysis of silver particle size and exposure concentration on Drosophila larvae and pupae (data compiled from Gorth et al., 2011):**

| Particle size as purchased | Particle size peak(s) as determined by TEM | Particle size as determined by dynamic light scattering | Eggs that pupated after exposure to 10 ppm | % Pupae that emerged to adults after exposure to 10 ppm | % Pupae that emerged to adults after exposure to 100 ppm | % Pupae that emerged to adults after exposure to 100 ppm | Silver accumulation in adult Drosophila after exposure to 10 ppm |
| --- | --- | --- | --- | --- | --- | --- | --- |
| Controls | N/A | N/A | 59% ± 10% | 89% ± 25% | 59% ± 10% | 89% ± 25% | 0 ± 0.00091 µg |
| 20-30 nm | 18.7-37.84 nm, and 122.4-190.1 | 782 ± 379 nm | 47% ± 15% | 57% ± 48%* | 51% ± 15% | 13% ± 4%* | 385.64 ± 20.19 µg |
| 100 nm | 141.8-342 nm | 693 ± 114 nm | 59% ± 10% | 91% ± 19% | 30% ± 17%* | 5% ± 1%* | 69.03 ± 0.97 µg |
| 500-1200 nm | 164.2-531.2 nm | 508 ± 32 nm | 34% ± 12%* | 94% ± 52% | 5% ± 4%* | 0% ± 0% | 14.17 ± 0.17 µg |

*Statistically significant (P < 0.05)

Authors reported that the larger particles were more toxic to the Drosophila larvae when the exposure concentration was 10 ppm and 100 ppm. Whereas exposure to 10 ppm resulted in fewer pupae reaching adulthood for small particles sizes, this trend reversed when the dose was 100 ppm. Authors concluded nanosilver < 100 nm was less toxic to Drosophila than conventional sizes of silver > 100 nm (Gorth et al., 2011). [NSF notes several deficiencies in these conclusions: 1) the authors did not note the control substance, 2) whereas authors noted complete lethality at 100 ppm for 500-1200 nm sized silver particles, they apparently ignored that the larger particles were less toxic to pupae at 10 ppm even though they acknowledged higher accumulations for adults exposed to the smaller particle sizes; 3) it would have been informative if they had measured accumulation from pupae as opposed to those exposed only as adults; 4) they did not report the concentration(s) for the results of the dynamic light scattering values; 5) authors apparently did not examine changes in agglomeration and particle sizes when moving from 10 ppm to 100 ppm; 6) Authors did not report analysis of total survival to adulthood, but this can be estimated by multiplying % pupated x % to adults. At 10 ppm approximately 52.51% of controls reached adulthood, 26.79% of the 20-30 nm group, 53.69% for the 100 nm group, and 32.96% for the 500-1200 nm group. The authors’ rationale that toxicity was greatest for larger particle sizes at 10 ppm is incomplete].

- Lacoue-Labarthe et al. (2009) examined the effect of silver chloride on the phenoloxidase activation (PO) system in the embryo of the common cuttlefish *Sepia officinalis*. Eggs were exposed to dissolved trace levels of silver chloride (2 µg AgCl/L, or 1.2 µg Ag/L) from the time of spawning to 32 days (i.e. the end of organogenesis) and to 50 days (end of development). After 32 days, silver had accumulated in the eggs to 21.5 ± 5.8 ng, compared to 0.8 ± 0.3 ng in the controls, and at the end of 50 days had accumulated to 95.1 ± 40 ng, compared to 4.2 ± 1.6 ng in controls. PO activity was 2-, 2-, 3-, and 4-fold lower in embryos compared to controls at 32, 36, 40 and 44 days, respectively. However, a few hours before hatching, PO-like activity in controls decreased whereas it increased significantly in the exposed embryo (0.64 ± 0.03 vs. 0.44 ± 0.04 in the silver-exposed and control embryos, respectively. NSF notes the significance of these results to human developmental toxicity, or to developmental toxicity of other invertebrates is not known. Furthermore, the particle sizes were not characterized.

**Endocrine Activity (E) Score (H, M or L):** DG

Conventional silver was assigned a score of **Data Gap** for endocrine activity based on lack of data (note the only possible scores for Endocrine Activity in the GreenScreen™ are High, Moderate, or Data Gap – there is currently no option for Low hazard).

- Authoritative and Screening Lists
  - *Authoritative: Not on any authoritative lists*
  - *Screening: Not on any screening lists*
- Thyroid hormone signaling was measured in frog tissue using a cultured tail fin biopsy (C-fin) assay and *Rana catesbeiana* tadpoles. Whereas the metamorphosis of a tadpole into a frog is directly dependent on thyroid hormones (THs), premetamorphic tadpoles are free living and fully formed but do not produce measureable levels of THs, compared to prometamorphic tadpoles which synthesize increasing amounts that lead to the metamorphic changes. The C-fin assay maintains tissue complexity and biological replication, while allowing multiple chemical responses to be assessed from the same individual. Gene expression, measured as disruption of TH-action was assessed by measurement of mRNA abundance of transcripts encoding the TH-induced TH receptor β (TRβ) and TH-repressed Rana larval keratin type I (RLKI). Effects of cellular stress was also measured as changes in transcript levels of heat shock protein 30 (HSP30) and catalase (CAT). Alterations in the steady-state levels of these transcripts is suggestive of the potential for perturbing hormone-dependent postembryonic development and inducing cellular stress. Test substances included carboxy-coated nanosilver (1-10 nm), carboxy-coated quantum dots of cadmium telluride (1-10 nm), carboxy-coated nanozinc oxide (1-10 nm), **micro-silver (5-8 µm),** silver nitrate, and micro-cadmium telluride (<250 µm), in the presence and absence of 3,3’,5’-triiodothyronine (T_3_) using quantitative real-time polymerase chain reaction.

In the C-fin assay, half of the tail fin biopsies from each tadpole were exposed to solvent control or test substance to examine the effect on premetamorphic frog tadpole tissue. The other half of the biopsies were exposed to 10 nM T_3_, or 10 nM T_3_ plus test substance, to simulate a prometamorphic state and assess the effect of test substance exposure on the TH-induced response.

- Nanosilver (10 nM) decreased TRβ transcript levels 2-fold compared to controls, and RLKI transcript levels were decreased up to 2-fold at the two highest concentrations tested (5 nM and 10 nM). **Micro-silver exerted no significant effects on TRβ or RLKI transcripts levels in the presence or absence of T_3_. Silver nitrate showed little effect on** TRβ and RLK1 transcript levels, except for a 1.7-fold attenuation in the T_3_-dependent increase in TRβ transcript levels at the lowest silver nitrate concentration tested. Nanosilver did not cause any significant changes to the HSP30 and CAT transcript levels in both the absence and presence of T_3_. **Micro-silver showed no significant effects on the HSP30 or CAT transcripts.** Silver nitrate exposure elicited a 4-fold and 9-fold increase in HSP30 transcript levels at 0.6 and 6.0 µg/L, respectively (Hinter et al., 2010).

**Group II and II* Human Health Effects (Group II and II* Human)**

*Note: Group II and Group II* endpoints are distinguished in the v 1.2 Benchmark system (the asterisk indicates repeated exposure). For Systemic Toxicity and Neurotoxicity, Group II and II* are considered sub-endpoints. When classifying hazard for Systemic Toxicity/Organ Effects and Neurotoxicity endpoints, repeated exposure results are required and preferred. Lacking repeated exposure results in a data gap. Lacking single exposure data does not result in a data gap when repeated exposure data are present (shade out the cell in the hazard table and make a note). If data are available for both single and repeated exposures, then the more conservative value is used.*

**Acute Mammalian Toxicity (AT) Group II Score (vH, H, M or L): L**

Conventional silver was assigned a score of **Low** (high confidence) for acute mammalian toxicity based on EPA reported LD50’s for oral and dermal exposures to silver (II) oxide. The hazard score is reported as high confidence based on LD50 values reported in multiple reliable secondary sources.

- Authoritative and Screening Lists
  - *Authoritative: Not on any authoritative lists*
  - *Screening: Not on any screening lists*
- **The EPA RED (1993) reported an oral LD50 > 5000 mg/kg, an inhalation LC50 as N/A [not applicable], and a dermal LD50 at >2000 mg/kg,** based on powdered sildate (7.5 g dispersed in 250 mL distilled water). [Powdered Sildate is silver (II) oxide, which was tested in crystal form, size not specified, <http://www.tetrasilver1.com/>. Sildate is registered by N. Jonas & Company, Inc., EPA Reg. # 3432-64].
- Japanese NITE classification for acute toxicity **oral** is “not classified” based on rat oral LD50 > 5000 mg/kg as reported in DFGOTvol.19 (2003) (NITE, 2006). The cross-reference for DFGOTvol.19 could not be found.
- **Rat oral LD50 was reported at >2000 mg/kg-bw,** conducted per OECD 401 using silver powder of <40 µm particle size (Johnson Matthey, 1993 (unpublished report) as cited in IUCLID, 2000).
- Japanese NITE classification for acute toxicity **dermal** is “not applicable” based on a reported LD50 >2000 mg/kg as cited in HSDB (2003) (NITE, 2006). It should be noted the values reported in HSDB correspond to those reported in EPA RED (1993).
- Japanese NITE classification for acute toxicity **inhalation** - gas is “not applicable” based on the GHS definition for a solid; for vapor and dust/mist “classification not possible” based on no available data (NITE, 2006).
- ATSDR (1990) reports no studies were located regarding death in humans or animals after inhalation, oral, or dermal exposure to silver or silver compounds. Data was summarized for colloidal silver and silver nitrate.
- ACGIH (2001) noted an **oral LD50 for AgO was 2820 mg/kg**.
- ACGIH (2001) further reported acute toxicity for oral and intraperitoneal to silver nitrate in rodents, and case studies of respiratory effects to silver fumes, however, silver nitrate and silver fumes were determined to be outside the scope of this GreenScreen.

**Systemic Toxicity/Organ Effects incl. Immunotoxicity (ST)**

**(ST-single) Group II Score (single dose: vH, H, M or L);** DG

Conventional silver was assigned a score of **Data Gap** for systemic toxicity/organ effects based on single exposure, due to insufficient data. It may be noted the Japanese NITE classification and ATSDR comments on acute inhalation toxicity have been discounted (see Drake and Hazelwood (2005) below).

- Authoritative and Screening Lists
  - *Authoritative: Not on any authoritative lists*
  - *Screening:*
    - *Japan - GHS - Specific target organs/systemic toxicity following single exposure - Category 1*
- The Japanese NITE classification for specific target organ / systemic toxicity following single exposure is “Category 1, danger, cause damage to organs (respiratory)” based on reports that lung disorders with pulmonary edema developed after exposure to heated metallic silver fumes for 4 hours as reported by ACGIH (2001), and that irritation to the airways develops after occupational exposure to the dust as reported by ATSDR Tox FAQs (1997) (NITE, 2006).
- ACGIH (2001) reported case studies of respiratory effects to silver fumes, however, silver fumes was determined to be outside the scope of this GreenScreen. (Forycki et al., 1983; Drake and Hazelwood 2005; Rosenman et al., 1979, 1987; Pifer et al., 1989; Barrie and Harding, 1947; Perrone et al., 1977; Forycki et al.,1983) ATSDR FAQ’s for silver discuss hazards associated with inhalation exposure in only a single statement: “Exposure to high levels of silver in the air has resulted in breathing problems, lung and throat irritation, and stomach pains” (ATSDR, 1999).
- No acute inhalation studies in humans or animals, of duration < 14 days exposure, were identified (ATSDR, 1990).

**(ST-repeat) Group II* Score (repeated dose: H, M, L):** DG

Conventional silver was assigned a score of **Data Gap** for systemic toxicity/organ effects based on insufficient data. It should be noted the oral RfD (IRIS, 2003) is based on argyria in humans, a cosmetic effect resulting from oral exposure to silver nitrate, silver acetate, and silver arsphenamine, which are outside the scope of this paper. There is no inhalation RfC, based on lack of data.

- Authoritative and Screening Lists
  - *Authoritative: Not on any authoritative lists*
  - *Screening:*
    - *Japan - GHS - Specific target organs/systemic toxicity following repeated exposure - Category 1*
- The Japanese NITE classification [ID 106] for specific target organ / systemic toxicity following repeated exposure is “Category 1, danger, causes damage to organs (eye) through prolonged or repeated exposure; causes damage to organs ( respiratory: inhalation) through prolonged or repeated exposure.” This is based on reports of argyria as reported by ACGIH (2001) and Patty’s Industrial Hygiene (5^th^, 2001), but as the description of dysfunction of decreased night vision (ATSDR ToxFAQs (1997), it was classified into Category 1 (eye). [Note the night vision effects were based on complaints that were not substantiated with testing, see ATSDR 1990 below. Also, the most recent ATSDR ToxFAQ’s (1999) no longer mention a vision hazard]. It was classified into Category 1 (respiratory: inhalation) with the description that it became bronchitis by deposition to the lungs by prolonged inhalation of a dust as reported in Patty’s Industrial Hygiene (5^th^, 2001) and HSDB (2003) (NITE, 2006). [Note Patty’s is a secondary reference, and HSDB no longer mentions bronchitis. This information is insufficient to assign a hazard].

Oral

- Case histories of oral exposure to silver generally result in gray pigmentation of the skin in sun-exposed regions. Although granular deposits are distributed throughout the dermis, they tend to concentrate in the basement membrane and elastic fibers surrounding sweat glands (Bleehan et al., 1981 (occupational exposure, precise form unknown and mixed with other metals); MacIntyre et al., 1978 (anti-smoking lozenge containing silver acetate and ammonium chloride which precipitates silver chloride), as summarized in ATSDR, 1990). It is important to note that while deposition of silver from ingestion of silver nitrate and silver chloride has also been observed in animals, the skin discoloration has not been observed (Olcott, 1948, and Walker, 1971, as summarized in ATSDR, 1990).
- Note the following ATSDR studies were not relevant as they were based on colloidal or soluble silver [no information was found for conventional low-solubility non-nano silver]:
  - Deaths occurred in rats exposed to 2589 ppm (362 mg Ag/kg/day) colloidal silver in drinking water for 2 weeks. Decreased body weight was observed at 181 mg Ag/kg/day (Dequidt et al., 1974, as summarized in ATSDR, 1990).
  - Sluggish behavior was reported in mice exposed to 95 ppm (18 mg Ag/kg/day) silver nitrate in the drinking water for 125 days (Rungby and Danscher, 1984, as summarized in ATSDR, 1990).
  - Decreased body weight was reported in rats exposed to 1587 ppm (222.2 mg Ag/kg/day) silver nitrate in drinking water for 37 weeks (Matuk et al., 1981, as summarized in ATSDR, 1990).

Dermal

- Granular deposits were observed in the conjunctiva and cornea of the eyes of 20 out of 30 workers occupationally exposed to silver nitrate and silver oxide in the study of Rosenman et al. (1979). Subjective determination of the degree of silver deposition in the conjunctiva correlated with the duration of employment (ATSDR, 1990). The amount of deposition in the eyes was correlated with reports of changes in skin color and decreased night vision, but tests of night vision did not detect significant differences in impairment (Pifer et al., 1989, as summarized in ATSDR, 1990).
- Skin and ocular burns have been reported from worker exposure to silver nitrate [concentration not specified] (Moss et al., 1979; Rosenman et al., 1979, as summarized in ATSDR, 1990), but not for other forms of silver.
- No studies were located regarding dermal or ocular effects in animals following inhalation exposure to silver or silver compounds (ATSDR, 1990).
- No studies were located regarding respiratory, cardiovascular, gastrointestinal, hematological, musculoskeletal, hepatic, renal, or ocular effects in humans or animals after dermal exposure to silver or silver compounds (ATSDR, 1990).

Inhalation

- No repeated dose inhalation studies were identified from exposure to silver or silver compounds in humans or animals, of duration > 14 days (ATSDR, 1990).
- Although occupational studies report that 25 out of 30 workers complained of upper respiratory irritation (sneezing, stuffiness, and running nose or sore throat) at some time during their employment, with 20 out of 30 complaining of cough, wheezing, or chest tightness. Chest radiograms and results of clinical examination of respiratory function were predominantly normal with no demonstrated relationships between abnormalities and duration of employment. Employment durations ranged from less than one to greater than ten years (Rosenman et al., 1987, as summarized in ATSDR, 1990).
- **Abdominal pain** has been reported by workers exposed to silver nitrate and **silver oxide** in the workplace. This symptom correlated significantly with blood silver levels. Exposure levels were estimated to be between 0.039 and 0.378 mg Ag/m^3^, but no information on chemical form or particle size was provided (Rosenman et al., 1979, as summarized in ATSDR, 1990). [Abdominal pain is not necessarily toxicologically significant].
- In a study by Pifer et al. (1989), silver reclamation workers chronically exposed to insoluble silver compounds, mostly silver halides, exhibited a marginal decrease in red blood cell count, and an increase in mean corpuscular volume, however the toxicological significance is unclear (ATSDR, 1990).
- In a study that measured the liver enzymes alanine amino transferase, aspartate amino transferase, gamma glutamyl transferase, and alkaline phosphatase, there were no significant differences between workers exposed to silver and insoluble silver compounds compared to those with no history of silver exposure (Pifer et al., 1989, as summarized in ATSDR, 1990).
- No studies were located regarding health effects for humans or animals after inhalation exposure to silver or silver compounds for immunological effects, neurological effects, developmental effects, reproductive effects, genotoxic effects, or cancer (ATSDR, 1990).

**Neurotoxicity (N)**

**(N-single) Group II Score (single dose: vH, H, M or L)** DG

Conventional silver was assigned a score of **Data Gap** for neurotoxicity based on lack of data.

- Authoritative and Screening Lists
  - *Authoritative: Not on any authoritative lists*
  - *Screening: Not on any screening lists*
- No studies of neurotoxicity from silver exposure were found for inhalation, oral, or dermal exposure (ATSDR, 1990).
- The following citations evaluated neurotoxicity after a single dose of soluble silver salts therefore were not in the scope of this report:
  - Rungby (1987) Explored the neurotoxic effect of subcutaneous injections of silver lactate on the volumes of the components of the developing rat hippocampus.
  - Rungby (1984). Measured hypoactivity in mice exposed to silver lactate via i.p. injection and to silver nitrate in drinking water.
  - Rungby (1983). Examined localization of exogenous silver in brain and spinal cord of rats exposed to a single i.p. injection of silver lactate.

**(N-repeat) Group II* Score (repeated dose: H, M, L):** DG

Conventional silver was assigned a score of **Data Gap** for neurotoxicity based on lack of data.

- Authoritative and Screening Lists
  - *Authoritative: Not on any authoritative lists*
  - *Screening: Not on any screening lists*
- No studies of neurotoxicity from silver exposure were found for inhalation, oral, or dermal exposure (ATSDR, 1990).
- The following citation evaluated neurotoxicity after repeated doses of soluble silver salts therefore was not in the scope of this report:
  - Rungby (1984). Measured hypoactivity in mice exposed to silver lactate via i.p. injection and to silver nitrate in drinking water.

**Skin Sensitization (SnS) Group II* Score (H, M or L):** *L*

Silver was assigned a score of Low (low confidence) for skin sensitization based on centuries of use with only very limited anecdotal reports of sensitization, which would not qualify for classification per GHS criteria. Confidence is low due to lack of standardized test data on conventional silver. Note the transformation product silver thiosulfate tested negative in a guideline study.

- Authoritative and Screening Lists
  - *Authoritative: Not on any authoritative lists*
  - *Screening:*
    - *Japan - GHS - Skin sensitizer - Category 1*
- Three case studies report sensitization potentially from exposure to silver. First was to silver cyanide after 6 months exposure, second to silver or other substances in radiographic processing solutions after 10 years exposure, and third to silver in dental amalgam [“amalgam” implies a blend with other metals] after 20 years. In each case the quantity of silver was not known, and no other studies of sensitization were identified for humans or animals (ATSDR, 1990). [This is insufficient to assign a hazard].
- The Japanese NITE classification for skin sensitization is “Category 1, warning, may cause allergic skin reaction” based on descriptions that powder exposure causes allergic contact dermatitis as reported by ACGIH (2001) and that contact to accessories containing silver produced allergic reactions as report in Patty’s Industrial Hygiene (5th, 2001) (NITE, 2006). [ACGIH (2001) is addressed below, and Patty’s (2001) is a secondary reference. Neither of these it suitable to assign a hazard].
- ACGIH (2001) notes “skin contact with silver compounds has been found to cause mild allergic reactions, such as rash, swelling, and inflammation, in some people”. [No further details were reported and this is insufficient to assign a hazard].
- A sensitization study was identified for ionic silver (http://www.purebio.com/technology/file/7/Dermal_Sensitization.pdf). This data was not considered further because ionic silver is outside of the scope of this report.

**Respiratory Sensitization (SnR) Group II* Score (H, M or L):** DG

Conventional silver was assigned a score of DG for respiratory sensitization based on lack of data.

- Authoritative and Screening Lists
  - *Authoritative: Not on any authoritative lists*
  - *Screening: Not on any screening lists*
- The Japanese NITE classification for respiratory sensitization is “classification not possible” based on no data (NITE, 2006).

**Skin Irritation/Corrosivity (IrS) Group II Score (vH, H, M or L): L**

Conventional silver was assigned a score of **Low** (high confidence) for skin irritation/corrosivity based on results from an OECD 404 test. While some study details are lacking, skin irritation is not generally associated with silver occupational exposure. The hazard score is based on a well-documented GLP-study and therefore is reported as high confidence.

- Authoritative and Screening Lists
  - *Authoritative: Not on any authoritative lists*
  - *Screening: Not on any screening lists*
- The Japanese NITE classification for skin corrosion / irritation of silver is “not classified” based on reported “slightly irritating” to rabbits as cited in IUCLID (2000) (NITE, 2006).
- Silver powder of <40 µm particle size was slightly irritating when tested per OECD 404 under semi occlusive conditions on New Zealand White rabbits. Exposure was for 4 hours and observations where made approximately 1 hour following the removal of the patches, and 24, 48 and 72 hours later, the test sites were examined. 24 hours after removal of the patches a mean Draize score of 0.33 was observed. Irritation was fully reversible within: 72 hours after removal of the patches (ECHA 2012).

**Eye Irritation/Corrosivity (IrE) Group II Score (vH, H, M or L): M**

Silver was assigned a score of **Moderate** (high confidence) for eye irritation/corrosivity based on an OECD 405 study. The hazard score is based on a well-documented GLP-study and therefore is reported as high confidence.

- Authoritative and Screening Lists
  - *Authoritative: Not on any authoritative lists*
  - *Screening:*
    - *Japan - GHS - Serious eye damage / eye irritation - Category 2B*
- The Japanese NITE classification for serious eye damage / eye irritation is Category 2B, warning, causes eye irritation, based on mild irritation in rabbits with recovery in 48 hours as reported in IUCLID (2000) (NITE, 2006).
- Silver powder of <40 µm particle size was **slightly irritating** to rabbit eyes when tested per OECD 405 (Johnson Matthey, 1993 unpublished report IUCLID, 2000). [Additional details including the species were not reported, however the default species for this method is the rabbit. This data corresponds to that published for Sildate on the Tetrasilver1 website. Sildate is a crystal form of silver (II) oxide, size not specified, <http://www.tetrasilver1.com/>. Sildate is registered by N. Jonas & Company, Inc., EPA Reg. # 3432-64].

**Ecotoxicity (Ecotox)**

Colloidal elemental silver is listed with German FEA – Substances Hazardous to Water (VwVwS): Class 3 Severe Hazard to Waters. [This classification can be based on mammalian or aquatic toxicity, combined with persistence and bioaccumulation potential. VwVwS is a screening list per the GreenScreen v1.2 List Translator, and can span all of the GreenScreen Benchmark scores. No further characterization of the silver by particle size is specified in the German listing, and no further details regarding this evaluation were provided].

**Acute Aquatic Toxicity (AA) Score (vH, H, M or L): vH**

Conventional silver was assigned a score of **very High** (high confidence) for acute aquatic toxicity based on GHS Category 1 classification in daphnia (EC/LC50 < 1 mg/L). The hazard score is based on results from numerous studies and therefore is reported as high confidence. Please note that the actual aquatic toxicity of silver will vary greatly based on the environmental conditions. See the discussion in the introduction under "Justification for Chemical Surrogates" for more information.

- Authoritative and Screening Lists
  - *Authoritative: Not on any authoritative lists*
  - *Screening:*
    - *New Zealand - GHS - 9.1A (algal) - Very ecotoxic in the aquatic environment*
    - *New Zealand - GHS - 9.1A (crustacean) - Very ecotoxic in the aquatic environment*
    - *New Zealand - GHS - 9.1A (fish) - Very ecotoxic in the aquatic environment*
- The Japanese NITE classification for acute aquatic hazards is “classification not possible” based on insufficient data (NITE, 2006).
- A study by Kim et al. (2011) examined EC50 and LC50 values for *Daphnia magna* and *Oryzias latipes* (Japanese Medaka) exposed to silver nanoparticles. The study also attempted to confirm that the resulting toxicity is due to Ag^+^ ion released from the silver nanoparticles. An acute immobilization test for *D. magna* was carried out in accordance with **OECD 202**, and the acute toxicity test for *O. lapites* was carried out in accordance with **OECD 203**. Two suspensions of AgNP powder were used in the study: one with a mean particle diameter of 60 nm and the other with a **mean particle diameter of 300 nm.** In addition, the two organisms were also exposed to AgNO_3_ solution to compare the effects of AgNP to those of ionic silver. The author noted that rapid aggregation and agglomeration occurred in suspension, but many particles with diameters of < 100 nm were present in the water column at 48 hours, indicating that the potential for exposure to silver nanoparticles was present for at least an exposure of 48 hours.

The EC50s were as follows:

48 h EC50, daphnia, AgNO_3_ = 0.5 mg Ag/L (95% CI = 0.4–0.6).

48-h EC50, daphnia, AgNP 60 nm = 1.0 mg Ag/L (95% CI = 0.1–.3)

**48-h EC50, daphnia, AgNP 300 nm = 1.4 mg Ag/L (95% CI = 0.3–2.1)**

96 h LC50, latipes, AgNO_3_ = 21 mg Ag/L (95% CI = 15–30)

96 h LC50, latipes, AgNP 60 nm = 28 mg Ag/L (95% CI = 23–34)

**96 h LC50, latipes, AgNP 300 nm = 67 mg Ag/L (95% CI = 45– 08)**

- “The sorption efficiency of the synthesized sorbent was checked by spiking 0.5 mg/L Ag^+^ standard solution. The result, given in Table 1, showed that most silver ion was absorbed to the sorbent and very little silver was detected in the sample passed through the column. The sorption efficiencies of the column for 300-nm AgNP suspension were 98.8, 99.7 and 99.7%, which were lower than those for Ag+ standard solution. The calculated exposure concentrations of the column-passed 300 nm AgNP suspensions were 3.22, 0.34, and 0.72 mg/L in three experiments, respectively. Although the 48 h EC50 of 300 nm AgNP in the previous toxicity test was 1.4 mg/L, immobilization was not seen in any of the column-passed 300 nm AgNP suspensions at 48 h.”
- The authors concluded that AgNPs were acutely lethal to *D. magna* and *O. latipes* and would be classified as GHS category 1 for acute aquatic toxicity. The authors also noted that the EC50s/LC50s were similar to AgNO_3_, suggesting that the toxicity of AgNP suspensions is caused by Ag^+^ particles. The sorbent test described in the previous paragraph exposed daphnia to AgNP that excluded Ag^+^, and immobilization of *daphnia* did not occur in the 300 nm 0.00322 µg/L AgNP solution lacking Ag^+^. The authors stressed the need to examine the aggregates/agglomerates in the suspension of nanoparticles, which are believed to be non-ionic and non-toxic to aquatic life (Kim et al., 2011).
- Gaiser et al (2011) examined acute 96-hour and chronic 21-day exposure of *Daphnia magna* neonates to silver particles. Nano and micro silver particles of size 35 nm and **0.6 – 1.6 μm, respectively**, were used. However, in the reconstituted hard water used for D. magna culture and exposures, rapid aggregation occurred, and **a high degree of polydispersity was evident. Mean sizes of particles in reconstituted hard water were 588 nm for nano-Ag**, **and 811 nm for micro-Ag.**
- *Daphnia* neonates were exposed to 20 mL of 0, 0.01, 0.1, 1 and 10 mg/L of all particle types under semi-static conditions for acute exposures (96 h). For chronic exposures (21 d), the particle concentrations were based on the results of the acute toxicity studies and were 0, 0.001, 0.005, 0.01 and 0.05 mg/L. All exposure groups consisted of ten organisms each. *D. magna* were assessed for survival and molting (shedding of carapace) daily.
- “Ag particles caused mortality of *D. magna* neonates in a concentration-dependent manner. **Nano-Ag particles exhibited higher toxicity than the micron-sized particles:** 100% mortality occurred for both 10 and 1 mg/L of nano-Ag, and 56.7 ± 23.3% mortality at 0.1 mg/L for the nanoparticles. Treatment with micro-Ag resulted in a lower overall toxicity threshold with 100% mortality at 10 mg/L, 80 ± 20% mortality at 1 mg/L, and no significant toxicity at 0.1 mg/L.” The inferred 96-hour LC50 for the nanoparticles was approximately 0.1 mg/L, and **the inferred** **96-hour LC50 for the microparticles is in the range of 0.1 – 1 mg/L.** The middle and high dose groups also showed reduced moulting frequency and reduced growth over the 96-hour period compared with controls.
- “Compared with the control daphnids, neonates exposed to the lowest concentration of nano-Ag had a normal appearance. However, approximately half of the neonates exposed to 0.1 mg/L of nano-Ag showed clear signs of the toxic effects, including significantly reduced body size, changes in feeding and/or metabolism assessed by lack or reduced amounts of algae (green colour) in the digestive tract (66.7% of examined neonates), lack or reduced amounts of the brown lipid storage droplets usually surrounding the intestine (53.3% of examined neonates), and lesions (40% of examined neonates). *D. magna* exposed to micro-Ag up to 1 mg/L, while significantly reduced in size (1 mg/L only), did not show abnormalities in the contents of their intestine, the amount of lipid storage droplets, or lesions, even at 1 mg/L”.
- There was consistent but low mortality throughout the 21-day exposures, with mortality rates ranging from 0 – 30% across the dose range. There was a lack of dose-dependency, with the 30% mortality rate occurring at the low dose (0.001 mg/L).
- **48-hr LC50 in *Daphnia magna* for micro-sized silver (600-1600nm) was < 1 mg/L** (based on 80% mortality at 1 mg/L). Test method was EPA-821-R-02-012, Methods for measuring the acute toxicity of effluents and receiving waters to freshwater and marine organisms (Gaiser, 2012).
- Survival rates in *D. magna* exposed to **micro-Ag (600-1600 nm)** for 96 h were reported at 96.67 ± 3.33% at 0.01 mg/L, 86.67 ± 6.67% for 0.1 mg/L, 20 ± 20% at 1 mg/L, and 0% at 10 mg/L (Gaiser et al., 2011 and 2009). **The 96-hr LC50 in *D. magna* can be inferred to be in the range of 0.1-1 mg/L.**
- 7-day LC50 values in rainbow trout were > 100 mg/L for silver thiosulfate, silver chloride, and silver sulfide (Wood et al., 1994, 1996b; Hogstrand et al., 1996, as cited in WHO, 2002).
- The acute toxicity of nanosilver powder (25.4nm) suspensions was evaluated in *Ceriodaphnia dubia* and *Pseudokirchneriella subcapitata*, each exposed in three different types of water. The water types varied in dissolved organic carbon (DOC) content, pH, and various ion concentrations. Mixtures were shaken for 1 week then filtered (1.6 µm) to remove large aggregates. The obtained filtrates were then used to determine total concentrations of Ag by ICP-AES. The exposure method was **EPA-821-R-02-013, Short-term methods for estimating the chronic toxicity of effluents and receiving waters to freshwater organisms.** *C. dubia* were exposed at 25°C under constant aeration with a photoperiod of 16:8 light:dark, for 48 hours under static conditions. The endpoint was mortality and/or immobilization. *P. subcapitata* was incubated for 96 hours at room temperature under controlled light and shaken twice daily. Authors observed images from scanning electron microscopy which showed the silver apparently coated by the DOC within the ACT water, and speculated this serves a protective function resulting in lower acute aquatic toxicity under the test conditions. Comparatively, the nanosilver suspended in the SPG (Spring fed) water showed large and small aggregates with no evidence of organic matter coating (McLaughlin et al., 2012). Results are summarized as follows:

**Summary of acute aquatic toxicity study results (derived from Mclaughlin et. al, 2012)**

| Water designation | Water type | Average diameter of the suspended nAg (nm) | Zeta potential (mV) | *C. dubia* 48hr EC50 (mg/L) | *P. subcapitata* 96h IC50 (mg/L) |
| --- | --- | --- | --- | --- | --- |
| ACT | Wetland water (high DOC) | 76.8 | -28.8 | 221 | 1600 |
| SPG | Spring fed water (**low DOC** and moderate ionic strength) | **192** | -12.7 | **0.433** | 22.6 |
| CM | Culture medium (per EPA method) | **174** | -24.8 | N/A | **4.61** |
| MHW | Moderately hard water (traditional growth media) | **395** | -4.38 | **0.482** | N/A |

**Summary LC/EC50’s:**

| **Species** | **AgNP form** | **EC50** | **Reference** |
| --- | --- | --- | --- |
| **Invertebrates** | | | |
| *Daphnia magna* | Powder 300 nm | 1.4 mg/L, 48h | Kim et al., 2011 |
| *Daphnia magna* | 0.6-1600 nm | 0.1-1 mg/L, 96h | Gaiser et al., 2011 |
| *Daphnia magna* | 0.6-1600 nm | <1 mg/L, 48h | Gaiser et al., 2012 |
| *Ceriodaphnia dubia* | Powder AgNP 395 nm | 0.433-0.482 mg/L, 48h (in spring fed and moderately hard water) | McLaughlin et al., 2012 |
| **Fish** | | | |
| *Oryzias latipes* (Japanese Medaka) | Powder 300 nm | 67 mg/L, 96 h | Kim et al., 2011 |
| **Algae** | | | |
| *P. subcapitata* | Powder AgNP 395 nm | 4.61 mg/L | McLaughlin et al., 2012 |

**Chronic Aquatic Toxicity (CA) Score (vH, H, M or L):** *vH*

Conventional silver was assigned a score of **very High** (low confidence) for chronic aquatic toxicity based on toxicity results summarized below and the acute toxicity results summarized above. GHS allows extrapolation from acute to chronic for this parameter, therefore effects in both daphnia and algae would result in a Category 1 assignment. The hazard score is based on equivocal test results and extrapolations from acute study results and therefore is reported as low confidence. Please note that the actual aquatic toxicity of silver will vary greatly based on the environmental conditions. See the discussion in the introduction under "Justification for Chemical Surrogates" for more information.

- Authoritative and Screening Lists
  - *Authoritative: Not on any authoritative lists*
  - *Screening:*
    - *New Zealand - GHS - 9.1A (algal) - Very ecotoxic in the aquatic environment*
    - *New Zealand - GHS - 9.1A (crustacean) - Very ecotoxic in the aquatic environment*
    - *New Zealand - GHS - 9.1A (fish) - Very ecotoxic in the aquatic environment*
- The Japanese NITE classification for chronic aquatic hazards is “classification not possible” based on lack of data (NITE, 2006).
- The New Zealand classification for fish, crustaceans, and algae is 9.1A “very ecotoxic to the aquatic environment,” which is equivalent to GHS category 1, and carries risk phrase R-50/53 (NZ EPA, not dated).
- 21-day LC50 in *Daphnia magna* was > 0.1 mg/L (based on 20% mortality at 0.1 mg/L). **Interestingly, toxicity did not appear to follow a dose-response curve in this test. Nominal concentrations were 0.01, 0.1, 1 and 10 mg/L. The 0.1 mg/L was the most toxic with 20% mortality, whereas the 0.01, 1 and 10 mg/L exposures resulted in only 10% mortality** (Gaiser, 2012).
- There was no effect on survival in *D. magna* exposed to macro-Ag (600-1600 nm) at concentrations of 0, 0.001, 0.005, 0.01, and 0.05 mg/L for 21 days. A trend of decreased moulting was observed however, with those treated at 0.001 and 0.005 mg/L had 709% and 715% cumulative moulting, at 0.01 mg/L 679% moulting, and at 0.05 mg/L 622% moulting (Gaiser, 2011). **A 21-day LC50 can be inferred at > 0.05 mg/L.** An EC50 was not calculated, and moulting is not a traditional endpoint in GHS criteria.

**Environmental Fate (Fate)**

**Persistence (P) Score (vH, H, M, L, or vL): vH**

Conventional silver was assigned a score of **very High** (high confidence) for persistence. It is expected to be persistent because it is an element which can be transformed but not degraded. The hazard score is based on known physical properties of the chemical and therefore reported with high confidence.

- Authoritative and Screening Lists
  - *Authoritative: Not on any authoritative lists*
  - *Screening:*
    - *EC - CEPA DSL - Persistent*
- The New Zealand classification for rapid degradation is ND (NZ EPA, not dated), presumably “no data”.

**Bioaccumulation (B) Score (vH, H, M, L, or vL): L**

Conventional silver was assigned a score of **Low** (high confidence) for bioaccumulation based on weight of evidence, including rapid excretion in humans and dogs, low BAF’s and BCF’s in various aquatic species, and a low solubility which generally corresponds with low potential for bioaccumulation. The hazard score is based on results from numerous studies and therefore is reported as high confidence.

- Authoritative and Screening Lists
  - *Authoritative: Not on any authoritative lists*
  - *Screening: Not on any screening lists*
- Uptake from water, depuration and tissue distribution of 110Ag in a freshwater fish, Cyprinus carpio reported BCF at a steady state was ~70 (ECHA 2012).
- As summarized above, a Comet assay along with analysis for bioaccumulation was performed in the polychaete, *Nereis diversicolor.* The worms were exposed to nominal concentrations of nanosilver (<100 nm, 99.5% metals basis, coated by 0.2 wt% PVP), **micro-silver** (2-3.5 µm, ≥99.9% trace metal analysis), and ionic silver (AgNO3) at 0, 1, 5, 10, 25, and 50 ug Ag/g dry weight sediment for 10 days. The presence of highly crystalline material was observed in nanosilver, suggesting the presence of large silver particles (aggregates, 20-200 nm, average 162 nm). For microsilver, 5-10% of non-crystalline material was observed, suggesting it was not as pure as described by the manufacturer (i.e. <99.9% purity), and had both micro and nano-sized particles (8nm – 3 µm). **Reported silver body burdens for the** nano-, **micro-,** and ionic-silver **treatments were** 8.56, **6.92,** and 9.86 **µg/g dw,** respectively**. These values correspond to BAF factors of** 0.17, **0.14,** and 0.20, respectively (Cong et al., 2011).
- Absorption was measured in a **dog** that was exposed to 0.5 µm silver by inhalation for 6 hours. Authors reported 3.1% (0.8 µg) of the deposited silver was dissolved, transported out of the lungs, and found in the liver and blood. They estimated the absorption rate was 1 µg/cm^2^/day. Clearance from the lung to blood was reported to be triphasic with half-lives of 1.7, 8.4 and 40 days. They also reported that distribution to tissues other than the lungs was the same at 6 hours as at 225 days, with 77% being found in the liver (Phalen and Morrow, 1973, as summarized in ATSDR, 1990).
- Olcott (1948) reported wide silver distribution in **rats** following ingestion of silver chloride in the presence of sodium thiosulfate, and also as silver nitrate in drinking water. Concentrations were observed in the tissues of the reticuloendothelial system in the liver, spleen, bone marrow, lymph nodes, skin, and kidney. Silver was also distributed to other tissues including the tongue, teeth, salivary glands, thyroid, parathyroid, heart, pancreas, gastrointestinal tract, adrenal glands, and brain. Within these tissues, advanced accumulation of silver was found in the basement membrane of the glomeruli, walls of the blood vessels between the kidney tubules, portal vein, and other parts of the liver, choroid plexus of the brain, choroid layer of the eye, and in the thyroid gland. [Rate of excretion and bioaccumulation were not addressed].
- East et al. (1980) and Macintyre et al. (1978) reported approximately 18-19% of a single oral dose of silver acetate was retained in the body of a **human** 8-30 weeks after exposure. Furchner et al. (1968) noted this is 10% greater than that retained in dog tissues 20 weeks after a single oral dose (ATSDR, 1990).
- Bioaccumulation of metallic silver is unlikely as it is insoluble in water (Johnson Matthey, plc, as cited in IUCLID, 2000).
- A **BAF of 0.18 for silver was reported in Oligochaete (Lumbriculus Variegatus)** (an aquatic worm). This was a non-GLP and non-guideline study, sediment samples were spiked with silver sulfide, 300 worms were exposed in fresh water for 28 days (Hirsch, 1997 as cited in ECHA, 2012).
- A **BCF of 170 is reported for silver in the Mediterranean echinoid *Paracentrotus lividus*** [sea urchin] after exposure for 28 days. The silver appears to have been metallic but is not further characterized with regards to size, the presence of coatings, stabilizers, or other additives (Warnau et al., 1996 as cited in ECHA, 2012).
- A **BCF of 44.3 L/kg was reported in *Oligocuttus maculosus*** (Tidepool sculpins) after exposure for 21 days to concentrations up to 50.2 ± 0.7 mg/L Ag. This study was nearly conforming to OECD guideline305, and non-GLP. [The silver was not further characterized with regards to size, the presence of coatings, stabilizers, or other additives (Webb and Wood, 2000 as cited in ECHA, 2012).

**Physical Hazards (Physical)**

**Reactivity (Rx) Score (vH, H, M or L): L**

Conventional silver was assigned a score of **Low** (high confidence) for reactivity based on Dutch government research (RIVM, 2009), Japanese NITE classification and an MSDS for bulk metallic silver (ESPI Metals, 2011). The confidence level is high based on multiple lines of evidence suggesting low reactivity for bulk silver.

- Authoritative and Screening Lists
  - *Authoritative: Not on any authoritative lists*
  - *Screening: Not on any screening lists*
- Mechanical impact on powders may result in explosion, although bulk silver is not explosive (RIVM, 2009- no further information provided).
- Several compounds of silver are potential explosion hazards: silver oxalate (Ag_2_C_2_O_4_) decomposes explosively when heated; silver acetylide (Ag_2_C_2_) is sensitive to detonation on contact; and silver azide (AgN_3_) detonates spontaneously under certain circumstances (Smith & Carson, 1977 as cited in WHO, 2002). Such materials are outside of the scope of the current assessment.
- An MSDS notes the powder form of metallic silver has an HMIS reactivity rating of 1 (Slight hazard – materials which are normally stable, but can become unstable at high temperatures), and the solid form has an HMIS flammability rating of 0 (Minimal hazard – materials which are normally stable even under fire conditions, and which will not react with water) (ESPI Metals, 2011).
- Japanese NITE classification for silver is “not applicable” based on “no chemical groups associated with explosive properties present in the molecules” (NITE, 2006).

**Flammability (F) Score (vH, H, M or L): L**

Conventional silver was assigned a score of **Low** (high confidence) for flammability based on the analytical test results published in ECHA, 2012. Although the WHO (2002) and an MSDS in the public literature (ESPI Metals, 2011) indicate a concern for the powder form, the data used to support this concern is not available. Note an HMIS flammability rating of III is equivalent to GHS 2 or 3, which would translate to GreenScreen moderate or high hazard, respectively.

- Authoritative and Screening Lists
  - *Authoritative: Not on any authoritative lists*
  - *Screening: Not on any screening lists*
- Metallic silver powder (particle size was 90% > **0.5 µm**, based on D10 = 0.5 µm, D50 = 2.3 µm, and D90 = 83.4 µm) was determined to be nonflammable according to the EU Method A.10 (Flammability (Solids) (ECHA, 2012).
- **Silver is not combustible, except as powder** (WHO, 2002).
- An MSDS notes the ***powder* form of metallic silver has an HMIS flammability rating of 3 (Serious hazard - materials capable of ignition under almost all normal temperature conditions),** and the *solid* form has an HMIS flammability rating of 0 (Minimal hazard – materials that will not burn) (ESPI Metals, 2011).
- HMIS and NFPA flammability rating of 0 (Sigma Aldrich, 2015).
- Japanese NITE classification is “not applicable” for silver as a gas and liquid, and “not classified” for the solid form based on “non-combustible” per the ISCS, 1997 (NITE, 2006).

**References**

Agency for Toxic Substances and Disease Registry (ATSDR), U.S. Public Health Service. 1990. Toxicological profile for silver.

Asakura, K., S. Hiroshi, M. Chiba, M.Okamoto, K. Serizawa, M. Nakano, and K. Omae. 2009. Genotoxicity studies of heavy metals: Lead, Bismuth, Indium, Silver and Antimony. J Occup Health, 51:498-512.

American Conference of Governmental Industrial Hygienists (ACGIH). 2001. Documentation of the threshold limit values and biological exposure indices. Supplement to the 6th edition.

Agency for Toxic Substances and Disease Registry (ATSDR). 1999. ToxFAQ’s. http://www.atsdr.cdc.gov/toxfaqs/tfacts146.pdf. Accessed 11/29/12.

Barrie, H.J. and H.E. Harding. 1947. Argyro-siderosis of the lungs in silver finishers. Br J Ind Med. 4:225-32. (Secondary reference from Drake and Hazelwood, 2005).

Bleehen, S.S., D.J. Gould, C.I. Harrington, et al. 1981. Occupational argyria; light and electron microscopic studies and x-ray microanalysis. Br J Dermatol 104:19-26. (Secondary reference from ASTDR, 1990).

Cha, K., H.W. Hong, Y.G. Choi, M.J. Lee, J.H. Park, H.K. Chae, G. Ryu, and H. Myung. 2008. Comparison of acute responses of mice livers to short-term exposure to nano-sized or micro-sized silver particles. Biotechnol Lett 30:1893-1899.

Cong, Y., G.T. Banta, H. Selck, D. Berhanu, E. Valsami-Jones, and V.E. Forbes, 2011. Toxic effects and bioaccumulation of nano-, micron- and ionic-Ag in the polychaete, Nereis diversicolor. Aq Toxico. 105: 403-411.

Danscher, 1981. Full reference not found. (Secondary reference in EPA, 2009).

Dequidt, J., P. Vasseur, J. Gromez-Potentier. 1974. Experimental toxicological study of some silver derivatives. Bulletin de la Societe de Pharmacie de Lille 1:23-35 (French). (Secondary reference from ASTDR, 1990).

Drake, P.L. and K.J. Hazelwood. 2005. Exposure-Related Health Effects of Silver and Silver Compounds: A Review. Ann Occup Hyg, 49(7):575-585.

East, B.W., K. Boddy, E.D. Williams, et al. 1980. Silver retention, total body silver and tissue silver concentrations in argyria associated with exposure to an anti-smoking remedy containing silver acetate. Clin Exp Dermatol 5:305-311. (Secondary reference from ASTDR, 1990).

ECHA (European Chemicals Agency), 2012. Information on Chemicals: Registered chemicals. Search term: CAS #7440-22-4. At: http://echa.europa.eu/web/guest/information-on-chemicals/registered-substances, accessed November 2^nd^, 2015.

Erekson, G.L. 2004. In Vivo Mouse Micronucleus Assay. Covance Laboratories, Inc. (Vienna, Virginia). Study Number 6132-202, MRID 464533-02. http://www.epa.gov/pesticides/chem_search/cleared_reviews/csr_PC-072506_27-Jul-05_a.pdf. Accessed 12/3/12.

ESPI Metals. 2011. MSDS. http://www.espimetals.com/index.php/msds/274-silver. Accessed November 20, 2012.

Furchner, J.E., C.R. Richmond, G.A. Drake. 1968. Comparative metabolism of radionuclides in mammals-IV. Retention of silver-110m in the mouse, rat, monkey, and dog. Health Physics 15:505-514. (Secondary reference from ASTDR, 1990).

Gaiser, B.K., T.F. Fernandes, M.A. Jepson, J.R. Lead, S.R. Tyler, M. Baalousha, A. Biswas, G.J. Britton, P.A. Cole, B.D. Johnston, Y. Ju-Nam, P. Rosenkranz, T.M. Scown, and V. Stone. 2012. Interspecies comparisons on the uptake and toxicity of silver and cerium dioxide nanoparticles. Environ Toxicol and Chem 31(1):144-154.

Gaiser, B.K., A. Biswas, P. Rosenkranz, M.A. Jepson, J.R. Lead, V. Stone, C.R. Tyler, and T.F. Fernandes. 2011. Effects of silver and cerium dioxide micro- and nano-sized particles on Daphnia magna. J Environ Monit. May 13(5):1227-35.

Gaiser, B.K., T.F. Fernandes, M.A. Jepson, J.R. Lead, S.R. Tyler, and V. Stone. 2009. Assessing exposure, uptake and toxicity of silver and cerium dioxide nanoparticles from contaminated environments. Environ Health 8(Suppl 1):S2. Doi:10.1186/1476-069X-8-S1-S2.

Garneir, J., J.P. Baudin, and L. Foulquier. 1990. Accumulation from water and depuration of 110 mAg by a freshwater fish, Salmo trutta L. Wat Res. Vol 24(11): 1407-1414. (Secondary reference in ECHA).

German FEA. 2012. Umwelt Bundes Amt, Für Mensch und Umwelt. http://webrigoletto.uba.de/rigoletto/public/searchRequest.do;jsessionid=88A1AE1DEE0223CFE7DD76871FE35F67?event=request. Accessed 11/21/12.

Hirsch, M.P. 1997. Bioaccumulation of silver from laboratory-spiked sediments in the Oligochaete (Lumbriculus Variegatus). Env Toxicol Chem. 17(4): 605-609. (Secondary reference in ECHA).

Gorth, D.J., D.M. Rand, T.J. Webster. 2011. Silver nanoparticle toxicity in Drosophila: size does matter. International Journal of Nanomedicine 6: 343-350.

Hazardous Substances and New Organisms (HSNO) regulations, Chemical Classification and Information Database (CCID), (New Zealand) Environmental Protection Authority. http://www.epa.govt.nz/search-databases/pages/hsno-ccid.aspx. Accessed 11/30/12.

Hazardous Substances Data Bank (HSDB), U.S. National Library of Medicine. 2003. http://toxnet.nlm.nih.gov/cgi-bin/sis/htmlgen?HSDB. (Secondary reference in NITE, 2006, accessed 11/30/12).

Hinther, A., S. Vawda, R.C. Skirrow, N. Veldhoen, P. Collins, J.T. Cullen, G. Van Aggelen, and C.C. Helbing. Nanometals induce stress and alter thyroid hormone action in amphibian at or below North American water quality guidelines. Environ. Sci. Technol. 44:8314-8321.

Hogstrand, C., F. Galvez and C. Wood. 1996. Toxicity, silver accumulation and metallothionein induction in freshwater rainbow trout during exposure to different silver salts. Environ Toxicol Chem. 15:1102-1108. (Secondary reference from WHO, 2002).

Integrated Risk Information System (IRIS), U.S. Environmental Protection Agency. 2003. http://www.epa.gov/iris/subst/0099.htm. Accessed 11/30/12.

Integrated Risk Information System (IRIS). 1989. http://www.epa.gov/iris/subst/0099.htm. Accessed November 20, 2012.

International Uniform Chemical Information Database (IUCLID). 2000. Dataset for Substance ID 7440-22-4, Silver. http://esis.jrc.ec.europa.eu/doc/IUCLID/data_sheets/7440224.pdf. Accessed 11/30/12.

Kanematsu, N., H. Masako and T. Kada. 1980. Rec assay and mutagenicity studies on metal compounds. Mutat. Res. 77:109-116.

Kent, RD and Vikesland, PJ. 2011. Controlled Evaluation of Silver Nanoparticle Dissolution using Atomic Force Microscopy. Environ Sci Technol. Just Accepted Manuscript • DOI: 10.1021/es203475a • Publication Date (Web): 15 Dec 2011 Downloaded from http://pubs.acs.org on December 29, 2011.

Kim, J., S. Kim, and S. Lee, 2011. Differentiation of the toxicities of silver nanoparticles and silver ions to the Japanese medaka (Oryzias latipes) and the cladoceran Daphnia magna. Nanotoxicology, Vol. 5, No. 2 , Pages 208-214.

Lacoue-Labarthe, T., P. Bustamante, E. Hörlin, A. Luna-Acosta, A. Bado-Nilles, H. Thomas-Guyon. 2009. Phenoloxidase activation in the embryo of the common cuttlefish Sepia officinalis and response to the Ag and Cu exposure. Fish & Shellfish Immunology 27:516-521.

MacIntyre, D., A.L.C. McLay, B.W. East, et al. 1978. Silver poisoning associated with an antismoking lozenge. Br Med J 2:1749-1750. (Secondary reference from ASTDR, 1990).

Matuk, Y., M. Ghosh, C. McCulloch. 1981. Distribution of silver in the eyes and plasma proteins of the albino rat. Can J Opthalmol 16:145-150. (Secondary reference from ASTDR, 1990, abstract available at http://www.ncbi.nlm.nih.gov/pubmed/7296363).

McCoy, E.C. and H.S. Rosenkranz. 1978. Silver Sulfadiazine: Lack of Mutagenic Activity. Chemotherapy 24: 87-91.

McLaughlin, J., and J.C.J. Bonzongo, 2012. Effects of Natural Water Chemistry on Nanosilver Behavior and Toxicity to Ceriodaphnia Dubia and Pseudokirchneriella Subcapitata, Environ Toxicol Chem. 31 (1):168-175.

National Institute of Technology and Evaluation (NITE), (Japanese) Incorporated Administrative Agency.2006. http://www.safe.nite.go.jp/english/ghs_index.html#results. Accessed 11/30/12.

NSF International. 2012. Conventional Silver as an EPA Registered Pesticide. Phase 1: Scoping Project (unpublished).

Olcott, C.T. 1948. Experimental argyrosis. IV. Morphologic changes in the experimental animal. Am J Path 24:813-833. (Secondary reference from ASTDR, 1990).

Patty's Industrial Hygiene (Patty’s), 2001. Wiley-Interscience, 5th edition. (Secondary reference cited in NITE, 2006, volume and page numbers were not specified).

Perrone, S., E. Clonfero, G. Gori and L. Simonato. 1977. Observations of four cases of occupational argyrosis. Med Lav. 68: 178-86. (Secondary reference in Drake and Hazelwood, 2005).

Phalen, R.F., P.E. Morrow. 1973. Experimental inhalation of metallic silver. Health Phys 24:509-518. (Secondary reference from ASTDR, 1990).

Pifer, J.W., B.R. Friedlander, R.T. Kintz, et al. 1989. Absence of toxic effects in silver reclamation workers. Stand J Work Environ Health 15:210-221. (Secondary reference from ASTDR, 1990).

RIVM, 2009. Nanomaterials under REACH, Nanosilver as a case study. Report 601780003, National Institute for Public Health and the Environment.

Robison, S.H., O. Cantoni and M. Costa. 1982. Strand breakage and decreased molecular weight of DNA induced by specific metal compounds. Carcinogenesis. 3(6):657-662.

Rosenman, K.D., A. Moss, S. Kon. 1979. Argyria: Clinical implications of exposure to silver nitrate and silver oxide. J Occup Med 21:430-435. (Secondary reference from ASTDR, 1990).

Rosenman, K.D., N. Seixas, I. Jacobs. 1987. Potential nephrotoxic effects of exposure to silver. Br J Ind Med 44:267-272. (Secondary reference from ASTDR, 1990).

Rungby, J., L. Slomianka, G. Danscher, A.H. Andersen and M.J. West. 1987. A quantitative evaluation of the neurotoxic effect of silver on the volumes of the components of the developing rat hippocampus. Toxicol. 43(3): 261-8. (Secondary reference from EPA, 1993).

Rungby, J. and G. Danscher. 1984. Hypoactivity in silver exposed mice. Acta Pharmacol Toxicol 55:398-401. (Secondary reference from ASTDR, 1990).

Rungby, J. and G. Danscher. 1983. Localization of Exogenous silver in Brain and Spinal Cord of Silver Exposed Rats. Acta Neuropathol. 60: 92-98. (Secondary reference from EPA, 1993).

Scown, T.M., E.M. Santos, B.D. Johnston, B. Gaiser, M. Baalousha, S. Mitov, J.R. Lead, V. Stone, T.F. Fernandes, M. Jepson, R. van Aerle, and C.R. Tyler. 2010. Effects of aqueous exposure to silver nanoparticles of different sizes in Rainbow Trout. Toxicol Sci 115(2): 521-534.

Smith, I. and B. Carson. 1977. Trace metals in the environment. Volume 2. Silver. Ann Arbor, MI, Ann Arbor Science Publishers, 469. (Secondary reference in WHO, 2002).

U.S. Environmental Protection Agency (EPA). Office of Prevention, Pesticides and Toxic Substances. 2009. Memorandum: Silver, Silver salts, and Silver Zeolites: Human Health Assessment Scoping Document in Support of Registration Review. http://www.regulations.gov/#!documentDetail;D=EPA-HQ-OPP-2009-0334-0002. Accessed 12/6/12.

U.S. Environmental Protection Agency (EPA), Office of Prevention, Pesticides and Toxic Substances. 1993. Re-registration Eligibility Decision (RED) Facts, Silver. http://www.epa.gov/oppsrrd1/REDs/factsheets/4082fact.pdf. Accessed 12/6/12.

Walker, F. 1971. Experimental argyria: A model for basement membrane studies. Br J Exp Pathol 52:589-593. (Secondary reference from ASTDR, 1990).

Warnau, M., J.L. Teyssie and S.W. Fowler. 1996. Biokinetics of selected heavy metals and radionuclides in the common Mediterranean echinoid Paracentrotus lividus: sea water and food exposures. Marine Ecology Progress Series, 141: 83-94. (Secondary reference in ECHA, 2012).

Webb N. and C.M. Wood. 2000. Bioaccumulation and distribution of silver in four marine teleosts and two marine elasmobranchs: influence of exposure duration, concentration and salinity. Aquatic Toxicol 49: 111-129. (Secondary reference in ECHA, 2012).

Wood, C. C. Hogstrand, F. Galvez and R. Munger. 1996. The physiology of waterborn silver toxicity in freshwater rainbow trout (Oncorhynchus mykiss): 2. The effects of silver thiosulfate. Aquatic Toxicology. 35: 111-125. (Secondary reference in WHO, 2002).

Wood, C., S. Munger, F. Galvez and C. Hogstrand. 1994. The physiology of silver toxicity in freshwater fish. In: Andren A., Bober T., eds. Transport, fate, and effects of silver in the environment. Proceedings of the 2nd international conference. 11-14 September 1994. Madison, WI, University of Wisconsin Sea Grant Institute, 109-114. (Secondary reference in WHO, 2002).

World Health Organization (WHO). 2002. Concise International Chemical Assessment Document 44, Silver and Silver Compounds: Environmental Aspects. http://www.who.int/ipcs/publications/cicad/en/cicad44.pdf. Accessed 11/9/12.

# APPENDIX A: Hazard Benchmark Acronyms

**(alphabetical order)**

**(AA) Acute Aquatic Toxicity**

**(AT) Acute Mammalian Toxicity**

**(B) Bioaccumulation**

**(C) Carcinogenicity**

**(CA) Chronic Aquatic Toxicity**

**(Cr) Corrosion/ Irritation (Skin/ Eye)**

**(D) Developmental Toxicity**

**(E) Endocrine Activity**

**(F) Flammability**

**(IrE) Eye Irritation/Corrosivity**

**(IrS) Skin Irritation/Corrosivity**

**(M) Mutagenicity and Genotoxicity**

**(N) Neurotoxicity**

**(P) Persistence**

**(R) Reproductive Toxicity**

**(Rx) Reactivity**

**(SnS) Sensitization- Skin**

**(SnR) Sensitization- Respiratory**

**(ST) Systemic/Organ Toxicity**

**Appendix B**

**Optional Hazard Summary Table**

| **Route** | **GreenScreen™Hazard Ratings: Conventional (low-solubility, non-nanoscale) silver** | | | | | | | | | | | | | | | | | | | |
| --- | --- | --- | --- | --- | --- | --- | --- | --- | --- | --- | --- | --- | --- | --- | --- | --- | --- | --- | --- | --- |
|  | **Group I Human** | | | | | **Group II and II Human** | | | | | | | | | **Ecotox** | | **Fate** | | **Physical** | |
|  | **C** | **M** | **R** | **D** | **E** | **AT** | **ST** | | **N** | | **SnS** | **SnR** | **IrS** | **IrE** | **AA** | **CA** | **P** | **B** | **RX** | **F** |
|  |  |  |  |  |  |  | _Single_ | _Repeated_ | _Single_ | _Repeated_ |  |  |  |  |  |  |  |  |  |  |
| o | DG | *M* | DG | DG | DG | **L** | DG | DG | DG | DG | *L* | DG | **L** | **M** | **vH** | *vH* | **vH** | **L** | **L** | **L** |
| d | DG |  | DG | DG |  | **L** | DG | DG | DG | DG |  |  |  |  |  |  |  |  |  |  |
| i | DG |  | DG | DG |  | DG | DG | DG | DG | DG |  |  |  |  |  |  |  |  |  |  |

1. Use GreenScreen® Assessment Procedure (Guidance) V1.2 [↑](#footnote-ref-1)
2. See GreenScreen Guidance V1.2 [↑](#footnote-ref-2)
3. Note any composition or hazard attributes of the chemical product relevant to how it is manufactured. For example, certain synthetic pathways or processes result in typical contaminants, by-products or transformation products. Explain any differences between the manufactured chemical product and the GreenScreen assessment of the generic chemical by CAS #. [↑](#footnote-ref-3)
4. See Appendix A for a glossary of hazard endpoint acronyms [↑](#footnote-ref-4)
5. See Appendix B for alternative GreenScreen Hazard Summary Table (Classification presented by exposure route) [↑](#footnote-ref-5)
6. For inorganic chemicals only, see GreenScreen Guidance V1.2 Section 14.4. (Exceptions for Persistence) [↑](#footnote-ref-6)
7. For Systemic Toxicity and Neurotoxicity, repeated exposure data are preferred. Lack of single exposure data is not a Data Gap when repeated exposure data are available. In that case, lack of single exposure data may be represented as NA instead of DG. See GreenScreen Guidance V1.2 Section 9.3. [↑](#footnote-ref-7)
8. See GreenScreen Guidance V1.2 Section 13 [↑](#footnote-ref-8)
9. A moiety is a discrete chemical entity that is a constituent part or component of a substance. A moiety of concern is often the parent substance itself for organic compounds. For inorganic compounds, the moiety of concern is typically a dissociated component of the substance or a transformation product. [↑](#footnote-ref-9)
10. The CPA “Red List” refers to chemicals 1. flagged as Benchmark 1 using the GreenScreen™ List Translator or 2. flagged as Benchmark 1 or 2 using the GreenScreen™ List Translator and further assessed and assigned as Benchmark 1. The most recent version of the GreenScreen™ List Translator should be used. [↑](#footnote-ref-10)
11. The way you conduct assessments for transformation products depends on the Benchmark Score of the parent chemical (See Guidance). [↑](#footnote-ref-11)
