# Supplementary material for: Use of a modified GreenScreen tool to conduct a screening-level comparative hazard assessment of conventional silver and two forms of nanosilver
Source: Environ Health. 2016 Nov 8;15:105. doi: 10.1186/s12940-016-0188-y (PMC5101654; doi:10.1186/s12940-016-0188-y)
Supplement: Additional file 2: — GreenScreen for Nanosilver 2015. (DOCX 180 kb) [file 12940_2016_188_MOESM2_ESM.docx]

**Chemical Hazard Assessment for *Nanosilver, metallic (CAS # 7440-22-4)***

**Modified from GreenScreen^®^ Version 1.2^[[1]](#footnote-1)^**

| **Initial Modified GreenScreen^®^ Assessment Prepared By:** | **Initial Modified GreenScreen^®^ Assessment Quality Control Performed By:** |
| --- | --- |
| Name: Nancy Linde  Brad Lampet | Name: Teresa McGrath |
| Title: Toxicologist | Title: Senior Managing Toxicologist |
| Organization: NSF International | Organization: NSF International |
| Date: June 12th, 2012  Revised: December 18, 2013  Revised: June 30, 2014  Last literature search performed: September 12, 2013. | Date: June 12th, 2012  Revised: December 18, 2013  Revised: July 22nd, 2014 |
|  | **Updated by**: Eric Rosenblum, Ph.D., D.A.B.T  Organization: Rosenblum Environmental LLC  Date: October 31, 2015 |

**Confirm application of the *Disclosure and Assessment Rules and Best Practice*^[[2]](#footnote-2)^:** (List disclosure threshold and any deviations)

Because this review is based on generic nanosilver, not a particular manufacturer’s product, the de minimus rule is not applicable.

**Chemical Name (CAS #):** Silver, CAS # 7440-22-4, restricted to the nano forms with average particle size <100 nm, and includes nanosilver capped and stabilized (see properties below).

**Also Called:**

Silver, AgNP

**Suitable analogs or moieties of chemicals used in this assessment (CAS #’s):**

Silver Chloride (nanoscale) (CAS # 7783-90-6).

**Chemical Structure(s):**

Ag (see description of properties below)

AgCl (silver chloride, nanoscale)

**Notes related to production specific attributes^[[3]](#footnote-3)^:**

**For Inorganic Chemicals and relevant particulate organics (*if not relevant, list NA*)**

**Define Properties:**

The following inorganic chemical characteristics were examined in each study and reported where available as part of the assessment of study quality and relevance:

1. Particle size (e.g. silica of respirable size). Range 1-~100 nm; mean or median < ~ 100 nm.
2. Structure (e.g. amorphous vs. crystalline).
3. Mobility (e.g. Water solubility, volatility).
4. Bioavailability

The following additional nanoparticle characteristics (Card and Magnuson, 2010) were examined in each study and reported where available as part of the assessment of study quality and relevance:

1. agglomeration and/or aggregation
2. chemical composition
3. purity
4. shape – spherical unless stated otherwise
5. surface area
6. surface charge
7. surface chemistry (including composition and reactivity). Any of the following stabilizers or capping agents are in scope: PVP (polyvinylpyrrolidone), CMC (carboxymethylcellulose), citrate, carbonate, and starch. The following strong ligands/stabilizing agents are out of scope: cysteine, anything “mercapto-“ , or “thiol”, serum albumin, “passivated” nanoparticles (which means they are surface-derivatized and surface-functionalized with strong ligands); also surfactants (without controls for inherent toxicity); nanocomposites
8. whether any characterization was conducted in the relevant experimental media.

**Identify Applications/Functional Uses:**

**(e.g., Cleaning product, TV casing)**

1. Textile applications as an antimicrobial fabric protector

**GreenScreen Benchmark Score and Hazard Summary Table:^[[4]](#footnote-4),^^[[5]](#footnote-5),^^[[6]](#footnote-6),^^[[7]](#footnote-7)^ Nanosilver** was assigned a Benchmark Score of **1** based on criteria 1c. very high persistence coupled with high repeat dose systemic toxicity and very high ecotoxicity.

Note: Hazard levels (Very High (vH), High (H), Moderate (M), Low (L), Very Low (vL)) in *italics* reflect estimated values, authoritative B lists, screening lists, weak analogues, and lower confidence. Hazard levels in **BOLD** font are used with good quality data, authoritative A lists, or strong analogues. Group II Human Health endpoints differ from Group II* Human Health endpoints in that they have four hazard scores (i.e., vH, H, M and L) instead of three (i.e., H, M and L), and are based on single exposures instead of repeated exposures.

**Environmental Transformation Products and Ratings^[[8]](#footnote-8)^:**

**Identify feasible and relevant environmental transformation products** **(i.e., dissociation products, transformation products, valence states)** **and/or moieties of concern**^[[9]](#footnote-9)^

| **Life Cycle Stage** | **Transformation Pathway** | **Transformation Products** | **CAS #** | **On CPA Red List^^[[10]](#footnote-10)^^?** | **GreenScreen™Rating^^[[11]](#footnote-11)^^** |
| --- | --- | --- | --- | --- | --- |
| Textile Production (masterbatch, fiber and fabric preparation, fabric padding process) | Degradation or dissociation followed by agglomeration | Pure Ag^0^ aggregates | 7440-22-4 | Yes | 1 (See note below) |
| Consumer use (release from textiles during wear and washing) |  |  |  |  |  |
| End of life (release, dissolution, aggregation, precipitation) |  |  |  |  |  |
| Textile Production (masterbatch, fiber and fabric preparation, fabric padding process) | In the presence of hypochlorite, elemental silver may oxidize and convert into silver chloride (EPA-HQ-OPP-2009-1012-0015.pdf, EPA, 2010). | Silver chloride (AgCl) | 7783-90-6 | No |  |
| Consumer use (release from textiles during wear and washing) |  |  |  |  |  |
| End of life (release, dissolution, aggregation, precipitation) |  |  |  |  |  |
| Consumer use where release from textiles occurs during wear and washing | Release / degradation / dissociation | Silver ion (Ag+) | 14701-21-4 | No |  |
| End of life (release, dissolution, aggregation, precipitation) |  |  |  |  |  |
| End of life (release, dissolution, aggregation, precipitation) | Release / dissolution / aggregation / precipitation | Silver sulfide (Ag_2_S) | 21548-73-2 | No |  |
| End of life (release, dissolution, aggregation, precipitation) | Release / dissolution / aggregation / precipitation | Silver thiosulfate (Ag_2_H_2_O_3_S_2_) | 23149-52-2 | No |  |

Note: For the above noted transformation products, silver (CAS# 7440-22-4) was listed on the Japanese NITE list as GHS Category 1 for respiratory effects – Specific target organ/systemic toxicity following single exposure. This translates to *vH* concern for a Group II Human endpoint, and may result in a **BM 1** score when coupled with **vH** persistence. It may be further noted the NITE listing is based on the ACGIH (2001) reports pertaining to pulmonary edema developed after exposure to heated metallic silver fumes, and ATSDR (1997) reported irritation from occupational exposure to dust.

**Introduction:**

See “’Nanosilver’ as used in textiles Phase I: Scoping Project” (English, 2012). Please note that the data in this report are based on published literature as of September 12, 2013. Nanosilver is a very active area of research and new studies have since become available that are not included in this hazard summary.

**Hazard Classification Summary Section:**

**For all hazard endpoints:**

- **Search all GreenScreen specified lists. Report relevant results either in each hazard endpoint section or attach to the end of the report.**
- **Always indicate if suitable analogs or models were used.**
- **Attach modeling results (See Appendix C).**
- **Include all references either in each hazard endpoint section or at the end of the report.**

**Group I Human Health Effects (Group I Human)**

**Carcinogenicity (C) Score (H, M or L):** DG

Nanosilver was assigned a score of **Data Gap** for carcinogenicity based on lack of data.

**Mutagenicity/Genotoxicity (M) Score (H, M or L):** *M*

Nanosilver was assigned a score of **Moderate** (low confidence) for mutagenicity based on limited or marginal evidence of mutagenicity in both in vivo and in vitro mutagenicity/genotoxicity assays. The hazard score is reported in low confidence as expert judgement and weight of evidence was used in determining the score

Authoritative and Screening Lists

- - *Authoritative: Not on any authoritative lists*
  - *Screening: Not on any screening lists*

*In vivo -* mammalian

- An *in vivo* rat bone marrow micronucleus assay was conducted in Sprague-Dawley rats per OECD 474. Rats were orally exposed to nanosilver (average 60 nm, range 52.7-70.9 nm) at doses of 0, 30, 300 or 1000 mg/kg-bw/day by gavage in 0.5% aqueous carboxy methylcellulose for 28 days. The frequency of micronucleated polychromatic erythrocytes (MN PCEs) in 2000 PCEs for male rats was 5.20, 6.00, 6.60, and 7.40, for the controls, low, mid, and high dose groups respectively. For females, the frequency of MN PCEs was 2.50, 3.50, 2.40, and 3.40, respectively. Authors note while there was an apparent dose-related response in males for MN PCE’s, there was no statistically significant difference in the frequency of MN PCE’s or in the ratio of PCE/(PCE + normochromatic erythrocytes) for either sex. Authors concluded the nanosilver was not genotoxic or cytotoxic to the bone marrow under the test conditions (Kim et al., 2008).
- In an *in vivo* study reported as reliable with restrictions, silver nanoparticles with different sizes and coatings were evaluated using the mouse micronucleus assay. Five male B6C3F1 mice per group were treated with 5 nm polyvinylpyrrolidone (PVP)-coated silver nanoparticles at a single dose of 0.5, 1.0, 2.5, 5.0, 10.0 or 20.0 mg/kg bw via intravenous injection or with 15–100 nm PVP- or 10–80 nm silicon-coated silver nanoparticles at a single or 3-day repeated dose of 25.0 mg/kg bw. According to the authors, PVP-coated silver nanoparticles were more cytotoxic than the silica-coated silver nanoparticles in the bone marrow; but the silver nanoparticles did not induce genotoxicity. No DNA strand breaks were observed in the liver using the standard Comet assay, but PVP- and silicon-coated silver nanoparticles were found to cause DNA breaks in liver in the enzyme-modified Comet assay (Li 2013).
- Genotoxicity testing (OECD 474, *in vivo* micronuclei test) was conducted after exposing male and female Sprague-Dawley rats to silver nanoparticles by inhalation for 90 days according to OECD test guideline 413 (Subchronic Inhalation Toxicity: 90 Day Study) with a good laboratory practice system. The rats were exposed to silver nanoparticles (18 nm diameter) at concentrations of 0.7 × 106 particles/cm^3^ (low dose), 1.4 × 106 particles/cm^3^ (middle dose), and 2.9 × 106 particles/cm^3^ (high dose) for 6 hr/day in an inhalation chamber for 90 days. The rats were killed 24 hr after the last administration, then the femurs were removed and the bone marrow collected and evaluated for micronucleus induction. There were no statistically significant differences in the micronucleated polychromatic erythrocytes or in the ratio of polychromatic erythrocytes among the total erythrocytes after silver nanoparticle exposure when compared with the control. The authors conclude that results suggest that exposure to silver nanoparticles by inhalation for 90 days does not induce genetic toxicity in male and female rat bone marrow in vivo (Kim 2011c).
- In a poorly reported study the cytotoxicity and genotoxicity of silver nanoparticles at different doses and particle sizes to bone marrow cells was investigated. Rats were injected intravenously with a single dose of 5 or 10 mg/kg bw of 20 nm silver nanoparticles or with 5 mg/kg bw 200 nm silver nanoparticles. The samples were taken at 24 hours, 1 week and 4 weeks following the exposure. Micronucleus test was used to detect DNA damage. According to the authors, the nanoparticles induced significant levels of micronuclei in PCE of bone marrow. Increased level of micronuclei was observed not only one day after the exposure, but also in later time points. Results showed that the frequency of micronuclei decreased within 4 weeks. At 1 wk after the exposure the frequencies of micronuclei were similar in all silver nanoparticle exposed groups, whereas 4 weeks after exposure the enhanced level of micronuclei was observed only in case of the highest dose (10 mg/kg bw) of 20 nm silver nanoparticles. The results with the comet assay did not show significant increase of DNA damage in bone marrow leukocytes of rats. Such results were present at all time points post single treatment to silver nanoparticles. (Dobrzyńska 2014).
- Mouse bone marrow cells – male Swiss albino mice were exposed to nanosilver particles via a single i.p. injection at 10, 20, 40 or 80 mg/kg-bw. Silver nanoparticles characterization was performed using TEM (75-130 nm, average 125nm), SEM (90-180 nm, average 120 nm), EDX and XRD (confirmed no impurities), DLS (zeta potential of -4.86 mV suggested agglomeration), and UV-vis spectrophotometry (420-440 nm). Silver nanoparticles solutions (in PBS or water) were dispersed with sonication. Monitoring with UV visible spectra and dynamic light scattering indicated that the nanoparticles were agglomerating, with more agglomeration occurring at higher concentrations.
  - Comet assay – the % tail DNA was increased (p ≤ 0.05) for mice exposed at 10 and 20 mg/kg, but not at 40 and 80 mg/kg. The greatest increase compared to controls was ~5-fold at 20 mg/kg, whereas the positive control had a ~16-fold increase in % tail DNA.
  - Chromosome aberration assay – 16.5h after the administration of the test compound, animals were injected i.p. with 0.04% colchicine, then sacrificed 90 minutes later. Bone marrow samples were collected 18h after the silver nanoparticles exposure, when the cells were in their first metaphase. Aberrations in nanosilver exposed cells were mainly chromatid breaks (whereas the control demonstrated chromatid and chromosome aberrations). Results were significant (p ≤ 0.05) at all dose levels compared to control, but did not show a dose-response. According to the authors, silver nanoparticles can be classified as clastogen (Ghosh et al., 2012)

*In vitro* and/or non-mammalian

- An Ames test was performed using S. typhi TA98, TA100, TA1535, and TA1537, and E. coli WP2uvrA, with and without metabolic action with rat liver S-9. The test substance was AgNP (10 nm) suspended in 1% citric acid solution. Cytotoxicity was exhibited at concentrations of 125 µg/plate in TA98, TA100 and TA1537, and at 250 µg/plate in TA1535 and WP2uvrA in the presence of metabolic activation. Precipitation and aggregation were also noted at >1.25 µg/plate with and without activation. When compared to the negative control, there was no significant number of revertant colonies for any of the bacterial strains with or without activation, and there was no dose-dependent increase in revertant colonies (Kim et al., 2012). NSF does not consider bacterial reverse mutation assays to be reliable for assessing the mutagenic potential of a bactericidal agent such as nanosilver, which is toxic to bacteria at low doses. Moreover, bacterial mutation assays may be unsuitable for nanoparticulate substances in general because they may fail to penetrate the bacterial cell wall, and therefore this study was discounted from the evaluation.
- Two widely used in vitro cell culture models, human liver HepG2 cells and human colon Caco2 cells, and flow cytometry techniques were evaluated as tools for rapid screening of potential genotoxicity of food-related nanosilver. Comparative genotoxic potential of 20 nm silver was evaluated in HepG2 and Caco2 cell cultures by a flow cytometric-based in vitro micronucleus assay. The nanosilver, characterized by the dynamic light scattering, transmission electron microscopy and inductively coupled plasma-mass spectrometry analysis, showed no agglomeration of the silver nanoparticles. The inductively coupled plasma-mass spectrometry and transmission electron microscopy analysis demonstrated the uptake of 20 nm silver by both cell types. The 20 nm silver exposure of HepG2 cells increased the concentration-dependent micronucleus formation sevenfold at 10 µg ml(-1) concentration in attached cell conditions and 1.3-fold in cell suspension conditions compared to the vehicle controls. However, compared to the vehicle controls, the 20 nm silver exposure of Caco2 cells increased the micronucleus formation 1.2-fold at a concentration of 10 µg ml(-1) both in the attached cell conditions as well as in the cell suspension conditions (Sahu 2014).
- In an *in vitro* genetic toxicity study that contained significant methodological and reporting deficiencies, the comet assay (single cell gel electrophoresis; performed in alkaline conditions (pH > 13)) was used to study DNA strand breaks and alkaline labile sites in human bronchial epithelial BEAS 2B cells following silver nanoparticle (AgNP) exposure. Semiconfluent cultures on 24-well plates were exposed for 4 and 24 hours to seven doses of AgNPs: 2, 4, 8, 16, 24, 36 and 48 g/cm^2^ (corresponding to 7.6, 15.2, 30.4, 60.8, 91.2, 136.8, 182.4 g/ml). Untreated and positive controls were included in all series. Silver nanoparticles produced more than 2-fold increase in the percentage of DNA in comet tail (P < 0.0001) at 16 µg/cm² and higher doses, after the 4-hour and 24-hour treatments. The trend was significantly dose-dependent after both treatments (P < 0.0001, slope increase of 0.1% and 0.06% DNA in tail per µg/cm² after 4 hours and 24 hours, respectively). The positive control (20 mM H2O2) induced a clear increase in the percentage of DNA in tail (6.5-fold in the 4-hour and 5.3-fold in the 24-hour treatment) (Nymark 2013).
- In an in vitro mammalian cell micronucleus test the cytotoxic and genotoxic effects of silver nanoparticles on primary Syrian hamster embryo (SHE) cells were investigated. Cell viability was assessed using a methyl tetrazolium (MTT) and genotoxic potential was evaluated using a cytokinesis-block micronucleus (CBMN) assay. There was a significant increase (P<0.05) in the frequency of micronucleus for all cells exposed to silver-nanoparticles. The increase in micronucleation frequency was not found to be dose-dependent at 40 µg/mL exposre. According to the authors, these results suggested that silver-nanoparticles may induce chromosome damage in primary Syrian hamster embryo cells. It should be noted that authors did not account for cytotoxic effects during MN evaluation, thus difficult to assess whether positive findings were caused by direkt clastogenic effect interaction or via secondary cytotoxic mode of action (Li 2013).
- Genotoxicity of silver nanoparticles was evaluated in a bacterial reverse mutation assay and an *in vitro* micronucleus assay. AgNP size and distribution was measured with TEM (average particle sizes were 4-12 nm, with agglomeration sizes up to 30 nm), UV-visible spectrometry (max absorbance at 450 nm with a narrow peak width at half maximums suggesting a narrow distribution of sizes). AgNP test solutions were dispersed in sterilized water by vortexing for 5 minutes and then sonicating for 5 minutes (Li et al., 2012).
  - S. typhi strains TA102, TA100, TA1537, TA98, and TA1535 were treated with AgNPs at 10 different doses ranging from 0.15 to 76.8 μg/plate, per OECD 471. Toxicity limited the doses that could be assayed to 2.4-38.4 μg/plate. No increase in mutant frequency over the vehicle control was found for any concentration in any strain. The strains were exposed only in the absence of S9 because the authors suggested the test substance is unlikely to be metabolized by S9.
- Human lymphoblastoid TK6 cells were treated with 10-30 μg/mL AgNPs. Micronucleus frequencies were increased in a dose-dependent manner at 25 and 30 μg/mL, the results were positive with a 2.59-fold and 3.17-fold increase, respectively, compared to controls (a 3-fold increase is needed to call it “positive”). Significant cytotoxicity was observed at concentrations above 20 μg/mL, with 55% cytotoxicity at the highest dose of 30 μg/mL.^^[[12]](#footnote-12)^^ An *in vitro* chromosome aberration test was performed per OECD 473. Chinese hamster ovary cells were exposed to AgNP (10 nm) suspended in 1% citric acid solution. For the groups treated for 24 and 6 hours without the S9 mix, a cytotoxic effect was induced above 15.625 µg/mL, and for groups with S9, cytotoxicity was observed above 31.25 µg/mL. Doses were then set at 3.906, 7.813, and 15.625 µg/mL, with approximately 50% of the RCC as the highest concentration. In cells without S-9, there were no metaphase chromosomes observed at any dose levels after 24 h of continuous treatment, and also none after 6 h hours of treatment followed by 18 hours of recovery, therefore doses had to be set lower at 0.488, 0.977 and 1.953 µg/mL for 24 hour continuous treatment and 0.977, 1.953 and 3.906 µg/mL for the 6 hours of treatment. AgNPs did not produce any statistically significant increases in the number of cells with chromosome aberrations, or the number of cells with polyploidy or endoreduplication, when compared with the negative controls, with or without activation (Kim et al., 2012).
- *In vitro* analyses for cytotoxicity and genotoxicity of starch-coated silver nanoparticles (6-20 nm) was performed in normal human lung fibroblast cells (IMR-90) and in human glioblastoma (brain cancer) cells (U251).Toxicity endpoints included cell morphology, cell viability, metabolic activity and oxidative stress. Genotoxicity endpoints included DNA damage via the single cell gel electrophoresis (SCGE, aka Comet) assay and chromosome aberrations via the cytokinesis blocked micronucleus assay (CBMN).
  - The following cytotoxicity analyses were not performed to established guideline methods but are useful in characterizing mode of action:
    - Authors note changes in cell shape or morphology were observed but they do not specify if this was in the IMR-90 and/or U251 cells.
    - ATP assays demonstrated a concentration- and time-dependent drop in luminescence intensity in both cells types at 48 and 72 hours compared to the control (starch only).
    - Microscopic analysis showed no indication of massive cell death, but large numbers of floating cells combined with low ATP levels suggests the possibility of metabolic arrest. Therefore metabolic activity was measured using the cell titer blue assay and also led to a low ATP measure.
    - Reactive oxygen species (ROS) is a measure of oxidative stress. ROS was measured using the DCF-DA and DHE staining methods. In the presence of ROS, fluorescent intensity of stained cells will increase. Analysis showed a significant increase in hydrogen peroxide and superoxide production in cells treated with 25 and 50 μg/mL, but not further increase ≥100 μg/mL.
    - Analysis of cell cycle was performed to detect parameters such as apoptosis, cell cycle arrest, and evidence of DNA damage. Cells with damaged DNA will accumulate in gap1 (G_1_), DNA synthesis (S), or in gap2/mitosis (G_2_/M) phase. Flow cytometry on exposed cells showed both cell types had a concentration-dependent G_2_ arrest which was observed as an increase in cell population in G_2_/M phase compared to control. Exposure at 25 μg/mL marked the onset of G_2_/M arrest. As concentration increased to 400 μg/mL, there was a massive increase (~30%) in G_2_ population, compared to controls which had major cell populations in the G_1_ phase. No significant apoptosis was observed as indicated by the absence of cell population in subG_1_.
    - Annexin-V staining was performed to assess the extent and mode of cell death. Viable cells do not stain, necrotic cells show red fluorescence, apoptotic cells fluoresce green, and late apoptosis cells are dual stained. IMR-90 cells showed an increase of 5-9% in apoptotic cells from 25 to 100 μg/mL, relative to controls, which may be attributed to ROS production, and 16 ± 5% cell death due to late apoptosis and/or necrosis.
  - DNA damage was evaluated in a SCGE assay – AgNP treated cells showed a concentration-dependent increase in tail momentum compared to controls, in both cell types. DNA damage was dose dependent in the U251 cells, but did not increase in the IMR-90 cells beyond 100 μg/mL
  - A cytokinesis block micronucleus assay was performed using starch-coated silver nanoparticles (6-20 nm) in normal human lung fibroblast cells (IMR-90) and in human glioblastoma (brain cancer) cells (U251). Cells were treated with 0, 100, or 200 µg for 48 hours, followed by further incubation for 22 hours with cytochalasin B. Increases in micronuclei were significantly higher and showed a dose response at 100 and 200 μg/mL, when compared to controls (p < 0.05), and authors noted chromosome breaks were much high in cancer cells compared to fibroblasts (AshaRani et al., 2009).
- A Comet assay and analysis for bioaccumulation was performed in the polychaete*, Nereis diversicolor.* The worms were exposed to nominal concentrations of nanosilver (<100 nm, 99.5% metals basis, coated by 0.2 wt% PVP), micro-silver (2-3.5 μm, ≥99.9% trace metal analysis), and ionic silver (AgNO3-) at 0, 1, 5, 10, 25, and 50 µg/Ag/g dry weight sediment for 10 days. DNA damage, measured as tail moment and tail DNA intensities, was dependent on dose and silver-form. Damage was significantly higher at 25 and 50 µg/g dw in nano- and micro-Ag treatments, and at 50 µg/g dw for the ionic silver, compared to controls. The presence of highly crystalline material was observed in nanosilver, suggesting the presence of large silver particles (aggregates, 20-200 nm, average 162 nm). For macrosilver, 5-10% of non-crystalline material was observed, suggesting it was not as pure as described by the manufacturer (i.e. <99.9% purity), and had both micro and nano-sized particles (8nm – 3 um). Reported silver body burdens for the nano-, micron-, and ionic-silver treatments were 8.56, 6.92, and 9.86 µg/g dw, respectively. These values correspond to BAF factors of 0.17, 0.14, and 0.20, respectively (Cong et al., 2011). The Comet assay did not include controls for apoptosis, and the relevance of the measured DNA strand breaks to heritable genetic damage is uncertain.
- The genotoxicity of silver nanoparticles in *Drosophila melanogaster* was evaluated using the SMART assay, which is an *in vivo* test system based on the loss of heterozygosity in normal genes and the corresponding expression of two recessive markers (multiple wing hairs (mwh) and altered shape of wing hairs (flr3) in the wing blade of adult flies. Thus, the induced genotoxic effect is a significant increase in the frequency of mutant spots on the wings. The assay can detect somatic recombination and a diverse set of mutational events such as point mutations, deletions, and chromosomal aberrations. Silver nanoparticles were < 60 nm in size at a density of 1.2 g/mL. The particles were suspended in 10% ethylene glycol and dosages were prepared using distilled water without previous sonication. No differences in aggregation were observed between ethylene glycol and distilled water suspensions. Ethylene glycol and distilled water were used as negative controls, while ethyl methanesulfonate was used as a positive control. Silver nitrate was used to compare the effects of ionic vs. nanoparticulate forms. The majority of nanoparticles were spherical in shape and no agglomeration was observed dispersed in ethylene glycol or in water. The average hydrodynamic diameter in suspension was 45.39 nm, and zeta potential was -37.6 mV. Virgin females heterozygous for the flr3 gene were mated to males heterozygous for the mwh gene. Eggs from this cross were collected during 8-hour periods in culture bottles containing standard medium. The resulting 3-day old larvae were then transferred to plastic vials containing *Drosophila* medium prepared with 0.1, 1, 5, or 10 mM concentrations of AgNPs. The larvae were fed on this medium until pupation, and the wings of surviving adults were scored for the presence of spots resulting from point mutation, recombination, or small deletion of the wild type allele in the case of mwh, or point mutation or small deletion of the wild type allele in the case of flr3.

The results obtained with transheterozygous larvae (mwh/+ and flr3/+) indicate that AgNPs induced significant increases in the frequency of total mutant spots, mainly inducing small single mwh spots, compared to the negative control. It was apparent based on the small size of the mutant spots that progression of nanosilver particles to reach cell targets (wing imaginal disks) takes time and only produces genetic damage in the last stages of development. The negative and positive control observations were consistent with expectations. The experiment was repeated with balanced heterozygous flies, meaning that the chromosomes containing the mutant alleles of mwh were paired with a “balancer” chromosome that suppresses genetic recombination. In this experiment, the observed frequency of mutations in AgNP exposed larvae was not significantly different from the negative controls, suggesting that the genotoxic effects are mainly due to somatic recombination, and not the result of the nanosilver, but rather natural variation that occurs at a low frequency in drosophila somatic cells. Mutation frequencies resulting from exposure to silver nitrate was not significantly different from controls, suggesting a different genotoxic profile of the ionic form vs. the nanoparticulate form (Demir et al., 2011).

- Nanosilver powder (<100nm, 99.5%) was used to investigate the cytotoxicity and genotoxicity in mammalian cells lines via the dye exclusion assay (cytotoxicity), Comet assay (DNA damage), and mouse lymphoma thymidine kinase gene mutation assay (Kim et al., 2010a).
  - Cytotoxicity values were first determined using the trypan blue exclusion assay in the L5178Y mouse lymphoma cells, based on the IC20 at 3.8 mg/mL and 1.8 mg/mL, with and without S-9, respectively. Then IC20 values in the human bronchial epithelial (BEAS-2B) cells were 1.1 mg/mL and 0.8 mg/mL with and without S-9, respectively.
  - The Comet assay was performed in L5178Y mouse lymphoma cells at concentration of 0.9 mg/mL to 3.8 mg/mL with S-9, and 0.4-1.8 mg/mL without S-9, and in BEAS-2B cells at concentrations of 0.3 mg/mL to 1.2 mg/mL with activation, and 0.2-0.8 mg/mL without activation. All concentrations in both cell lines, with and without S-9, had significant increases in DNA damage compared to controls. As noted above, the relevance of the measured DNA strand breaks to heritable genetic damage is uncertain.
  - The mouse lymphoma assay was performed in both cells lines. Cytotoxicity was dose dependent with metabolic activation (measured as decrease in %RS (relative survival) and RTG (relative total growth). Authors reported Nanosilver exposure did not lead to a significant increase in mutation frequencies at all tested concentrations.
- Human lung cells were tested for genotoxicity stimulated by oxidative stress *in vitro*. Human normal bronchial epithelial (BEAS-2B) cells were exposed to nanosilver for analysis in the comet assay, two micronucleus assays (with and without cytochalasin B), and an oxidative stress fluorescence assay. Nanosilver powder (<100 nm) was dispersed in bronchial-epithelial growth medium and sonicated for 30 minutes, the filtered (pore size 200 nm). Size and shape of the nanosilver solutions was characterized by SEM, TEM, and DLS. The average particle size after filtration was 58.9 nm, and the distribution was 43-260 [although not stated, Figure 1 in Kim et al. 2011a shows a clear majority of aggregates were <100 nm]. Results suggest in vitro genotoxicity of silver nano-particles is related to ROS generation (Kim et al., 2011a).
  - Comet assay: cells were exposed to nanosilver alone, or nanosilver with one of 4 scavengers (mannitol (MAN), catalase (CAT), sodium selenite (SS), or superoxide dismutase (SOD)). BEAS-2B cells exposed at 0.1-10 μg/mL showed a dose-dependent increase in DNA breakage compared to controls with statistically significant increases at each dose level. Cells exposed to nanosilver and a scavenger also had increased olive tail moment (% DNA in the tail x distance between centers of mass), results were significantly ameliorated relative to cells exposed to nanosilver without a scavenger.
  - *In vitro* micronucleus assays: cells were exposed to nanosilver alone, or nanosilver with scavengers, and with or without cytochalasin B. Increases in micronucleus formation was statistically significant for all doses, with a dose-response, when exposed to nanosilver at 0.1-10 μg/mL with and without cytochalsin B, however results with cytochalasin B did not meet the criteria of 3-fold increase and therefore did not qualify as a positive response for clastogenicity. Cells exposed to nanosilver in the presence of scavengers also had increased rates of MN formation, but increases were significantly ameliorated.
  - Oxidative stress assay: cellular oxidative stress response was determined by monitoring reactive oxygen species (ROS) fluorescence which occurs when H_2_DCF-DA (2’,7’-dichlorodihydrofluorescein-diacetate) is oxidized to DCF (2’,7’-dichlorofluorescein) by ROS. DCF fluorescence intensity was significantly increased by exposure to nanosilver at 0.1-10 μg/mL after 24 h, indicating significant ROS generation.
- Cytotoxicity and genotoxicity of silver nanoparticles (<100 nm) was tested in human lymphocytes. Cytotoxicity was assessed using the trypan blue dye exclusion assay (measures plasma membrane integrity associated with necrosis), and MTT (3-(4,5-dimethylthiazolyl-2)-2,5-diphenyltetrazolium bromide) and WST (water soluble tetrazolium salt) assay (measures effects on mitochondrial dehydrogenase activity). Genotoxicity was assessed via the comet assay. Generation of ROS (reactive oxygen species) was assessed using the DCFDA (2’,7’ –dichlorofluorescein diacetate) assay. In addition, flow cytometry was performed to measure the mode of cell death and uptake of silver nanoparticles in the lymphocytes. A chromosome aberration assay and comet assay were also performed in mouse bone marrow cells [summarized above in the *in vivo* section], and in root or leaf tissues of *Allium* and *Nicotiana* plants. Silver nanoparticles characterization was performed using TEM (75-130 nm, average 125nm), SEM (90-180 nm, average 120 nm), EDX and XRD (confirmed no impurities), DLS (zeta potential of -4.86 mV suggested agglomeration), and UV-vis spectrophotometry (420-440 nm, ). Silver nanoparticles solutions (in PBS or water) were dispersed with sonication. Monitoring with UV visible spectra and dynamic light scattering indicated that the nanoparticles were agglomerating, with more agglomeration occurring at higher concentrations. (Ghosh et al., 2012):
  - Human lymphocytes – cells were exposed to nanosilver at 0, 0.025, 0.050, 0.1, 0.15 or 0.2 mg/mL :
    - Comet assay – tail intensity (% tail DNA) was higher than control values at all concentrations, but was statistically significant (p ≤ 0.05) only at 0.025, 0.05, and 0.2 mg/mL.
    - ROS generation – nanosilver exposed cells showed ~3-5 fold increase in fluorescence intensity (DCFDA) compared to controls.
    - Flow cytometry – the Trypan blue dye exclusion method revealed a dose-dependent decrease in viability of cells, significant at 0.15 mg/mL (72.54%) and above. Measures of mitochondrial dehydrogenase in the MTT and WST assays suggested a dose-dependent decrease in cell viability, significant at 0.025 and 0.05 mg/mL, respectively. Analysis of fluorescent-stained cells showed a small percentage of cells undergoing apoptosis, about 1.5-fold increase compared to controls, and a significant increase in the number of necrotic cells at 0.025 mg/mL and above.
    - Qualitative analysis of human blood cells exposed to nanosilver showed different degrees of deformity, damaged cell membranes, and vacuolation, compared to controls.
    - ROS generation – fluorescence intensity was ~1.5 and 1.4-fold greater at 10 and 20 mg/kg-bw, respectively, and was negligible at higher doses compared to controls.
  - Plant root and leaf tissues were exposed to nanosilver solutions at 0, 0.025, 0.05 or 0.075 mg/mL.
    - Comet assay – in Allium cepat, DNA strand breaks were increased in the roots and in shoots at 0.025 and 0.05 mg/mL but not at 0.075 mg/mL (p ≤ 0.05). In Nicotiana tabacum, DNA strand breaks increased in roots at 0.05 and 0.075 mg/mL, and in shoots/leaf at 0.025, 0.05 and 0.075 mg/mL, but without a dose-response.
    - Qualitative analysis of A. cepa root cells exposed to nanosilver revealed extensive vacuolation, loss of nuclear organization, ruptured plasma membrane, shrinkage of the protoplast, and localization of nanoparticles in the vacuoles.
- Mouse lymphoma L5178y/*Tk*^+/-^-3.7.2C cells were tested for silver nanoparticle (5nm)-induced mutations in the mouse lymphoma assay (MLA) (using the microwell and soft-agar versions). Additional analyses for loss-of-heterozygosity (LOH), a Comet Assay, and analysis of gene expression in oxidative stress-related genes were performed to examine potential mode of action. Uncoated AgNPs were suspended in sterilized water and sonicated to remove large aggregates. NP sizes were confirmed with TEM and DLS. In a preliminary range finding study, doses lower than 3 µg/mL were associated with little cytotoxicity whereas doses higher than 6 µg/mL with a 4 hr incubation resulted in less than 10% of relative total growth.
  - Mouse lymphoma cells were cultured in F5p medium containing 5 µg/mL of the AgNPs for 4 hr and then washed three times with PBS to remove non-incorporated particles from the cell membrane. Following a 2-day expression period, treated cultures were divided for analysis of mutations in both the microwell and soft-agar versions of the MLA. Ag-NPs (5nm) induced dose-dependent cytotoxicity and mutagenicity in mouse lymphoma cells with both methods. The mean mutation frequency for treatment with 5 µg/mL AnNPs was ~7X higher than in controls.
  - Genomic DNA was extracted from mutant clones. Polymerase chain reaction analysis of LOH at the *Tk* and other loci was performed to determine LOH or retention of heterozygosity at the given locus. The major type of mutation was LOH involving < 34 Mpb chromosomal alterations in chromosome 11, suggesting a clastogenic mode of action, possibly via oxidative stress.
  - The Comet assay was performed using the same treatment procedure as the MLA (above). In the standard Comet assay, there was no significant induction of DNA damage although the percentage of DNA in the tail increased slightly with increasing dose. As the sensitivity and specificity of this assay can be improved by incubating the lysed cells with lesion-specific endonucleases that recognize particular oxidized bases and create additional breaks, three enzymes were employed (FPG, EndoIII, and hOOG1). Addition of these lesion-specific endonucleases resulted in significant induction of DNA breaks in a dose-dependent manner (Mei et al., 2012).
  - Gene expression analysis of oxidative stress-related genes was performed using a pathway-specific polymerase chain reaction (PCR) array. The Mouse Oxidative Stress and Antioxidant Defense RT2 Profile PCR Array can measure the expression of 84 genes related to oxidative stress. Six vehicle control and six Ag-NP (5 µg/mL) treated cell samples were used for this study. Among the 84 genes, 59 were actively expressed in the mouse lymphoma cells. 17 Genes demonstrated a 1.5-fold change in the gene expression compared to untreated controls with p < 0.05, 9 of which were up-regulated, and 8 down-regulated. The functions of these differentially expressed genes are involved in the production of reactive oxygen species (ROS), antioxidants, oxidative stress response, oxygen transporters, and DNA repair.
- Medaka (*Oryzias latipes*) OLHNI2 (fin) cells were treated with 0.05 – 5 μg/cm^2^ of 30 nm silver nanoparticles for 24 hours. Solutions were prepared using silver nitrate as a reducing agent, and sodium citrate as a stabilizing agent. The authors hypothesized that concentration related aggregation of silver colloids occurred based on the measured size distribution (i.e. there was a significant peak ~200 nm). The silver nanoparticles were capped by sodium citrate, leaving a zeta-potential (surface charge) of -20 mV. This surface charge inhibited the aggregation potential. The particles are spherical in shape based on transmission electron microscopy. Silver nanoparticles were cytotoxic to medaka cells in a concentration-dependent manner. The LC50 for cytotoxicity was 0.33 μg/cm^2^. Silver nanoparticles induced a concentration-dependent chromosomal aberrations in OLHNI2 cells. Treatments of 0, 0.05, 0.1 and 0.3 μg/cm^2^ induced 8, 10.8, 16 and 15.8% metaphases with damage and 10.8, 15.6, 24 and 24 total aberrations in 100 metaphases, respectively. Metaphase cells could not be obtained at a concentration of 0.5 μg/cm^2^, indicating cell cycle arrest was occurring. The spectrum of damage included chromatid lesions, isochromatid lesions, chromatid exchanges and centromere spreading (Wise et al., 2010).
- DNA damage was examined in mouse embryonic stem (mES) cells and mouse embryonic fibroblasts (MEF) using uncoated silver nanoparticles (25nm) and polysaccharide coated nanosilver (25 nm). DNA damage was assessed by increases in p53 protein content, Rad51 and H2AX protein expression. P53 in the absence of stress is kept in low concentrations within the cell by rapid degradation via the ubiquitin pathway. However, in the presence of stress, such as DNA damage, p53 accumulates and triggers cell cycle arrest to provide time for the damage to be repaired, or if the damage is too extensive it will trigger self-mediated apoptosis. Rad51 is a biomarker for DNA double strain breakage repair. The AgNPs were suspended in deionized water and added to each type of cell at 50 µg/mL, the cell cultures were placed in an incubator at 37°C and 10% CO_2_ and harvested at 4, 24, 48 and 72 h after treatment for immediate analysis , or placed in -20°C for storage. The p53 was upregulated, Rad51 expression was increased, and H2AX expression was increased after treatment with both coated and uncoated AgNPs in both cell lines at 4 and 24 h after treatment. MTT analysis revealed that coated AgNPs exerted a more severe response in both kinds of mammalian cells than the uncoated AgNPs (Ahamed et al., 2008). NSF notes the methods used provide indirect measures of DNA damage and are of uncertain relevance to heritable genetic damage. .
- NT2 human testicular embryonic carcinoma cell line, primary testicular cells from C57BL6 mice (wild type), and 8-oxoguanine DNA glycosylase KO genotype were exposed to AgNPs of 20 nm and 200 nm in size to evaluate apoptosis, necrosis, and DNA strand breaks. 8-oxoguanine DNA glycosylase KO mice are defective in the repair of oxidated purine bases, which mimics the repair status of human testicular cells with oxidative damage. Cells were exposed to concentrations of 10, 50, or 100 µg/mL and incubated for a period of four hours. After incubation, apoptosis was assessed by PI/Hoescht staining, DNA damage (strand breaks) was assessed by the Comet assay, and release of IL-8/MIP-2, IL-6, and TNF-alpha was assessed by ELISA. Cellular metabolic activity was assessed by measuring the rate of absorption of MTT in PBS. All three cell lines showed a dose-dependent decrease in metabolic activity compared to controls with increasing concentrations of AgNP 200 nm and 20 nm. The reduction at the high dose was approximately 50%, with the 20 nm AgNPs having a slightly larger effect. AgNPs also decrease the number of viable cells, and increased the rate of apoptosis and necrosis in the NT2 cell line. The number of viable cells was reduced by approximately 30% at the high dose compared to controls for both 20 nm and 200 nm AgNPs. For the analysis of DNA strand breaks, the results showed low levels of AgNP-induced DNA strand breaks in NT2 cells or the primary testicular cells, although a positive concentration-dependent trend was observed in NT2 cells in the Comet assay without formamidopyrimidine-DNA glycosylase (Fpg), a bacterial enzymatic extract. Ag200 seemed to cause the highest level of damage, with about 25% DNA intensity in tail at the high dose, whereas Ag20 and TiO2-NP caused approximately 15% and 10% strand breakage, respectively, at the same concentration in NT2 cells. In contrast, the AgNPs seemed to cause little to no DNA-strand breaks in testicular cells derived from either WT or KO mice. Additionally, no significant increase in oxidative DNA damage was observed in any of these experiments. Therefore, AgNPs showed a tendency to cause DNA damage, although the effect was weak and not always statistically significant (Asare et al., 2012).

**Reproductive Toxicity (R) Score (H, M, or L):** *L*

Nanosilver was assigned a score of **Low** (low confidence) for reproductive toxicity based the absence of reproductive toxicity observed in a guideline oral exposure study at doses up to 250 mg/kg bw/d. Low confidence was assigned due to the fact that the study was a screening test. No data was available for either dermal or oral exposures.

- Authoritative and Screening Lists
  - *Authoritative: Not on any authoritative lists*
  - *Screening: Not on any screening lists*
- A combined repeated-dose toxicity study and reproductive/developmental toxicity screening test were performed in rats orally exposed to nanosilver particles, in accordance with OECD TG 422 and per GLP. Sprague Dawley rats were exposed citrate-capped AgNPs (7.9 ± 0.95 nm) in deionized water (prepared daily) at 0, 62.5, 125, or 250 mg/kg bw/d. Males were exposed from 14 days before mating, 14 days during mating, and 14 days post-mating. Females were exposed from 2 weeks before mating, during mating and gestation, and through 4 days of lactation, for a total of 52 days. All rats were maintained untreated during 14 days recovery for males, and 16 days for females. TEM analysis confirmed AgNP sizes at 8.8 ± 5.2 nm when measured immediately after preparation, and 7.7 ± 4.8 nm 1 day after preparation, close to the initial size. There were no deaths in any treatment group or controls.
  - General findings: there were no statistically significant differences in weight gain or food consumption for any groups, although males were noted as having greater food consumption and weight gain in all groups, compared to females. Alopecia was observed in one male at 125 mg/kg bw/d, and in two males at 250 mg/kg bw/d, at 37 and 38 days, respectively. In females, alopecia was noted in two pregnant rats in the vehicle-treated group, one at 62.5 mg/kg bw/d, two at 125 mg/kg bw/d, and in six at 250 mg/kg bw/d. For all rats with alopecia, only two females at 250 mg/kg bw/d recovered. One female at 250 mg/kg bw/d displayed a single incidence of salivation immediately after administration of the AgNP on day 1 after gestation. There were no statistically significant differences in estrus cycle between female groups. There were no statistically significant differences in hematology findings for any of the groups in either sex. The only statistically significant findings in biochemistry was males from the recovery group had decreased inorganic phosphate levels and increased levels of chloride ions, and females at 250 mg/kg bw/d had decreased aspartate aminotransferase levels. Authors noted some differences [unspecified] were observed in some urinalysis endpoints, but they were not significant relative to historical controls for this laboratory. Males at 250 mg/kg bw/d in the recovery group had significantly increased absolute and relative liver weight compared to controls (p < 0.05). Females in 250 mg/kg bw/d recovery group had significant increased absolute but not relative kidney weights (left and right) and adrenal gland weights (left only). At necropsy, yellow discoloration of the lung was observed in one male at 250 mg/kg, and multifocal white discoloration of the spleen and splenomegaly were observed in one female at 125 mg/kg bw/d. Granulomas were observed in 2 females at 250 mg/kg bw/d and in 1 recovery female at 250 mg/kg bw/d, the biological significance of this finding is not known [and the statistical significance was not reported]. Tissue levels of silver in high dose females were ~33 times higher in the liver, ~13 times higher in the kidney, and ~213 times in the lung, compared to controls. A NOAEL for repeated dose effects was not reported, as the significance of the observed alopecia and lung granulomas is not known.
  - Reproductive / developmental findings: there were no statistically significant differences in mating, fertility or pregnancy rate among groups, except for one non-pregnant rat in the control group, and one premature birth in the control group. There were no treatment related changes in functional observations, including auditory response, papillary reflex, acute pain sensitivity, motor activity and passive avoidance in any group. There were no statistically significant differences in gestation period, number of corpora lutea and implantation, delivery rate, number of live or dead pups, percentage of live and dead pups relative to number of implantations, pre- or post-implantation loss, sex ratio, survival rate, number of neonates with external abnormalities, or pup body weights on postnatal days 0 and 4 (Hong et al., 2013). A NOAEL for reproductive and developmental effects was not reported but may be inferred at 250 mg/kg bw/d. Meets GHS not classified = GreenScreen Low.

**Developmental Toxicity incl. Developmental Neurotoxicity (D) Score (H, M or L):** *L*

Nanosilver was assigned a score of **Low** (low confidence) for developmental toxicity based on the absence of developmental toxicity observed in an OECD 422 test combined repeated dose toxicity study with the reproductive/developmental screening showed no concern in rats at doses from 62.5 to 250 mg/kg bw/d (Hong et al., 2013). Low confidence was assigned due to the fact that the study was a screening test. No data was available for either dermal or oral exposures.

- Authoritative and Screening Lists
  - *Authoritative:*
    - *MAK - Pregnancy Risk Group D*
  - *Screening: Not on any screening lists*
- See above summary for a combined repeated-dose toxicity study and reproductive/developmental toxicity screening test performed in rats orally exposed to nanosilver particles, in accordance with OECD TG 422 and per GLP (Hong et al., 2013). A NOAEL for reproductive and developmental effects was not reported but may be inferred at 250 mg/kg bw/d. Meets GHS not classified = GreenScreen Low.
- Mahabady (2012) investigated teratogenicity of nanosilver (particle size not reported) in rat foetuses following a single intraperitoneal administration of 0.4 or 0.8 mg nanosilver/kg bw into pregnant rats on gestation days (GD) 8 or 9. The foetuses collected on gestation day 20 (of rats treated on GD 8 with both doses and from the highest dose group on GD 9) had significantly reduced weights and lengths. Lower placenta weights, volumes and widths compared to the control group were reported for all treated groups. There were no macroscopic anomalies or skeletal effects in rat foetuses. Presence or absence of maternal effects was not reported. The toxicological significance of reduced weights and sizes is unclear. In addition, intraperitoneal exposures are not typically used in GreenScreen hazard classifications. Therefore, this study has not been included in the hazard score due.

**Endocrine Activity (E) Score (H, M or L):** DG

Nanosilver was assigned a score of **Data Gap** for endocrine activity. It may be noted a single entry for nanosilver appears in TEDX, however it is for a water soluble form of nanosilver (proprietary carboxyl-functionalized coating) that is outside the scope of textile applications and is therefore not applicable to this GreenScreen.

- Authoritative and Screening Lists
  - *Authoritative: Not on any authoritative lists*
  - *Screening:*
    - *TEDX - Potential Endocrine Disruptors - Potential Endocrine Disruptor*
- Frog tissue derived from *Rana catesbeiana* tadpoles was exposed to water soluble nanosilver (2-10 nm diameter with a proprietary carboxyl-functionalized coating) at 0.06 µg/L – 5.5 mg/L in a cultured tail fin biopsy (C-fin) assay. The C-fin assay maintains tissue complexity and biological replication while multiple chemical responses can be assessed from the same individual. The affects on gene expression in the presence or absence of 3,3’,5’-triiodothyronine (T_3_) was examined using quantitative real-time polymerase chain reaction. Nanosilver perturbed T_3_-mediated signaling at concentrations that did not induce cell stress, however, positive controls were not included. (abstract only, cited in TEDX, Hinther et al., 2010).

**Group II and II* Human Health Effects (Group II and II* Human)**

*Note: Group II and Group II* endpoints are distinguished in the v 1.2 Benchmark system (the asterisk indicates repeated exposure). For Systemic Toxicity and Neurotoxicity, Group II and II* are considered sub-endpoints. When classifying hazard for Systemic Toxicity/Organ Effects and Neurotoxicity endpoints, repeated exposure results are required and preferred. Lacking repeated exposure results in a data gap. Lacking single exposure data does not result in a data gap when repeated exposure data are present (shade out the cell in the hazard table and make a note). If data are available for both single and repeated exposures, then the more conservative value is used.*

**Acute Mammalian Toxicity (AT) Group II Score (vH, H, M or L): L**

Nanosilver was assigned a score of **Low** (high confidence) for acute mammalian toxicity based on data for oral and dermal exposures. Insufficient data was available for acute inhalation toxicity despite the fact that a robust inhalation study does exist showing low concern at the highest dose tested, this dose was not sufficiently high to assign a concern level. The low score is based on test data and therefore is reported as high confidence.

- Authoritative and Screening Lists
  - *Authoritative: Not on any authoritative lists*
  - *Screening: Not on any screening lists*

Oral

- Sprague Dawley rat oral LD50 using AgNP (10 nm) suspended in 1% citric acid solution was >2000 mg/kg-bw/d when tested per OECD 423 (Kim et al., 2012). Does not require classification per GHS criteria. Low concern for acute oral toxicity per GreenScreen™.
- ICR mice were exposed to a single limit dose of 5,000 mg/kg AgNP solution in distilled water, per OECD 425. Purity was measured at 99.96%, particle size 10-20 nm by TEM, and UV-vis spectrometry showed a narrow distribution suggesting low agglomeration. The mouse oral LD50 > 5,000 mg/kg (Maneewattanapinyo et al., 2011).

Dermal

- Sprague Dawley rat dermal LD50 using AgNP (10 nm) suspended in 1% citric acid solution was >2000 mg/kg-bw/d when tested per OECD 402 (Kim et el., 2012). Does not require classification per GHS criteria. Low concern for acute dermal toxicity per GreenScreen™.
- Groups of 6 male Hartley albino guinea pigs were exposed to aqueous solutions of colloidal nanosilver (<100 nm as confirmed by TEM) at 0, 100, or 1,000 μg/mL (0, 0.1, or 1 mg/L) on 10% of the body surface area per OECD 402. No animals died (Korani et al., 2011). Due to lack of further details in the reporting (i.e. total applied dose), a dermal LD50 cannot be inferred. Informational only.

Inhalation

- Sprague Dawley rat 4 hr LC50 for nanosilver (18-20nm) was reported at > 750 µg/m^3^ (3.1E6 particles/cm^3^) in a whole-body inhalation exposure test performed per OECD 403, and to GLP (Sung et al., 2011). It may be noted there were no significant changes in body weight, clinical observations, or lung function tests relative to control animals. This equates to a 4 hr LC50 > 0.000750 mg/L. Authors noted exposure at higher concentrations could be achievable, however higher concentrations would be expected to generate a lot of sub-micro-sized agglomerated/aggregated particles. Furthermore, the high dose was 7.5 times greater than the current occupational exposure limit of silver dust by either the American Conference of Governmental Industrial Hygienists (ACGIH) or the Occupational Safety and Health Administration (OSHA). In terms of particle surface area, the high dose was more than 600 times the particle surface area of respiratory dust with a 4µm diameter. The reported LC50 does not correspond to a GreenScreen™ hazard rating. As higher concentrations were in fact not tested, it is not known if deaths may occur in the range of maximum saturation without agglomeration, therefore a data gap is assigned for acute inhalation toxicity.

**Systemic Toxicity/Organ Effects incl. Immunotoxicity (ST)**

**(ST-single) Group II Score (single dose: vH, H, M or L);** DG

Nanosilver was assigned a score of **Data Gap** for systemic toxicity/organ effects based on single exposure based on lack of data for all three routes of exposure (note the GreenScreen™ allows for no data with single exposure when there is data for repeat exposure – see below). The inclusion of silver under Japan GHS is based

- Authoritative and Screening Lists
  - *Authoritative: Not on any authoritative lists*
  - *Screening:*
    - *Japan - GHS - Specific target organs/systemic toxicity following single exposure - Category 1*
- The Japanese NITE classification for specific target organ / systemic toxicity following single exposure is “Category 1, danger, cause damage to organs (respiratory)” based on reports that lung disorders with pulmonary edema developed after exposure to heated metallic silver fumes for 4 hours as reported by ACGIH (2001), and that irritation to the airways develops after occupational exposure to the dust as reported by ATSDR Tox FAQs (1997) (NITE, 2006).
- ACGIH (2001) reported case studies of respiratory effects to silver fumes, however, silver fumes was determined to be outside the scope of this GreenScreen. ATSDR FAQ’s for silver discuss hazards associated with inhalation exposure in only a single statement: “Exposure to high levels of silver in the air has resulted in breathing problems, lung and throat irritation, and stomach pains” (ATSDR, 1999).

**(ST-repeat) Group II* Score (repeated dose: H, M, L): H**

Nanosilver was assigned a score of **High** concern (high confidence) for systemic toxicity/organ effects based on repeated inhalation exposure (Ji et al., 2007; Sung et al., 2009; Song et al., 2012). The hazard score is based on clearly reported study data and therefore is reported with high confidence. A Moderate concern (high confidence) was assigned for oral exposure based on the Kim (2010) study. A DG for dermal exposure was assigned; although a dermal study was performed and summarized below this information could not be converted into a hazard rating.

- Authoritative and Screening Lists
  - *Authoritative: Not on any authoritative lists*
  - *Screening:*
    - *Japan - GHS - Specific target organs/systemic toxicity following repeated exposure - Category 1*

Oral

- Groups of 5 male and female ICR mice were exposed to nanosilver (22 nm, 42 nm, 71 nm, or 323 nm in deionized water) orally administered in 1 mg/kg for 14 days. The control group was exposed to deionized water only. Tissue distribution was measured in the brain, lung, liver, kidney and testis after 14 days?. Silver was not detected in the control mice, or in the group treated with the largest size AgNPs (323 nm), however it was significantly accumulated with the highest amounts in the 22nm group for all tissues (p < 0.01). Tissue levels were inversely related to AgNP size, and decreased at 42 nm and 71 nm respectively for the brain, lung, and testis. The inverse size response was not as clear in the liver and kidney with levels from highest to lowest at 22 nm, 71 nm, and 42 nm, respectively. Authors suggested smaller sized AgNP could be more easily absorbed in the GI tract and transported to organs. TGF-β was measured in serum after 14 days, and levels were significantly increased by the greatest amount for the 22nm group, followed by 42 nm and 71 nm, show a clear size-related response. TGF-β was not increased in the control or 323 nm group. Analysis of lymphocyte phenotypes and T cell subtypes after oral administration of the AgNPs suggested a size-related response with values slightly higher in the group with the smaller size AgNPs [note reported details do not include statistical significance]. There were no significant changes in body weight gain, organ weights, or histopathological findings for any group (Park et al., 2010). A NOAEL was not determined, all exposures were at the same mass dose, and this information does not necessarily relate to a specific GreenScreen™ hazard endpoint.
- Groups of 6 male and female ICR mice were exposed to nanosilver (42 nm in deionized water) at 0.25, 0.5, or 1 mg/kg by gavage?oral administration for 28 days in a non-guideline repeated dose toxicity test. The control group was exposed to deionized water only. Body weight was measured on days 0, 7, 14, 21, and 28. Blood samples were taken when?from 3 mice per group for cell phenotype and biochemistry (total protein, albumin, aspartate aminotransferase (AST), alanine aminotransferase (ALT), alkaline phosphatase (ALP), creatinine, and BUN., and from the other 3 mice for a cytokine assay. Mice were then sacrificed for analysis of tissue distribution and histopathology (kidney, liver and small intestine). ALP and AST was significantly increased in males and females at 1 mg/kg bw/d, and ALT was increased in females only at 1 mg/kg bw/d. There were no significant findings in remaining biochemical analyses. Levels of pro-inflammatory cytokines were dose-dependently increased with p < 0.01 for IL-1, IL-6, IL-10, IL-12, and TGF-β, and p < 0.05 for IL-4 and IgE. Results were not statistically different from controls with the TNF-α, IL-5 and IFNr. Increases in NK, NKT, B, and T cell distributions were slightly increased in all treated groups compared to controls [p-value not reported]. Histopathological analyses revealed slight cell infiltration [not quantified] in the cortex of the kidneys for exposed males and females, and no other findings in the livers or small intestines (Park et al., 2010). A NOAEL/LOAEL was not determined and cannot be inferred, this was not a guideline study, findings are not definitively related to a specific GreenScreen™ hazard endpoint.
- A 90-day repeated dose study was performed in five week old male and female Fisher 344 rats per OECD 408 and GLP. Daily oral doses of silver nanoparticles were administered by gavage in 0.5% carboxymethylcellulose (CMC) for 13-weeks to groups of 10/dose, at 0, 30, 125, or 500 mg/kg/day. The silver nanoparticles (CAS no 7440-22-4) were at least 99.98% pure and had a geometric mean particle diameter of 56 nm (GSD = 1.46 nm). After 90 days of exposure, clinical chemistry, hematology, histopathology, and silver distribution were studied.
- There were no significant differences in food consumption and water intake between treated male and female rats and the control group. There were no significant dose-related changes in the body weight of female rats; however, there were significant dose-related decreases in the body weight of high-dose male rats at 4, 5, and 7 weeks of exposure at the conclusion of the study 13 weeks, and middle-dose male rats at 10 weeks of exposure (magnitude not reported. No significant organ-weight changes were observed in either the male or female rats after 90 days except for an minimal increase in the weight of the left testis for the high dose male rats (though statistically significant, the severity was minimal with weights of 0.47 +/- 0.02 for controls v. 0.51 +/- 0.01 for high dose group), and for decreases in the weight of right kidney for the low-and middle-dose female rats but not the high dose females.
- There appeared to be increased alkaline phosphatase for male rats in the middle and high-dose groups but was not statistically significant. However, there was a significant increase in alkaline phosphatase for female rats in the high-dose groups. A significant increase in cholesterol was also found in the middle and high-dose male rats and the high-dose female rats. A significant increase in total bilirubin was noted in the middle-dose male rats. A significant decrease in magnesium, protein, and inorganic phosphorus was found in the middle and high-dose female rats. No significant changes in the hematological parameters were noted except for a significant increase in monocytes in the high-dose female rats. The reticulocyte count for the low-dose female rats decreased significantly when compared with the control group. Coagulation time in terms of the active partial thromboplast time and prothrombin time did not show any significant changes when compared with the control group.
- There was a statistically significant (P < 0.01) dose dependent increase in the silver concentration of all the tissue samples from the groups exposed to silver nanoparticles in this study. In addition, a two-fold higher accumulation of silver in the kidneys of female rats when compared with the male rats occurred across all the dose groups indicating a marked gender-dependent distribution. In the liver, minimal bile-duct hyperplasia was identified in 4/10, 7/10, 8/10, and 6/10 of control, low, middle, and high-dose males, as well as 3/10, 7/10, 8/10, and 7/10 of the control, low, middle, and high-dose females, respectively. Focal, multifocal, or lobular necrosis was noted in 0/10, 4/10, 5/10, and 4/10 of the control, low, middle, and high-dose male, and 0/10, 2/10, 2/ 10, and 2/10 of the control, low, middle, and high-dose females, respectively. Although there appeared to be a slight increase of minimal tubular basophilia in the kidneys of high-dose male rats, these changes were not statistically significant and thus not considered to be test article exposure related. Tubular basophilia were also more prevalent in the male rats compared to the female rats. Minimum or mild renal unilateral or bilateral mineralization was observed in 5/10, 8/10, 7/10, and 9/10 of the control, low, middle, and high-dose female rats, respectively. Histopathologic examination of lung tissue did not show any treatment-related effects. In the intestines, pigmentation of villi was observed in 0/10, 0/10, 8/10, and 8/10 of the control, low, middle, and high-dose male rats, respectively. This dose-dependent increase in the pigmentation of the villi indicated an apparent treatment-related effect.
- The authors noted that the tissue distribution of silver particles and the liver as the target organ are consistent with previous oral (Kim et al., 2007) and inhalation (Sung et al., 2009) studies where smaller silver particles were used (18-19 nm instead of 56 nm), indicating that distribution and toxicity do not appear to be dependent on particle size in this tested range. The authors suggested a LOAEL of 125 mg/kg and a NOAEL of 30 mg/kg (Kim et al., 2010). It is not clear what the authors used as the critical effect, it would appear to be the minimum liver effects (hyperplasia and necrosis) in combination with histological findings of increased cholesterol at 125 mg/kg and higher. It may also be noted the decreased body weight gain of approximately 10% (estimated graphically) for high dose male rats may have been toxicologically significant noting there were no differences in food consumption). NSF suggests there were adverse effects seen at all doses, a NOAEL was not determined, and the LOAEL should have been 30 mg/kg, based on hyperplasia and necrosis in the liver, and mineralization in the kidneys. Without a NOAEL, this study approximates GHS Category 2 and GreenScreen™ Moderate concern for repeated dose toxicity – oral exposure.
- Groups of Sprague Dawley rats were exposed to nanosilver (average 60 nm, range 52.7-70.9 nm, 99.98% pure) at doses of 0, 30, 300 or 1000 mg/kg-bw/day by gavage in 0.5% aqueous carboxymethylcellulose for 28 days, per OECD 407. Observations included clinical signs, body weight, haematology, blood chemistry, organ weights, microscopy, tissue distribution (determination of silver concentration in blood, lungs, kidneys, brain, stomach, liver and testes). There were no observed effects on body weights or organ weights, except for an increase in brain weight in high dose males. Some statistically significant changes were observed in hematology (increases in RBC, Hb, Hc and decrease in blood coagulation time in females at mid and/or high dose, and increase in MCV in high dose males) and biochemistry parameters (increases in ALP and cholesterol in mid- and/or high-dose males and females). Histopathological changes in the liver of males and females included dose-dependent bile-duct hyperplasia, dilation of the central veins and inflammation (authors did not specify which dose groups had these effects). Silver concentrations were dose-dependent in blood and all examined tissues, with the kidney concentrations in females being 2-fold higher than males (Kim et al., 2008). A NOAEL and LOAEL were not reported and cannot be inferred. Cannot apply a GreenScreen™ rating – informational only. The RIVM (2009) review of this study noted no information was provided on the magnitude and form/shape of nanoparticles in the dosing solution. Furthermore, where the study was stated to have been performed per OECD 407, neurotoxicity parameters were not investigated, and histopathology did not include examination of spinal cord, peripheral nerve, bone marrow, lymph nodes, stomach or small or large intestines. It is also unclear whether representative regions of the brain were examined histopathologically.
- Wistar rats were exposed by oral gavage 2x/day for 28 days to 11.5 mg/mL of either Polyvinylpyrrolidone (PVP, vehicle control, N=9), AgNPs + PVP (N=9), or AgAc + PVP (N=7). One additional rat per group was included for tissue sampling for electron microscopy. The daily dose of silver in the AgNPs and AgAc groups was 9 mg/kg-bw. There is no citation of a method guideline and no reference to GLP. AgNP particle size was 14 +/- 4 nm (90% by volume). The organ distribution pattern of silver following administration of AgNPs and AgAc was similar; however the absolute silver concentrations in tissues were lower for AgNps compared to AgAc, which corresponded with higher fecal concentrations for AgNPs. The largest silver concentrations were detected in the liver and kidneys, smaller amounts were detected in the lungs and brain. Granules detected in the lysosomes consisted of silver, selenium, and sulfur for both AgNP and AgAc exposed rats and the diameter of the deposited granules was in the same size range as the administered AgNP’s, suggesting a common mechanism of formation for AgNPs and AgAc (Loeschner et al., 2011). This study demonstrates that silver is bioavailable following oral exposure to AgNP (PVP coated). It has not been determined whether AgNPs become absorbed and translocate as intact nanoparticles to organs and tissues, or if they dissolve in the gastrointestinal system prior to absorption. Does not translate to a GreenScreen™ value, informational only.

Dermal

- Groups of 6 male Hartley albino guinea pigs were exposed to aqueous solutions of colloidal nanosilver (<100 nm as confirmed by TEM) at 0, 100, 1,000 or 10,000 μg/mL (0, 0.1, 1, or 10 mg/L), or silver nitrate at 0.1 mg/L, applied to an area of 5 cm x 5 cm on the back in a shaved region. The application was performed twice daily 5 days/week for 13 weeks. Animals were evaluated for clinical signs, and histopathology was performed on tissues form the skin, liver and spleen for 3 animals/group. Animals exposed to 0.1 mg/L nanosilver had decreased thickness of epidermis and dermis, increased levels of Langerhans cells, inflammation, and decreased papillary layer with regular collagen fibers. Mid dose animals had the same effects as the low dose group, as well as increased levels of round cells, and acidophilic cytoplasm in muscle fibers with inflammation also in the endomysium, and some muscle fibers were surrounded by macrophages. High dose animals had the same effects as lower dose animals, as well as muscle fibers with acidophilic cytoplasm were surrounded by macrophages, and in addition to degenerative fiber, increased levels of macrophage in endomysium were observed with inflammation. Authors suggest all of these histopathological changes were adverse and dose- and time-dependent. In the liver, animals exposed to nanosilver and to silver nitrate had destruction of hepatocytes cords, overproduction of Kupffer cells, and degeneration of hepatocytes. Necrosis was observed only at the highest dose group for nanosilver, 10 mg/mL. In the spleen, red capsules and white pulp were seen regularly in the control animals without any abnormal change or inflammation. In the silver nitrate group, red capsules were thinner, there was inflammation, and white pulp hypertrophy. The low-, mid-, and high-dose nanosilver groups had thinner red capsules with signs of inflammation, accumulation of red blood cells, and white pulp atrophy, with severity being greatest at the highest dose (Korani et al., 2011). A NOAEL was not determined, and the LOAEL cannot be quantified in terms of mg/kg bw/d because the total mass applied was not reported. Informational only.

Inhalation

- Sprague Dawley rats 10/sex/dose were exposed to nanosilver (1.98-64.9 nm, average 18-19 nm) by inhalation (whole-body) at doses of 0, 0.48 µg/m^3^, 3.48 µg/m^3^, or 61.24 µg/m^3^ (authors calculated doses from number particles/cm^3^ and geometric mean diameter) 6 hr/day, 5 d/week, for 4 weeks, per OECD 412. Observations included clinical signs, body weight, hematology, blood chemistry (22 parameters), organ weights and microscopy (21 tissues/organs, including brain, lungs, reproductive organs), and determination of silver concentration in blood, lungs, brain, olfactory bulb, and liver (in 5/sex/dose). No distinct clinical effects, effects on body weight or organ weight changes were observed. There were only very few small, statistically significant changes in hematology and biochemistry parameters observed, with a slight increase in blood calcium being the only consistent parameter affected in both males and females (high dose only). Increases in cytoplasmic vacuolization were statistically significant, but were only dose related in females (2 cases each in the control and low dose groups, 6 in the mid-dose, and 7 cases in the high dose group). Two cases of hepatic focal necrosis were detected among male rats in the high dose group and one case among female rats in the high dose group. Silver was not detected in blood, but the silver concentration was increased in lungs, liver, olfactory bulb and brain. No other histopathological findings were reported. Authors suggested the vacuolization and hepatic focal necrosis were not dose related and were often found in the normal rat population (Ji et al., 2007). However, as the toxicological significance of the calcium increase is not clear, NSF concluded a NOAEC of 0.48 µg/m^3^ and LOAEC of 3.48 µg/m^3^ based on liver histopathology (cytoplasmic vacuolization in females at mid-dose). Although RIVM (2009) noted OECD 412 does not include detection of neurotoxic potential and has limited pathology (e.g. no specific examinations of respiratory system), OECD 412 is an acceptable method for evaluation against GHS and DfE criteria. This equates to GHS Category 1 (0.48 µg*/*m^3^ = 0.00048 µg*/*L) and High concern for repeated dose inhalation toxicity per the GreenScreen™ criteria.
- Sprague-Dawley rats 10/sex/dose were exposed to nanosilver (18 nm) by inhalation (whole body) at 0, 49 μg/m^3^, 133 μg/m^3^ or 515 μg/m^3^ 6 hours/day, 5 days/week, for 13 weeks in a while body inhalation chamber per OECD 413 and GLP. Silver nanoparticles were confirmed by TEM to be spherical in shape and nonaggregated/agglomerated with diameters under 55 nm, with median diameter 18 nm, and GSD 1.5.
- There were no statistically significant gross effects observed for food consumption, body weights, or organ weights for either sex. There were no significant dose-related differences in hematology values or blood biochemical parameters. Silver concentrations were increased and dose related in the lung, blood, liver, brain and olfactory bulb for both sexes. Silver concentration in the kidneys showed a gender difference with females having two to three times more accumulation than males. In the liver, minimal bile-duct hyperplasia was identified in 0/10, 0/10, 1/10, and 4/9 for the control, low, mid, and high-dose males, respectively. One of the high dose males with minimal bile-duct hyperplasia also had minimal portal mineralization. For females, minimal bile-duct hyperplasia was identified in 3/10, 2/10, 4/10, and 8/10 for the control, low, mid, and high dose groups, respectively. In high dose females, 3/10 also showed single-cell hepatocellular necrosis characterized by increased cellular eosinophilia and shrunken condensed nuclei. One high dose female had moderate bile-duct hyperplasia with concurrent moderate centrolobular fibrosis, minimal single-cell hepatocye necrosis, mild pigment accumulation, and moderate multifocal necrosis. Histopathological examination of the lung samples revealed a high incidence of minimal alterations, including some chronic alveolar inflammation, a mixed cell perivascular infiltrate, and alveolar macrophage accumulation in the high-dose males and females, compared to controls. No significant histopathological findings were noted in the nasal pathways. In the kidneys, minimal tubular basophilia was noted in all groups, including controls, and was not considered test-related. Similarly, in the heart minimal degeneration/necrosis was observed in all groups including controls and was not considered test related. Authors concluded a 90-day inhalation, 6hr/day NOAEC of 100 µg/m^3^ (Sung et al., 2009). However, as the reported NOAEC was not one of the tested concentrations, NSF concluded a NOAEC of 133 µg/m^3^ (0.000133 mg/L), and LOAEC of 515 µg/m^3^ (0.000515 mg/L), based on bile-duct hyperplasia in the liver and inflammation in the lungs for both sexes. This equates to GHS Category 1 – high concern for inhalation toxicity.
- A sub-acute murine inhalation study examined the lung tissue of male C57B1/6 mice for signs of inflammation after 10-days exposure to nanosilver (5 +/- 2 nm) at 3.3 mg/m^3^ 4hrs/day x 10 days in a water-based aersol. Toxicity was assessed by enumeration of total and differential cells, determination of total protein, lactate dehydrogenase activity and inflammatory cytokines in bronchoalveolar lavage fluid, and lungs were evaluated for histopathologic changes and the presence of silver. Dissolution studies showed that nanosilver did not dissolve in solutions mimicking the intracellular or extracellular milieu. Exposed mice showed minimal pulmonary inflammation or cytotoxicity. This was not an OECD method but was performed to examine the possibility that *in vivo* toxicity is less pronounced than *in vitro* toxicity for lung inflammation (Stebounova et al., 2011). Does not translate to a GreenScreen™ value, informational only.
- Sprague-Dawley rats 10/sex/dose were exposed to nanosilver (18 nm) by inhalation (whole body) at 0, 49 μg/m^3^, 133 μg/m^3^ or 515 μg/m^3^ 6 hours/day, 5 days/week, for 13 weeks. This was not a guideline method, but was intended to examine inflammatory responses and pulmonary function changes. Lung function tests were conducted weekly on 4 rats/dose, parameters included the tidal volume (TV, ml), minute volume (MV, ml/min), respiratory frequency (BPM, breath/min), inspiration time (Ti, s), expiration time (Te, s), peak inspiration flow (PIF, ml/s), and peak expiration flow (PEF, ml/s). The same animals that underwent the lung function test were also subjected to a bronchoalveolar lavage after the 90 days of silver nanoparticle exposure. The cells were smeared, then stained to take a count of the total cell numbers, macrophages, polymorphonuclear cells (PMNs), and lymphocytes. The bronchoalveolar lavage (BAL) levels of total protein, albumin, and LDH were also measured.
  - When compared to the control group, all the exposed groups showed elevated total cell numbers, alveolar macrophages, polymorphonuclear cells (PMN), and lymphocytes in the male rats, while no elevation was noticeable in the female rats. The albumin, LDH, and total protein as inflammatory markers in the BAL were all increased in the female rats from the high-dose group; however, there were no significant increases in the male rats.
  - Among the pulmonary function test parameters, the tidal volume, minute volume, and peak inspiration flow showed significant changes during the 90 days of silver nanoparticle exposure. The dose-dependent tidal volume decreases in the male rats led to minute volume and peak inspiratory flow decreases in the high-dose group. The tendency of a dose-dependent decrease in the tidal volume also appeared in the female rats. All the exposed groups of female rats exhibited decreased minute volumes and peak inspiration flows compared with the control.
  - The histopathological examinations of the lung samples from the rats exposed to the silver nanoparticles for 90 days showed significantly increased incidence of mixed cell infiltrate perivascular and chronic alveolar inflammation, including alveolaritis, granulomatous lesions, and alveolar wall thickening and alveolar macrophage accumulation.
  - The authors concluded that the decreased lung function (tidal volume, minute volume and peak inspiration flow) was related to inflammatory lesions and inflammatory markers (BAL analyses), and that deposition in the respiratory system is inversely correlated with particle size. The discussion section of the article confirmed that the particles were nonaggregated and nonagglomerated. The surface area of the particles ranged from 1.08 x 10^9^ to 6.43 x 10^9^ in the breathing zone (Sung et al., 2008). This study is not suitable for direct comparison to the GreenScreen, it was not a guideline method and a NOAEL was not determined. A guideline follow-up study by same authors is summarized above.
- This study was a follow-up to Sung et al (2008, summarized above), that was designed to investigate the recovery from the lung effects noted in the previous study. Six week old male and female Sprague Dawley rats were divided into 4 dose groups (each group consisted of 17 male rats and 12 female rats) and exposed to silver nanoparticles 14-15 nm in diameter in an inhalation chamber in the following concentrations: a control group, a low-dose group (0.6 x 10^6^ particles/cm^3^ or 49 µg/m^3^), a medium dose group (1.4 x 10^6^ particles/cm^3^ or 117 µg/m^3^), and a high dose group (3.0 x 10^6^ particles/cm^3^ or 381 µg/m^3^). The silver nanoparticles were spherical in shape and non-aggregated/non-agglomerated with diameters under 47 nm. Four animals from each group were used to monitor specific time points: after 12 weeks of exposure, after 4 weeks of recovery, and after 12 weeks of recovery. An additional five male rats were also assigned to the 12-week exposure group for a study on brain translocation. The animals were examined daily on weekdays for exposure related effects including respiratory, dermal, behavioral, nasal, or genitourinary changes suggestive of irritancy. Animals were weighed once a week during the inhalation exposure.
  - Lung function tests were conducted on four rats from each dose group. The parameters for the pulmonary function test included the tidal volume (TV, ml), minute volume (MV, ml/min), respiratory frequency (BPM, breath/min), inspiration time (Ti, s), expiration time (Te, s), peak inspiration flow (PIF, ml/s), and peak expiration flow (PEF, ml/s). After the exposure and recovery periods, the rats were anaesthetized and blood samples were collected. The lungs, trachea, and other organs were fixed and examined.
  - No significant toxicity or signs of mortality were observed in any dose group, and no significant (biologically relevant) effects on food consumption or body weight were noted. There were statistically significant decreases in MV, PIF, PEF, and TV among the male rats in the middle and high dose groups at time points throughout both the exposure period and the recovery period. In contrast, there were no exposure-related statistically significant changes in lung function among female rats. There was a statistically significant dose-dependent increase in lung tissue silver concentration in both males and females after the exposure period, which showed a trend of clearing over the 12 week recovery period, but was not completely cleared by the end of the period. Most other tissues showed a similar trend, but a significant accumulation compared to the control group was still noted in the liver and spleen in male rats after 12 weeks of recovery, and the kidneys in female rats contained five times more accumulated silver than the male kidneys.
  - “Histopathological examination of lung samples from the rats exposed to the silver nanoparticles for 12 weeks revealed a significant increase in the incidence of mixed cell infiltrate perivascular and chronic alveolar inflammation, including alveolaritis, granulomatous lesions, and alveolar wall thickening and alveolar macrophage accumulation in both the male and female rats. During the 12 weeks of recovery, the female rats in all the groups showed a gradual clearance of the silver nanoparticles and decreased inflammation. However, the male rats in the high-dose group did not achieve a complete recovery, exhibiting persistent inflammation throughout the 12-week recovery period” (Song et al., 2012).
  - The authors were not able to definitively explain the gender difference in the toxicological response, but pointed out that female sex hormones may influence lung function and that testosterone may play a role in airway hyperresponsiveness. The middle dose (117 µg/m3 or 0.000117mg/L) was suggested as the NOAEL, as the inflammation seen at this dose was considered an adaptive response and not a sign of overt toxicity.

**Neurotoxicity (N)**

**(N-single) Group II Score (single dose: vH, H, M or L)** DG

Nanosilver was assigned a score of **Data Gap** for neurotoxicity based on no relevant data.

- Authoritative and Screening Lists
  - *Authoritative: Not on any authoritative lists*
  - *Screening: Not on any screening lists*
- Fifty-six 12-week old female balb/c mice were randomly divided into two groups of equal sizes (experimental and control group). The exposed group received two intracranial injections of 5 μl of silver-sodium hyaluronate within 1-2 min, and the control group received similar injections of sodium hyaluronate. Sodium hyaluronate was used as a vehicle based on its viscosity and the fact that it is known to be well tolerated. The two injections of 5 µl of the suspended silver suspension corresponded to 1.18 mg silver per animal. After 7 days, 14 days, and 9 months post exposure, sub-groups of 8, 10, and 10 animals (respectively) from the experimental cohort were perfused and examined via autometallography, immunohistochemistry, and electron microscopy. Particles were less than 20 μm in size.
  - Injection of metallic particles in neural tissue in the brain led to a large cavity formation near the injection site at the telencephalon, indicating severe tissue loss. After nine months, the lesion tract was completely healed with scar tissue formation. Control animals showed no such cavity formation. Injection also resulted in autometallographic staining of neurons and glial cells, which was most prominent in the ipsilateral cortex and hippocampus. A temporary TNF-alpha response was also observed, which was most prominent at the bottom of the injection tract in the deep cortex, which had almost completely dissipated after nine months. Controls showed very little TNF-alpha activity. The injection also increased microgliosis, astrogliosis, and metallothionein expression relative to controls. However, few apoptotic cells were observed 7 days post-injection, and no apoptotic cells were observed 14 days and nine months post-injection. The authors concluded that the study illustrates that metallic silver fragments cause inflammation and massive tissue loss in the brain after just a few weeks of exposure. The toxicological response in terms of tissue loss continued over time (Locht et al, 2011). This non-standard method is not applicable for comparison to GreenScreen™ criteria. Informational only.

**(N-repeat) Group II* Score (repeated dose: H, M, L):** *M*

Nanosilver was assigned a score of **Moderate** concern (low confidence) for repeat dose neurotoxicity based on effects in rats and mice following intranasal instillation, subcutaneous treatement, and intragastric administration. The hazard score is based on non-standard routes of exposure or non-standard neurotoxicity endpoints and therefore is reported with low confidence.

- Authoritative and Screening Lists
  - *Authoritative: Not on any authoritative lists*
  - *Screening: Not on any screening lists*
- Neonatal rats, initially weighing 4 - 5 g, were used as a control or treated with 0.1, 0.2, 0.5, and 1 mg/kg/day AgNP nasal drops via intranasal instillation once daily for 14 consecutive weeks. AgNPs (1 mg/mL) were coated with citrate. Transmission electron microscopy (TEM) characterization was carried out to obtain the primary size, size distribution, and morphology of the AgNPs. Both size and zeta potential were measured three times for accuracy. Intranasal instillation of AgNPs in neonatal Sprague- Dawley rats caused significant body weight loss. Moreover, histological examinations revealed activation of neuroglial cells with concomitant destruction of the granular layer of the cerebellum at 1 mg/kg/day. Furthermore, western blot analyses showed an increase in the levels of the glial fibrillary acidic protein (GFAP), a marker of astrocyte activation. These observations suggest that AgNPs have significant neurotoxic effects on the rat cerebellum (Yin 2015).
- It is known that the biological half-life of silver in the central nervous system is longer than in other organs. However, the potential toxicity of silver nanoparticles (NPs) on brain tissue and the underlying mechanism(s) of action are not well understood. In this study, neurotoxicity of silver NPs was examined in rat after intragastric administration. After a two-week exposure to low-dose (1 mg/kg, body weight) or high-dose (10 mg/kg) silver NPs, the pathological and ultrastructural changes in brain tissue were evaluated with H&E staining and transmission electron microscopy. The mRNA expression levels of key tight junction proteins of the blood–brain barrier (BBB) were analyzed by real-time RT-PCR, and several inflammatory factors were assessed in blood using ELISA assay. Observations included neuron shrinkage, cytoplasmic or foot swelling of astrocytes, and extravascular lymphocytes in silver NP exposure groups. The cadherin 1 and Claudin-1 were slightly increase in mRNA expression levels, and IL-4 significantly increased after silver NP exposure. It was suggest that silver NP can induce neuronal degeneration and astrocyte swelling, even with a low-dose (1 mg/kg) oral exposure. One potential mechanism for the effects of silver NPs to the nervous cells is involved in inflammatory effects. (Xu 2015)
- Male Wistar rats were orally exposed once daily via the gastric tube at a dose of 0.2 mg/kg b.w. per day for 14 days to citrate-stabilized silver nanoparticles (defined by the manufacturer as a colloidal solution of nanoparticles 10 - 4 nm in diameter, stabilized in sodium citrate). The effect of prolonged exposure on synapse ultrastructure and specific proteins was observed. Administration of both nanosilver and ionic silver over a two-week period resulted in ultrastructural changes including blurred synapse structure and strongly enhanced density of synaptic vesicles clustering in the center of the presynaptic part. Disturbed synaptic membrane leading to liberation of synaptic vesicles into neuropil, which testifies for strong synaptic degeneration, was characteristic feature observed under AgNPs exposure. Also a noteworthy finding was the presence of myelin-like structures derived from fragmented membranes and organelles which are associated with neurodegenerative processes. Additionally, observations included significantly decreased levels of the presynaptic proteins synapsin I and synaptophysin, as well as PSD-95 protein which is an indicator of postsynaptic densities. The authors concluded that this study demonstrates that exposure of adult rats to both forms of silver leads to ultrastructural changes in synapses. In addition the authors suggest tha that small AgNPs lead to more severe synaptic degeneration, mainly in the hippocampal region of brain. The observations may indicate impairment of nerve function and, in the case of hippocampus, may predict impairment of cognitive processes (Skalska 2014).
- Thirty virgin female NMRI mice were mated and treated subcutaneously once every three days from gestation day 3 until delivery, by 0, 0.2 and 2 mg/kg of bodyweight (BW) of Ag-NPs. The
- nanoparticles had been prepared by the sol-gel method in which silver ions were reduced by sodium borohydride in the presence of citrate as stabilizer. Behavioral functions of adult offspring including spatial memory, passive avoidance learning, stress, anxiety-like behaviors and locomotoractivities were assessed by commonly used neurobehavioral paradigms and the results were compared according to treatment and sex. Prenatal exposure to Ag-NPs significantly impaired their cognitive behavior in the Morris water maze. Although no evidence was observed indicating more anxiety-like behaviors in the treated offspring in the elevated plus maze, the number of defecations and leanings in the open field assay and number of passages in the light-dark box were greater in groups prenatally treated by Ag-NPs. Most of the impairments were more apparent in the offspring which had been prenatally exposed to high doses of Ag-NPs, particularly female ones. The present study indicated that the exposure of pregnant animals to Ag-NPs may lead to various neurobehavioral disorders in their offspring (Ghaderi 2015).
- Lee et al. (2012b) detected Ag-NPs in various organs, including the brain of 4 day-old pups following oral administration to pregnant dams.

**Skin Sensitization (SnS) Group II* Score (H, M or L): L**

Nanosilver was assigned a score of **Low** concern (high confidence) for skin sensitization based results from an OECD 406 study. This score is based on a well reported guideline study and therefore reported with high confidence.

- Authoritative and Screening Lists
  - *Authoritative: Not on any authoritative lists*
  - *Screening:*
    - *Japan - GHS - Skin sensitizer - Category 1*
- AgNP (10 nm) suspended in 1% citric acid solution weakly sensitizing in 1/20 (5%) SPF guinea pigs in the guinea pig maximization test (OECD 406), exhibited as discrete and patchy erythema (Kim et al., 2012). GHS guidelines for an adjuvant-based guinea pig test method identify a response in >30% of the animals as positive, therefore this results would not require classification and may be considered Low concern for the GreenScreen™.
- “From human experience there are no indications for skin sensitization when nanosilver is applied to (intact) skin” (RIVM, 2009).
- No data found in lit search, or the FDA-CDER website. While nanosilver is not approved for OTC drug product use (FDA, 1999), at least one product, Curad™ Silver Bandages, is currently marketed for such (GIT, 2006).
- The Japanese NITE classification for skin sensitization is “Category 1, warning, may cause allergic skin reaction” based on descriptions that powder exposure causes allergic contact dermatitis as reported by ACGIH (2001) and that contact to accessories containing silver produced allergic reactions as report in Patty’s Industrial Hygiene (5th, 2001) (NITE, 2006). [ACGIH (2001) is addressed below, and Patty’s (2001) is a secondary reference. Neither of these it suitable to assign a hazard].

**Respiratory Sensitization (SnR) Group II* Score (H, M or L):** DG

Nanosilver was assigned a score of **Data Gap** for respiratory sensitization based on lack of data.

- Authoritative and Screening Lists
  - *Authoritative: Not on any authoritative lists*
  - *Screening: Not on any screening lists*

**Skin Irritation/Corrosivity (IrS) Group II Score (vH, H, M or L): L**

Nanosilver was assigned a score of **Low** concern (high confidence) for skin irritation/corrosivity based on results from an OECD 404 test. This score is reported as high confidence as it is based on a well reported guideline study.

- Authoritative and Screening Lists
  - *Authoritative: Not on any authoritative lists*
  - *Screening: Not on any screening lists*
- AgNP (10 nm) suspended in 1% citric acid solution was not irritating in the skin of New Zealand White Rabbits per OECD 404 test (Kim et al., 2012).
- Eight different AgNP’s were used to examine the cytotoxicity of AgNPs in human epidermal keratinocytes (HEKs) and their inflammatory and penetrating potential into porcine skin *in vivo*. Unwashed/uncoated (20,50, and 80 nm) AgNP’s suspended in deionized water, washed/uncoated (20, 50, and 80nm) AgNP’s suspended in deionized water, and dried carbon-coated (25 and 35 nm) were topically applied for 14 consecutive days. Unwashed AgNPs resulted in dose-dependent decrease in HEK viability, however washed and carbon-coated AgNP’s had no effect. Unwashed AgNPs resulted in a significant increase in release of proinflammatory mediators interleukin (IL)-1β, IL-6, IL-8 and tumor necrosis factor TNF-α concentrations. All types of AgNPs were observed in the cytoplasmic vacuoles of HEKs. Macroscopic observations showed no gross irritation in porcine skin, whereas microscopic and ultrastructural observations showed areas of focal inflammation and localization of AgNPs on the surface and in the upper stratum corneium layers of the skin (Samberg et al., 2010). This study has limitations in usefulness for the GreenScreen™, including a non-standard method, no relative scoring, and insufficient characterization of the test substance. Informational only.
- Guinea pigs were exposed to 50 or 100,000 ppm AgNP for 24 hours under occlusion to examine skin irritation, per OECD 434. Purity was measured at 99.96%, particle size 10-20 nm by TEM, and UV-vis spectrometry showed a narrow distribution suggesting low agglomeration. There was no evidence of immunological or cellular changes of the skin (Maneewattanapinyo et al., 2011).

**Eye Irritation/Corrosivity (IrE) Group II Score (vH, H, M or L): L**

Nanosilver was assigned a score of **Low** concern (high confidence) for eye irritation/corrosivity based on an OECD 405 study. This score is reported as high confidence as it is based on a well reported guideline study.

- Authoritative and Screening Lists
  - *Authoritative: Not on any authoritative lists*
  - *Screening:*
    - *Japan - GHS - Serious eye damage / eye irritation - Category 2B*
- AgNP (10 nm) suspended in 1% citric acid solution was not irritating in the eyes of New Zealand White Rabbits per OECD 405 (Kim et al., 2012).
- No ocular effects were noted in the various whole-body inhalation exposure studies (summarized above), however they may not have been thoroughly examined and/or compared to controls.
- Guinea pigs were exposed to a single dose of 50 or 5,000 ppm AgNP solution in distilled water to examine eye irritation, per OECD 405. Purity was measured at 99.96%, particle size 10-20 nm by TEM, and UV-vis spectrometry showed a narrow distribution suggesting low agglomeration. There was some transient mild conjunctival irritation in some animals in the 5,000 ppm group at 24 hours, but no signs of toxicity were apparent at the end of the observation period (Maneewattanapinyo et al., 2011). The observed effects are not sufficient for classification under GHS 2B.
- The Japanese NITE classification for serious eye damage / eye irritation is Category 2B, warning, causes eye irritation, based on mild irritation in rabbits with recovery in 48 hours as reported in IUCLID (2000) (NITE, 2006).

**Ecotoxicity (Ecotox)**

**Acute Aquatic Toxicity (AA) Score (vH, H, M or L): vH**

Nanosilver was assigned a score of **very High** concern (high confidence) for acute aquatic toxicity based on GHS Category 1 classification in daphnia, fish and algae. This score is reported as high confidence as it is based on numerous well reported study data.

- Authoritative and Screening Lists
  - *Authoritative: Not on any authoritative lists*
  - *Screening:*
    - *New Zealand - GHS - 9.1A (algal) - Very ecotoxic in the aquatic environment*
    - *New Zealand - GHS - 9.1A (crustacean) - Very ecotoxic in the aquatic environment*
    - *New Zealand - GHS - 9.1A (fish) - Very ecotoxic in the aquatic environment*
- The toxicity of AgNP (contained 20∼21 wt% citrate as a capping agent of AgNPs with a diameter range of 5–25 nm) was assessed using Organization for Economic Cooperation and Development (OECD) test guidelines, including a “Daphnia sp., acute immobilization test,” “Fish, acute toxicity test,” and “freshwater alga and cyanobacteria, growth inhibition test.” Based on the estimated median lethal/effective concentrations of AgNPs, the susceptibility to the nanomaterials was different among test organisms (daphnia > algae > fish), suggesting that theAgNPs are classified as “category acute 1” for *Daphnia magna*, “category acute 2” for *Oryzias latipes*, and“category acute 1” for *Raphidocelis subcapitata*, according to the GHS (Globally Harmonized System of Classification and Labelling of Chemicals). The average 96-hour median lethal concentrations (LC50) of AgNPs for Oryzias latipes was estimated to be 1.8, the median effective concentrations (EC50s) of AgNPs was 0.012 and the 72hrs EC50 (concentration at which a 50% inhibition of the growth rate is observed) of AgNPs for the average specific growth rate of Raphidocelis subcapitata was calculated as 0.74mg/L (Sohn 2015).
- Acute toxicity of silver nanoparticle preparations was examined in Zebrafish (*Danio rerio*) using 14 different AgNP preparations. Toxicity was evaluated based on mortaility, hatching rate, and heart rate in accordance with OECD Test Guidelines 203, 210, and 212 [note results for heart rate and hatching are not GreenScreen™ endpoints and were not discussed further in this summary]. Test substance concentrations, stabilization agents, and physicochemical properties were monitored as contributing factors [note 2 thiol-stabilized AgNP’s and 1 rod-shaped powdered form were excluded here because they are out of scope]. The following measurements are derived from Table I and supplemental tables (Cunningham et al., 2013).

| Nanosilver form | MDD diameter (nm) | TEM diameter (nm) | DLS diameter (nm) | DLS diameter in freshwater (nm) | Zeta potential (mV) | Zeta potential in freshwater (mV) |
| --- | --- | --- | --- | --- | --- | --- |
| TSC1 | N/A | 46 ± 5 | 56.84 | 94.2 | -15.1 | -33.2 |
| TSC2 | N/A | 110 ± 15 | 148.6 | 187.4 | -16.2 | -38.6 |
| PVP1 | N/A | 52 ± 12 | 74.1 | 92.3 | -10.2 | -3.62 |
| PVP2 | N/A | 140 ± 12 | 182.4 | 248.3 | -12.7 | -6.7 |
| BIO1 | N/A | 48 ± 6 | 64.1 | 108.2 | -19.4 | 3.36 |
| BIO2 | N/A | 155 ± 17 | 180.7 | 260.8 | -23.4 | 3.69 |
| Powder1-SA | <100 | 78 ± 24 | 91 | 297.2 | -27 | -21.3 |
| Powder2-SA | <150 | 204 ± 38 | 225.3 | 312.1 | -16.4 | -14.7 |
| Powder 1-PC | 100-150 | 140 ± 23 | 151.2 | 212.7 | -19.3 | -12.8 |
| Suspension1 | 40 | 42 ± 2 | 58.2 | 124.1 | -21 | -18.5 |
| Suspension2 | 80 | 77 ± 8 | 101.3 | 184.6 | -25.1 | -19.5 |

TSC = trisodium citrate stabilized, PVP = polyvinylpyrolidone, Bio = gelatin.

Results showed dispersion and aggregation for all AgNP’s. TEM values show particle size and dispersion. The hydrodynamic diameters measured by DLS were compared to the TEM values. Diameter measurements for DLS were 1.2 to 2.0 times higher than those by TEM, and DLS in freshwater was > 3.0 time greater than TEM. These increases suggest particle aggregation was occurring in the media. The most notable shifts in zeta potential occurred with the TSC, PVP, and BIO solutions, and such shifts are expected as a result of changes in temperature, pH and salinity. Authors noted changes in the zeta potential were related to the capping agent and independent of the particle diameter. UV-vis analysis was also used to confirm the presence of an absorbance peak, and results were consistent with that expected for nanosilver.

The 24- and 48-hr LC50’s are presented in the following table, and show that mortality was strongly influenced by capping agent, zeta potential and Ag^+^ dissolution rate, with resulting LC50 values differing by 2 orders of magnitude (Cunningham et al., 2013):

| Nanosilver form | 24-h LC50 (ppm) | 48-h LC50 (ppm) |
| --- | --- | --- |
| TSC1 | 15.146 | 6.922 |
| TSC2 | 18.395 | 2.427 |
| PVP1 | 0.464 | **0.061** |
| PVP2 | 0.406 | 0.228 |
| BIO1 | 57.41 | 5.891 |
| BIO2 | 43.497 | 3.043 |
| Powder1-SA | 13.75 | 0.37 |
| Powder2-SA | 14.528 | 0.0455 |
| Powder 1-PC | 13.367 | 0.351 |
| Suspension1 | 5.28 | 3.455 |
| Suspension2 | 6.615 | 3.091 |

- Acute (48 h), chronic (21 d) and long-term effects of nanosilver (primary size 15 nm) on five successive generations of three Daphnia species (D. magna, D. pulex, and D. galeata) were investigated. Nanosilver dispersions were obtained by preparing a stock dispersion of NM-300. Acute EC50 values based on immobilization of nanosilver were 121 µg Ag L(-1) for D. magna being the least sensitive species and 8.95 and 13.9 µg Ag L(-1) for D. pulex and D. galeata, respectively (Volker 2013).
- The **48-hr EC50 in *C. daphnia* for powder type AgNP (60-100nm)** suspension, and using a water accommodated fraction (WAF), was 0.00075 mg/L for total Ag, 0.00037 mg/L for dissolved Ag. Comparatively, the 48 hr EC50 in *C. daphnia* for citrate-stabilized AgNP (average 13.3 nm) was 0.00798 mg/L for total Ag, and 0.00088 mg/L for dissolved Ag. Testing was performed per OECD 202. The WAF was prepared as follows, with the intent of removing aggregates: powders were added directly to the culture water at 100 mg/L, stirred for 24 h and settled for 48 hours, then filtered with a 0.45um membrane. The citrate stabilized silver was well dispersed in suspension and much smaller than 0.45 um, so it did not require filtration. Total Ag was measured after 48 hrs settling time, whereas dissolved Ag was determined by filtering (1-2nm membrane), centrifuging, then measuring by ICP-MS, which cannot distinguish ions from complexes (Lee et al., 2012).
- The acute toxicity of nanosilver powder (25.4nm) suspensions was evaluated in *Ceriodaphnia dubia* and *Pseudokirchneriella subcapitata*, each exposed in three different types of water. The water types varied in dissolved organic carbon (DOC) content, pH, and various ion concentrations. Mixtures were shaken for 1 weeks then filtered (1.6 um) to remove large aggregates. The obtained filtrates were then used to determine total concentrations of Ag by ICP-AES. The exposure method was EPA-821-R-02-013, Short-term methods for estimating the chronic toxicity of effluents and receiving waters to freshwater organisms. *C. dubia* were exposed at 25°C under constant aeration with a photoperiod of 16:8 light:dark, for 48 hours under static conditions. The endpoint was mortality and/or immobilization. *P. subcapitata* was incubated for 96 hours at room temperature under controlled light and shaken twice daily. Authors observed images from scanning electron microscopy which showed the silver apparently coated by the DOC within the ACT water, and speculated this serves a protective function resulting in lower acute aquatic toxicity under the test conditions. Comparatively, the nanosilver suspended in the SPG (Spring fed) water showed large and small aggregates with no evidence of organic matter coating (McLaughlin and Bonzongo, 2012). Results are summarized (by NSF) as follows:

**Summary of acute aquatic toxicity study results (Mclaughlin and Bonzongo, 2012)**

| Water designation | Water type | Average diameter of the suspended nAg (nm) | Zeta potential (mV) | *C. dubia* (water flea) 48hr EC50 (mg/L) | *P. subcapitata* (green algae) 96h IC50 (mg/L) |
| --- | --- | --- | --- | --- | --- |
| ACT | Wetland water (high DOC) | 76.8 | -28.8 | 221 | 1600 |
| SPG | Spring fed water (low DOC and moderate ionic strength) | 192 | -12.7 | 0.433 | 22.6 |
| CM | Culture medium (per EPA method) | 174 | -24.8 | N/A | 4.61 |
| MHW | Moderately hard water (traditional growth media) | 395 | -4.38 | 0.482 | N/A |

Comments: Methods for analysis using effluent and receiving waters are not comparable to OECD 202 or 201 (for daphnia and algae), however this study is important because it shows significant influence of the water parameters on nanosilver suspended particle size and also on EC/IC50 values.

- *Daphnia magna* were exposed to two colloidal suspensions of AgNP, a powder dispersion of AgNP, and AgNO_3_ (to test ionic silver) for 48 hours per OECD 202. One of the colloidal suspensions, referred to as nAg1, was a water-based colloid containing 200,000 mg/L spherical silver nanoparticles (5–25 nm, 99.98% purity), with confirmed 20.48 wt% silver nanoparticles, and 1.0 wt% citrate as the capping agent, at pH 5.80. The second colloidal type, nAg2, was a water-based colloid containing 4,000 mg/L spherical silver nanoparticles (average size 16.6 nm, purity not specified), pH 2.4. Due to the use of an anionic surfactant (linear alkyl benzene sulfonate) and lack of purity specification, nAg2 was considered outside the defined scope. The powdered AgNP dispersion, nAg3, was 99% pure spherical silver nanoparticles (average 20 nm), suspended in deionized water, followed by vortexing and sonication. In the case of the nAg3 suspension, despite extensive sonication, the TEM images showed that in an aqueous environment about 52.9% of the nanoparticles were clumped together and formed large aggregates, with 70.31% of the aggregates in the range of 25 to 100 nm, and most of the others in the range of 100 ~ 250 nm. During the exposure period, the mortality in the control groups was less than 5% for all the tests. The 48-hour EC50s for the two AgN colloids were 0.004 and 0.002 respectively, while the 48-hour EC50 for the AgNP suspension was 0.187 mg/L. The 48-hour EC50 for AgNO_3_ was 0.0023 mg/L. The authors proposed the lower toxicity with the nAg3 solution was likely due to the aggregation and decreased surface area to volume ratio (Asghari et al., 2012).
- A study by Kim et al (2011b) examined EC50 and LC50 values for *Daphnia magna* and *Oryzias latipes* (Japanese Medaka) exposed to silver nanoparticles. The study also attempted to confirm that the resulting toxicity is due to Ag^+^ ion released from the silver nanoparticles. An acute immobilization test for *D. magna* was carried out in accordance with OECD 202, and the acute toxicity test for *O. lapites* was carried out in accordance with OECD 203. Two suspensions of AgNP powder were used in the study: one with a mean particle diameter of 60 nm and the other with a mean particle diameter of 300 nm. In addition, the two organisms were also exposed to AgNO_3_ solution to compare the effects of AgNP to those of ionic silver. The author noted that rapid aggregation and agglomeration occurred in suspension, but many particles with diameters of < 100 nm were present in the water column at 48 hours, indicating that the potential for exposure to silver nanoparticles was present for at least an exposure of 48 hours. The EC50s were as follows:

48 h EC50, daphnia, AgNO3 = 0.5 mg Ag/L (95% CI = 0.4–0.6).

48-h EC50, daphnia, AgNP 60 nm = 1.0 mg Ag/L (95% CI = 0.1–1.3)

48-h EC50, daphnia, AgNP 300 nm = 1.4 mg Ag/L (95% CI = 0.3–2.1)

96 h LC50, latipes, AgNO3 = 21 mg Ag/L (95% CI = 15–30)

96 h LC50, latipes, AgNP 60 nm = 28 mg Ag/L (95% CI = 23–34)

96 h LC50, latipes, AgNP 300 nm = 67 mg Ag/L (95% CI = 45– 108)

- “The sorption efficiency of the synthesized sorbent was checked by spiking 0.5 mg/l Ag^+^ standard solution. The result, given in Table 1, showed that most silver ion was absorbed to the sorbent and very little silver was detected in the sample passed through the column. The sorption efficiencies of the column for 300-nm AgNP suspension were 98.8, 99.7 and 99.7%, which were lower than those for Ag+ standard solution. The calculated exposure concentrations of the column-passed 300 nm AgNP suspensions were 3.22, 0.34, and 0.72 mg/l in three experiments, respectively. Although the 48 h EC50 of 300 nm AgNP in the previous toxicity test was 1.4 mg/L, immobilization was not seen in any of the column-passed 300 nm AgNP suspensions at 48 h.”
- The authors concluded that AgNPs were acutely lethal to *D. magna* and *O. latipes* and would be classified as GHS category 1 for acute aquatic toxicity. The authors also noted that the EC50s/LC50s were similar to AgNO3, suggesting that the toxicity of AgNP suspensions is caused by Ag+ particles. The sorbent test described in the previous paragraph permitted daphnia exposure to AgNP without Ag+, and immobilization of daphnia did not occur in the 300 nm 0.00322 µg/L AgNP solution lacking Ag+. The authors stressed the need to examine the aggregates/agglomerates in the suspension of nanoparticles, which are believed to be non-ionic and non-toxic to aquatic life (Kim et al., 2011b).
- Gaiser et al (2011) examined acute 96-hour and chronic 21-day exposure of *Daphnia magna* neonates to silver particles. Nano and micro silver particles of size 35 nm and 0.6 – 1.6 μm, respectively, were used. However, in the reconstituted hard water used for D. magna culture and exposures, rapid aggregation occurred, and a high degree of polydispersity was evident. Mean sizes of particles in reconstituted hard water were 588 nm for nano-Ag, and 811 nm for micro-Ag.
- *Daphnia* neonates were exposed to 20 mL of 0, 0.01, 0.1, 1 and 10 mg/L of all particle types under semi-static conditions for acute exposures (96 h). For chronic exposures (21 d), the particle concentrations were based on the results of the acute toxicity studies and were 0, 0.001, 0.005, 0.01 and 0.05 mg/L. All exposure groups consisted of ten organisms each. *D. magna* were assessed for survival and molting (shedding of carapace) daily.
- “Ag particles caused mortality of *D. magna* neonates in a concentration-dependent manner. Nano-Ag particles exhibited higher toxicity than the micron-sized particles: 100% mortality occurred for both 10 and 1 mg/L of nano-Ag, and 56.7 ± 23.3% mortality at 0.1 mg/L for the nanoparticles. Treatment with micro-Ag resulted in a lower overall toxicity threshold with 100% mortality at 10 mg/L, 80 ± 20% mortality at 1 mg/L, and no significant toxicity at 0.1 mg/L.” The inferred 96-hour LC50 for the nanoparticles was approximately 0.1 mg/L, and the inferred 96-hour LC50 for the microparticles is in the range of 0.1 – 1 mg/L. The middle and high dose groups also showed reduced moulting frequency and reduced growth over the 96-hour period compared with controls. In the discussion, the authors cited a study on *C. dubia* and *D. pulex* where a much lower LC50 was observed (< 0.1 mg/L), but this difference was attributed to the lower incidence of particle aggregation and differences between *D. magna* and *D. pulex*.
- “Compared with the control daphnids, neonates exposed to the lowest concentration of nano-Ag had a normal appearance. However, approximately half of the neonates exposed to 0.1 mg/L of nano-Ag showed clear signs of the toxic effects, including significantly reduced body size, changes in feeding and/or metabolism assessed by lack or reduced amounts of algae (green colour) in the digestive tract (66.7% of examined neonates), lack or reduced amounts of the brown lipid storage droplets usually surrounding the intestine (53.3% of examined neonates), and lesions (40% of examined neonates). *D. magna* exposed to micro-Ag up to 1 mg/L, while significantly reduced in size (1 mg/L only), did not show abnormalities in the contents of their intestine, the amount of lipid storage droplets, or lesions, even at 1 mg/L (Fig. 3C).”
- There was consistent but low mortality throughout the 21-day exposures, with mortality rates ranging from 0 – 30% across the dose range. There was a lack of dose-dependency, with the 30% mortality rate occurring at the low dose (0.001 mg/L).
- A study by Li et al (2010) compared the acute aquatic toxicities (48-hour LC_50_s) of Ag, Au, and Ag-Au bimetallic nanoparticles to Daphnia magna. 1% sodium citrate was added to obtain silver nanoparticles with different molar ratios of silver ion to citrate (i.e. Ag:citrate ratios of 1:1.6, 1:3.1, or 1:4.2). As the ratio of Ag:citrate was decreased from 1:1.6 to 1:4.2, the average particle size increased from 36 to 66 nm. The Ag samples were composed of a mixture of spherical, prismatic, and rod shaped colloids (the rod particles were present at <1% of the total colloidal particles). Significant aggregation was detected in the AgNP solutions after 24-hour exposure to fresh water, suggesting that *daphnia* were subjected to a dynamic suspension of particles, originally containing small NPs (ranging from 36 – 66 nm) but undergoing significant aggregation over the exposure period (ranging from 378 – 553 nm after 24 h in fresh water). Testing for daphnia toxicity was performed in agreement with OECD guidelines (*Daphnia* Acute Immobilization Test and Reproduction Test). The 48-hour LC50 in *Daphnia* for silver ion was determined to be 0.002 mg/L. For AgNPs produced using different ratios of silver ion to citrate, the toxicity decreased with increasing amounts of citrate (LC50 = 5 – 10 µg/L), which corresponded to an increase in particle size, but when dissolved silver loss was taken into account (due to silver plating onto the reaction vessel), the toxicities were similar (LC50 = 3 – 4 µg/L). The authors speculated that the rapid aggregation of particles contributed to the lack of correlation between toxicity and apparent particle size. The LC50 value of gold NPs was found to be approximately 1000x greater (less toxic) than silver NPs. Over 95% of the Ag present was in solid form and not the ionic form, suggesting that the active component was the reduced form of the silver and not the presence of excess dissolved Ag ion. The toxicity effects of Ag-Au bimetallic NPs were in between AgNPs and AuNPs, but much closer to that of AgNPs (Li et al., 2010).
- Nanosilver as a powder (20-30nm) was suspended in a solution with 0.5% sodium citrate as stabilizer. The short-term (48 hour) LC50 in *Danio rerio* (fish) under static conditions was 7.07 mg Ag/L for adults and 7.20 mg Ag/L for juveniles; the short-term (48 hour) LC50 for Daphnia pulex (invertebrates) was 0.04 mg Ag/L for adults; and the short-term (48 hour) LC50 for *Ceriodaphnia dubia* was 0.067 mg Ag/L for juveniles. The short-term (96-hour) growth inhibition EC50 to *Pseudokirchneriela subcapitata* was 0.19 mg Ag/L. The test guidelines were ASTM 2002 (Griffitt et al., 2008, as cited in RIVM, 2009).
- *Daphnia magna* and *Pimephales promelas* (Fathead minnows) were exposed to ionic silver (silver nitrate ), and treated and untreated spherical uncapped nanosilver nanoparticles. Untreated nanosilver had average particle diameters of 10, 20, 30 and 50 nm, and was present in aqueous suspensions in 2mM phosphate buffer. Treated nanosilver suspensions (10 nm only) were run through a cation exchange resin to reduce the concentration of dissolved Ag^+^. The 48 hr EC50 for *Daphnia magna* for untreated nanosilver ranged from 0.00431 to 0.03036 mg/L, with increasing toxicity associated with the smallest particle sizes. The toxicity of 10 nm nanosilver preparations were similar, regardless of whether cation exchange was applied. The 48 hr EC50’s were 0.00215 and 0.00279 mg/L, respectively. The 96 hr LC50 and 7-d EC20 (based on larval survival and growth) for *P. promelas* were 0.0894 and 0.0461 mg/L, respectively for 10 nm untreated nanosilver, and 0.0047 and 0.00137 mg/L for silver nitrate. Testing in *D. magna* was conducted to ASTM Method E729, and testing in *P. promelas* was conducted to EPA/600/4-90/027F. Authors noted all sizes of nanosilver were less toxic on a mass basis compared to ionic silver, and although mass-based LC50’s for the different nanoparticles sizes differed, normalizing exposure to theoretical surface area largely eliminated differences among the exposure-response curves (Hoheisel et al., 2012).
- *Daphnia magna* were exposed for 24 h to suspensions of nanosilver (<100 nm and <150 nm), nano-copper dioxide (<50 nm), or solutions of ionic silver (as AgNO3) and ionic copper (as CuCl_2_^.^ 2H_2_O) in moderately hard reconstituted water, with the intent of comparing toxicity relative to changes in particle size and dispersion. Variables for the methods of preparation included stirring (24h), sonication (1h or 2h), and filtration (0.05, 0.1, and 0.45 μm membrane filters). Ag and Cu concentrations were measured by ICP-OES and ICP-MS. Zeta potential was measured as an indicator for the degree of dispersion. After stirring and sonication, all nano-Ag and nano-CuO preparations , including those that were filtered and unfiltered, had average particles sizes significantly greater (147.7 – 3,411 nm) than their nominal sizes of 100 nm, and 50 nm, respectively. Filtered suspensions as expected had lower average particles sizes (147.7 - 786 nm) and also greater zeta potential (-28.3 to - 34.6 mV), indicating greater dispersion. Mass based 24hr EC50’s in daphnia for filtered suspensions, with nominal particle sizes of 100 nm, and subjected to sonication, ranged from 0.009-0.0143 mg/L; for nominal particle sizes of 150 nm, and subjected to sonication the 24hr EC50 was 0.0042 mg/L; unfiltered suspensions with average nominal particle size <100 nm and < 150 nm had 24hr EC50’s in the range of 0.535-3.844 mg/L. Comparatively, 24hr EC50’s based on dissolved concentrations were very similar regardless of the nominal concentration or method of dispersion – all nanoAg solutions had normalized EC50’s in the range of 0.003-0.005 mg/L. A similar pattern was seen with the CuO solutions where filtered samples had the smallest average particle size and were the most toxic. Authors concluded the dissolved fraction had a greater influence on toxicity than the total mass, and noted the dissolution rate is largely affected by water quality parameters such as pH, hardness and dissolved organic matter (Jo et al., 2012).
- The algae *Chlamydomonas reinhardtii* was exposed to carbonate coated silver nanoparticles. The particle size ranged from 10 – 200 nm with a median particle diameter of 40 nm. Under experimental conditions, there was no aggregation and the Zeta potential was -3.66 mV. Decreased pH of the AgNP suspension resulted in less negative Zeta potentials and increased aggregation. Using an Ag-ISE based process, it was estimated that 1% of the Ag was in the form of free Ag^+^. Algae were exposed to AgNO3, AgNO3 + cysteine, AgNP, AgNP as a function of free Ag+ at the beginning of the experiment, and AgNP + cysteine. Algae were exposed from 1 – 5 hours at varying concentrations. The EC50s for reduced photosynthetic yield were as follows:

AgNO_3_ (1 – 2 hours) = 184 – 188 nM

AgNP (1 – 5 hours) = 829 – 3300 nM

AgNP (as free Ag^+^, 1- 5 hours) = 8 – 33 nM

AgNO_3_ showed similar EC50 values upon 1 or 2 hours of exposure, while AgNP toxicity was time-dependent for the first two hours, but stabilized after 2 hours. Based on total Ag concentration, AgNO_3_ displayed higher toxicity than AgNP, although this difference declined over time. However, based on Ag^+^, AgNP appeared to be more toxic than AgNO3. The authors suggested that this supports the hypothesis that while AgNP toxicity is related to Ag^+^ formation, it cannot be explained solely by the concentration of Ag^+^ ions in the original suspensions. The authors suggest that an additional interaction between the algae and the AgNPs may be forming additional Ag^+^.

To examine the effects of Ag+ to toxicity, cysteine was added as a silver ligand at concentrations from 1 to 500 nM in 5 or 10 µM AgNP. At the highest cysteine concentration, photosynthetic yield was comparable to control values. This suggests that the protective effects of cysteine are due to complexation with Ag^+^ in the exposure media resulting in reduced silver bioavailability. The authors interpreted these results as demonstrating the determinant role of free Ag^+^ for the toxicity of AgNP (Navarro et al., 2008). Not a guideline method, not comparable in exposure duration, etc. – cannot be compared to the GreenScreen™ criteria, informative only.

ENRHES conclusions:

LC50s ranged from 0.04 mg/L in *D. pulex* to 7.2 mg/L in juvenile *D. rerio*.

LC50’s ranged from 7.07 for adult *D. rerio* and 7.2 mg/L for juvenile *D. rerio*.

LC50’s ranged from 0.04 – 0.067 mg/L in *D. pulex* adults and *C. dubia* neonates, respectively.

EC50 in green algae *P. subcapitata* = 0.19 mg/L

**Summary of Invertebrates** **EC50’s:**

| Species | AgNP form | 48 hr EC50 | Reference |
| --- | --- | --- | --- |
| *Ceriodaphnia dubia* | Powder AgNP 60-100 nm | 0.00075 mg/L total  (0.00037 mg/L dissolved) | Lee et al., 2012 |
|  | Citrate-stabilized AgNP (average 13.3 nm) | 0.00798 mg/L total  (0.00088 mg/L dissolved) |  |
| *Ceriodaphnia dubia* | Powder AgNP 395 nm | 0.482 mg/L (in moderately hard water, the traditional growth medium)* | McLaughlin and Bonzongo, 2012 |
| *Daphnia magna* | Colloid 5-25 nm in water (nAg1) with 1% citrate capping agent | 0.004 mg/L | Asghari et al., 2012 |
|  | Colloid (average 16.6 nm) in water (nAg2) | 0.002 mg/L |  |
|  | Powdered dispersion 20 nm in deionized water (nAg3) | 0.187 mg/L (significant aggregation was observed) |  |
| *Daphnia magna* | Powder 60 nm | 1.0 mg/L | Kim et al., 2011b |
|  | Powder 300 nm | 1.4 mg/L |  |
| *Daphnia magna* | 35 nm (588 nm reconstituted) | 0.1 mg/L | Gaiser et al., 2011** |
| *Daphnia magna* | 36-66 nm in 1% sodium citrate (measured agglomeration to 378-553 nm after 24 h). | 0.002 mg/L | Li et al., 2010 |
| *Daphnia pulex* | Powder 20-30 nm, suspended in 0.5% sodium citrate solution | 0.04 mg/L adults | Griffitt et al., 2008 |
| *Ceriodaphnia dubia* |  | 0.067 mg/L juveniles |  |
| *Daphnia magna* | Nanosilver suspension in 2mM phosphate buffer, 10-50 nm | 0.00215 mg/L | Hoheisel et al., 2012 |

* not an OECD method

** study is of questionable validity

Using the most sensitive result, 0.00075 mg/L in *Ceriodaphnia dubia*, this would equate to GHS Category 1.

**Summary of LC50’s in Fish**

| Species | AgNP form | 96 hr LC50 (unless noted otherwise) | Reference |
| --- | --- | --- | --- |
| *Oryzias latipes* (Japanese Medaka) | Powder 60 nm | 28 mg/L | Kim et al., 2011b |
|  | Powder 300 nm | 67 mg/L |  |
| *Danio rerio* (zebrafish) | Powder 20-30 nm, suspended in 0.5% sodium citrate solution | 7.07 mg/L adults  7.20 mg/L juveniles | Griffitt et al., 2008 |
| *Pimephales promelas* (Fathead minnow) | Nanosilver suspension in 2mM phosphate buffer, 10-50 nm | 0.0894 mg/L | Hoheisel et al., 2012 |
| *Danio rerio* (zebrafish) | Polyvinylpyrolidone capped, 52-92nm | 48hr LC50 = 0.061 mg/L | Cunningham et al, 2013 |

Using the most sensitive result, 0.0894mg/L in *Pimephales promelas*, this would equate to GHS Category 1.

**Summary of EC50’s in Algae**

| Species | AgNP form | 96 hr EC50 | Reference |
| --- | --- | --- | --- |
| *P. subcapitata* | Powder AgNP 395 nm | 4.61 mg/L | McLaughlin and Bonzongo., 2012 |
| *Pseudokirchneriela subcapitata* | Powder 20-30 nm, suspended in 0.5% sodium citrate solution | 0.19 mg/L | Griffitt et al., 2008 |

Using the most sensitive result, 0.19 µg/L in *Pseudokirchneriela subcapitata*, this would equate to GHS Category 1.

**Chronic Aquatic Toxicity (CA) Score (vH, H, M or L): vH**

Nanosilver was assigned a score of **very High** concern (high confidence) for chronic aquatic toxicity based on a fish and invertebrate chronic studies that show LOEC of <0.1mg/L. This score is reported as high confidence as it is based on numerous well reported study data.

- Authoritative and Screening Lists
  - *Authoritative: Not on any authoritative lists*
  - *Screening:*
    - *New Zealand - GHS - 9.1A (algal) - Very ecotoxic in the aquatic environment*
    - *New Zealand - GHS - 9.1A (crustacean) - Very ecotoxic in the aquatic environment*
    - *New Zealand - GHS - 9.1A (fish) - Very ecotoxic in the aquatic environment*
- Chronic (21 d) and long-term effects of nanosilver (primary size 15 nm) on five successive generations of three Daphnia species (D. magna, D. pulex, and D. galeata) were investigated. Nanosilver dispersions were obtained by preparing a stock dispersion of NM-300. Chronic exposure provided EC10 values of 0.92 µg Ag L(-1) for D. magna showing the most sensitive chronic reaction and 2.25 and 3.45 µg Ag L(-1) for D. pulex and D. galeata, respectively. The multi-generation experiments resulted in effects on the population level for all tested species. Exposure of D. magna indicated an increased toxicity of nanosilver in the fifth generation of animals exposed to 10 µg Ag L(-1). Neonates from pre-exposed parental daphnids did not completely recover when transferred into clean water. Exposure of D. pulex and D. galeata revealed not only increasing toxicity in some generations, but also greater tolerance to nanosilver (Volker 2013).
- This is a non-GLP, chronic toxicity study on the early life stage of the fish Danio rerio following OECD guideline 210. Fish were exposed to concentrations of nanoparticulate silver in large static-renewal systems in which the test dispersion was permanently mixed by pumps. Range finding and orientation studies were conducted followed by two definitive tests, the first of which may run the risk of a bias by uneven conditions so has been statistically compared and combined where they do not differ significantly from the second test. A NOEC of 5.9 μg/L of nanosilver was determined in the valid 2nd test, based on an effect on growth, measured as total lengths (2013 reported in ECHA Registered Substances for Silver)
- The toxicity of silver nanoparticles to the development and survival of fish embryos was determined in a non-GLP compliant, non-guideline, published report (Kwok 2012), which follows sound scientific method, though there are some limitations in design and/or reporting. Fish embryos were exposed to four concentrations of each type of silver nanoparticle (large and small PVP coated, citrate coated and gum arabic coated), along with water and coating controls and a supernatant control for the large PVP coated treatment. The raw large PVP-coated nanoparticles were characterised as 75 ± 21 nm and the small as 21 ± 7 nm. Both were characterised as ~50 nm after 24 hours and ≥ 1 µm at 48 hours. Citrate coated nanoparticles were characterised as 7 ± 11 nm (raw) and 13 - 30 nm in test media. The gum arabic coated nanoparticles were characterised as 6 ± 2 nm core size (raw) and 70 nm in test media. The study is considered reliable and suitable for use for this endpoint. The 21 day NOEC to fish embryos exposed to silver nanoparticles is 150 µg/L for large PVP, 1500 µg/L for small PVP, 150 µg/L for citrate coated and 500 µg/L for gum arabic coated (Kwok 2012).
- In a near-guideline study, published in peer reviewed literature and considered suitable for use as a supporting study for this endpoint. The freshwater 21 -d NOEC value based on growth for Daphnia magna when exposed to carbonate coated silver nanoparticles was 2.5 µg/L (Zhao 2011).
- In the Gaiser et al. (2011) study summarized above (see acute aquatic toxicity section), a 21d NOEC in *Daphnia magna* was not determined. Authors noted the lowest dose (0.001 mg/L) had the highest mortality (30%) and speculated it was due to the rapid agglomeration at higher doses.
- Although no chronic aquatic toxicity data was identified for silver nanoparticles, RIVM (2009) extrapolated from Griffitt et al. (2008), using a 1000-fold uncertainty factor, to predict a PNEC of 0.00004 mg/L in *Daphnia pulex* (RIVM, 2009).
- As described above, 7-d EC20 (based on larval survival and growth) for *P. promelas* was 0.0461 mg/L, for 10 nm untreated nanosilver, and 0.00137 mg/L for silver nitrate per EPA/600/4-90/027F (Hoheisel et al., 2012).
- Adult Mozambique tilapia (O. mossambicus) were exposed to an aqueous suspension of silver nanoparticles (60-80 nm) in trisodium citrate and tannic acid for 8 days, per a non-guideline method. The LC50 was reported at 12.6 mg/L (Govindasamy and Rahuman, 2012)

**Environmental Fate (Fate)**

**Persistence (P) Score (vH, H, M, L, or vL):** *vH*

Nanosilver was assigned a score of very High concern (low confidence) for persistence. It is expected to be persistent because it is an element which cannot be degraded. The hazard score is based on professional judgement and therefore is reported as low confidence.

- Authoritative and Screening Lists
  - *Authoritative: Not on any authoritative lists*
  - *Screening:*
    - *EC - CEPA DSL - Persistent*

It may be noted that nanosilver particles are expected to aggregate, and may also bind to complexing and sorbing agents present in the environment (e.g. sediment, soil, and dissolved organic carbon). Factors affecting rates of aggregation and dissolution have been investigated, as reviewed in Kent and Vikeland, 2011.

**Bioaccumulation (B) Score (vH, H, M, L, or vL): L**

Nanosilver was assigned a score of **Low** concern (high confidence) for bioaccumulation based reported BCF values in non-mammalian studies. The score is based on study data and therefore is reported in high confidence.

- Authoritative and Screening Lists
  - *Authoritative: Not on any authoritative lists*
  - *Screening: Not on any screening lists*
- In a non-GLP, non-guideline study in which juvenile fish were exposed to dispersed (1:10) nanoparticulate silver NM-300 K and the total silver residues in different tissues were related to the water concentrations. The fish tissue portions were split into 1) head, gills and skin, 2) intestines (stomach and guts) and 3) fillet and rest (inner fish - organs and bones). The calculated BCFs for fish juvenile fish exposed to silver for 21 days ranged from 6.17 L/kg (inner fish exposed at 100 µg/L) to 326.99 L/kg (stomach and guts exposed at 25 µg/L) (2013 reported in ECHA Registered Substances for Silver).
- The uptake and effect of silver (as lactate Ag NPs of 40 nm) at low concentrations (10 µg/L) was investigated in the endobenthic bivalve *Scrobicularia plana* exposed, for 14 days, directly (water) or via the diet (contaminated diatoms, Nitzschia sp.) for 1 hour. Bioaccumulation of Ag in bivalves was measured by electrothermal atomic absorption spectrometry. Behavioural and biochemical biomarkers were determined in bivalves. There was no significant difference between bioaccumulation in AgNP exposure (753 ± 467 ng/g ww) and control. There was no significant difference between soft tissue concentrations of silver after dietary exposure to AgNP. Waterbourne exposure to silver did not affect feeding rate. Dietery exposure to AgNP resulted in reduced feeding rate after 10 days exposure (2013; reported in ECHA Registered Substances for silver).
- In a non-GLP, non-guideline study following standard scientific methods with 32 days exposure (0-28 days with silver) of adult zebra fish (*Danio rerio*) to 4 AgNP exposures (5, 15, 25 and 50 µg/L, commerically supplied material, resuspended in sodium citrate) and 5 µg/L Ag+ exposure. BCF was calculated from the concentration in water and the concentration in fish after 28 days exposure. The BCF for ionic silver was 95.5. Silver accumulated in both eviscerated caracass and in gills. Accumulation was an order of magnitude higher in gills than in carcass tissue at all time points.(2013; reported in ECHA Registered Substances for silver)
- Groups of 10 earthworms (*Eisenia fetida*) were exposed to neutron-activated silver nanoparticles (face-centered cubic structure, surface area 18.4 ± 0.4 m^2^/g, zeta potential -21.1 ± 0.4 mV, mean particle diameter 20.2 ± 2.5 nm, neutron activity 3.57 x 10^5^), silver ions, cobalt nanoparticles, or cobalt ions, for 28 days in the diet, and 6 earthworms served as controls. The earthworms were evaluated for the uptake, excretion, and biodistribution. The specific activity was used to calculate the actual concentration of engineered nanoparticles or ions in the samples (horse manure/food, earth-worm). Bioavailability was determined based on sequential extractions using the water to represent the water soluble metal species. 93% of silver nanoparticles were excreted within 48 hours. On day 30 (representing empty gut), using the mobile fraction, the BAF for nanosilver was calculated at 0.31 ± 0.12 (Coutris et al., 2012).
- Khan et al. (2012) have investigated the bioaccumlation dynamics of the estuarine snail *Peringia ulvae* exposed to silver nanoparticles. Field collected estuarine snails were exposed to silver nanoparticles for 1 to 5 days and were depurated for 16 to 24 days, depending on the experiments performed, in synthetic estuarine water. From the reported rate constant for metal uptake (Ku) and the rate constant for loss (Ke) for nanosilver a BCF of 0.0027 L/Kg was calculated following the dynamic BCF calculation reported in the REACH R.7C guidance.
- The fate and effects of three sizes of commercially available silver particles (including two nanoparticles: 10 and 35 nm) and ionic silver (as silver nitrate) was investigated using 10 day exposures with Oncorhynchus mykiss (rainbow trout). Uptake into the gills, liver and kidneys was quantified by inductively coupled plasma-optical emission spectrometry. Maximum tissue-specific BCF value for silver in gills was 6.9 and 35 in liver. Concentrations of silver in kidneys were below the analytical limit of detection, which precluded calculation of tissue-specific BCF values (2013; reported in ECHA Registered Substances for silver).
- Bioaccumlation dynamics of the freshwater snail Lymnaea stagnalis exposed to silver nanoparticles was investigated in aqueous and dietary experiments. Snails were exposed to silver nanoparticles for 2 -4 hours to1 day and were depurated for 48 hours to 25 days, depending on the experiments performed, in moderately hard water. From the reported rate constant for metal uptake (Ku) and the rate constant for loss (Ke) for nanosilver a BCF of 0.0060 L/Kg for cit-Ag NPS ans a BCF of 0.023 L/kg for Ha-Ag NPs were calculated following the dynamic BCF calculation reported in the REACH R.7C guidance (Croteau 2011).
- As summarized above, a Comet assay and analysis for bioaccumulation was performed in the polychaete, *Nereis diversicolor.* The worms were exposed to nominal concentrations of nanosilver (<100 nm, 99.5% metals basis, coated by 0.2 wt% PVP), micro-silver (2-3.5 µm, ≥99.9% trace metal analysis), and ionic silver (AgNO3) at 0, 1, 5, 10, 25, and 50 µg Ag/g dry weight sediment for 10 days. The presence of highly crystalline material was observed in nanosilver, suggesting the presence of large silver particles (aggregates, 20-200 nm, average 162 nm). For macrosilver, 5-10% of non-crystalline material was observed, suggesting it was not as pure as described by the manufacturer (i.e. <99.9% purity), and had both micro and nano-sized particles (8nm – 3 µm). Reported silver body burdens for the nano-, micron-, and ionic-silver treatments were 8.56, 6.92, and 9.86 µg/g dw, respectively. These values correspond to BAF factors of 0.17, 0.14, and 0.20, respectively (Cong et al., 2011).
- As summarized above, groups of 10 earthworms (Eisenia fetida) were exposed to nanosilver powder (30-50nm) coated with either polyvinylpyrrolidone (PVP) or oleic acid (OA) (both >95% pure Ag) in artificial soil medium, or silver nitrate, per a standard OECD sub-chronic reproduction toxicity test (the specified method is not cited but is reasonably documented and references OECD 2004). The worms were also evaluated for bioaccumulation. NPs were first suspended in deionized water and sonicated, and applied as suspensions to the soils at a rate of 50% of the moisture holding capacity (approximately 26% v/w). Nominal concentrations were 10, 100 and 1000 mg Ag/kg dry soil. AgNO3 was dissolved and added to soil medium at 10 and 100 mg Ag/kg soil. PVP coated particles were analyzed in solution with mean diameter of 56.35 nm, and OA coated particles had a mean diameter of 50.60 nm. OA particles also had greater percentage of aggregates in suspension as large as 200 nm. Zeta potentials were estimated at -35.9 mV for PVP-coated particles, and -45.4 mV for OA-coated particles. No metallic impurities were detected in the stock suspensions. The coatings accounted for 6.4 and 1.5 wt % of the mass of PVP and OA powders, respectively. Tissue concentrations were dose-dependent, however all BAF’s were below 0.1. (Shoults-Wilson et al., 2011).

**Physical Hazards (Physical)**

**Reactivity (Rx) Score (vH, H, M or L): L**

Nanosilver was assigned a score of Low concern (low confidence) for reactivity based on MSDS data. The low confidence is based on the lack of any reactivity test data specific to nanosilver.

- Authoritative and Screening Lists
  - *Authoritative: Not on any authoritative lists*
  - *Screening: Not on any screening lists*
- MSDS info for QSI-Nano® silver (unfunctionalized powder, 20-40nm): HMIS reactivity rating = 1 (QuantumSphere, Inc., 2007). This rating translates to “Slight hazard – materials which are normally stable, but can become unstable at high temperatures and pressures”.
- Mechanical impact on powders may result in explosion, although bulk silver is not explosive (RIVM, 2009- no further information provided). It would be inappropriate to assume nanosilver would have the same explosivity characteristics as silver in a bulk form. While similar sensitivity and severity has been demonstrated for various forms of carbon (coal, flour, carbon nanotubes, and nanostructured carbon blacks), marked differences were observed with various forms of aluminum. Small oxide layers on the aluminum presumably make the nanopowders less explosive than the macropowders, whereas nanopowders which tended to agglomerate showed explosion violence at the same order of magnitude as the macropowders (Bouillard et al., 2008).

**Flammability (F) Score (vH, H, M or L): L**

Nanosilver was assigned a score of Low concern (low confidence) for flammability based on MSDS data. The low confidence is based on the lack of any flammability test data specific to nanosilver.

- Authoritative and Screening Lists
  - *Authoritative: Not on any authoritative lists*
  - *Screening: Not on any screening lists*
- MSDS info for QSI-Nano® silver (20-40nm): HMIS flammability rating = 0 (QuantumSphere, Inc., 2007).
- Silver powder (particle size was 90% > 0.5 µm) was determined to be nonflammable according to the EU Method A.10 (Flammability (Solids) (ECHA database, 2009). As with explosivity discussed above, it would be inappropriate to assume nanosilver would have the same flammability characteristics as silver in a bulk form. Carbon nanotubes which have a higher surface area per mass (m^2^/g), had higher onset temperatures of combustion compared to carbon black. While specific sizes were not provided, the approximate onset temperature range spanned 390°C - 510°C. Conversely, with aluminum nanoparticles, onset temperatures of combustion have been reported at much lower temperature, starting well below the melting point for bulk aluminum (Bouillard et al., 2008).

**References**

Ahamed, M., M. Karns, M. Goodson, J. Rowe, S.M. Hussain, J.J. Schlager, and Y. Hong, 2008. DNA damage response to different surface chemistry of silver nanoparticles in mammalian cells. Toxicol and Appl Pharm. 233:404-410.

Asare, N., C. Instanes, W.J. Sandberg, M. Refsnes, P. Schwarze, M. Kruszewski, and G. Brunborg, 2012. Cytotoxic and genotoxic effects of silver nanoparticles in testicular cells. Toxicol. 291(1-3): p. 65-72.

Asghari, S., S.A. Johari, J.H. Lee, Y.S. Kim, Y.B. Jeon, H.J. Choi, M.C. Moon, and I.J. Yu, 2012. Toxicity of various silver nanoparticles compared to silver ions in Daphnia magna. J Nanobiotechnol. 10:14 doi:10.1186/1477-3155-10-14.

AshaRani, P.V., G. L. K. Mun, M. P. Hande, and S. Valiyaveettil, 2009. Cytotoxicity and genotoxicity of silver nanoparticles in human cells. ACSNano. 3(2):279-290.

Bouillard, J., A. Crossley, J.M. Dien, P. Dobson, T. Klepping, and A. Vignes, 2008. Safety Parameter Characterisation Techniques for Nanoparticles, Dissemination report, DR-152-200802-2, Project ID: NMP2-CT-2005-515843 (http://www.nanosafe.org/home/liblocal/docs/Dissemination%20report/DR2_s.pdf).

Card, J.W. and B. A. Magnuson, 2010. A method to assess the quality of studies that examine the toxicity of engineered nanomaterials. Int’l Jnl Toxicol. 29(4):402-410.

Cong, Y., G.T. Banta, H. Selck, D. Berhanu, E. Valsami-Jones, and V.E. Forbes, 2011. Toxic effects and bioaccumulation of nano-, micron- and ionic-Ag in the polychaete, Nereis diversicolor. Aq Toxico. 105: 403-411.

Coutris, C., T. Hertel-Aas, E. Lapied, E.J. Joner, and D.H. Oughton, 2012. Bioavailability of cobalt and silver nanoparticles to the earthworm Eisenia fetida, Nanotoxicol. March 6(2): 186-195.

Cunningham, S., M.E. Brennan-Fournet, D. Ledwith, L. Byrnes, and L. Joshi. 2013. Effect of nanoparticles stabilization and physicochemical properties on exposure outcome: acute toxicity of silver nanoparticles preparations in Zebrafish (Danio rerio). Environ Sci Technol. 47: 3883-3892.

Demir, E., G. Vales, B. Kaya, A. Creus, and R. Marcos, 2011. Genotoxic analysis of silver nanoparticles in Drosophila. Nanotoxicol. September, 5(3):417-424 (doi:10.3109/17435390.2010.529176).

Dobrzyńska 2014. Genotoxicity of silver and titanium dioxide nanoparticles in bone marrow cells of rats in vivo. Toxicology 315: 86 - 91.

ECHA (European Chemicals Agency), 2009. Information on Chemicals: Registered chemicals. Search term: CAS #7440-22-4. At: http://echa.europa.eu/web/guest/information-on-chemicals/registered-substances, accessed October 24th, 2015.

Engineered Nanoparticles: Review of Health and Environmental Safety (ENRHES), 2009. http://ihcp.jrc.ec.europa.eu/whats-new/enhres-final-report.

English, J.C. 2012. ‘Nanosilver’ as used in textiles Phase I: Scoping Project. NSF International. Feb 23, 2012. 9126858-tr01.

FDA, 1999. Federal Register Notice on Over-the-Counter Drug Products Containing Colloidal Silver Ingredients or Silver Salts. 64(158): 44653-44658 (http://www.fda.gov/ohrms/dockets/98fr/081799a.txt).

Gaiser, B.K., et al., Effects of silver and cerium dioxide micro- and nano-sized particles on Daphnia magna, 2011. J Environ Monit. May 13(5):1227-35.

Georgia Institute of Technology (GIT), National Nanotechnology Infrastructure Network, 2006, (http://www.nnin.org/doc/NNIN-1025.pdf).

Ghaderi, S., Tabatabaei, S., Varzi, H., Rashno, M. 2015. Induced adverse effects of prenatal exposure to silver nanoparticles on neurobehavioral development of offspring of mice. The Journal of Toxicological SciencesVol. 40 (2015) No. 2 April p. 263-275

Ghosh, M., J. Manivannan, S. Sinha, A. Chakraborty, S.K. Mallick, M. Bandyopadhyay, and A. Mukherjee, 2012. In vitro and in vivo genotoxicity of silver nanoparticles. Mutat Res. 749: 60-69.

Govindasamy, R. and A.A. Rahuman, 2012. Histopathological studies and oxidative stress of synthesized silver nanoparticles in Mozambique tilapia (Oreochromis mossambicus). J Env Sci. 24(6): 1091-1098.

Griffitt R.J., J. Luo, J. Gao, J.C. Bonzongo, and D.S. Barber, 2008. Effects of particle composition and species on toxicity of metallic nanomaterials in aquatic organisms. Environ Toxicol Chem. 27: 1972-1978.

Hinther A, Vawda S, Skirrow RC, Veldhoen N, Collins P, Cullen JT, van Aggelen G, Helbing CC. 2010. Nanometals induce stress and alter thyroid hormone action in amphibia at or below North American water quality guidelines. Environ Sci Technol. 44(21):8314-8321 (Abstract only, http://pubs.acs.org/doi/abs/10.1021/es101902n).

Hoheisel, S.M., S. Diamond, and D. Mount, 2012. Comparison of nanosilver and ionic silver toxicity in Daphnia magna and Pimephales promelas. Environ Toxicol Chem. 31(11): 2557-2563.

Hong, J.S., S. Kim, S.H. Lee, E. Jo, B. Lee, J. Yoon, I.C. Eom, H.M. Kim, P. Kim, K. Choi, M.Y. Lee, Y.R. Seo, Y. Kim, Y. Lee, J. Choi, and K. Park, 2013. Combined repeated-dose toxicity study of silver nanoparticles with the reproduction/developmental toxicity screening test. Nanotoxicology; Early Online 1-14.

Ji., J.H., J.U. Yoon, J. D. Park, B.S. Choi, Y.H. Chung, I.H. Kwon, J.Jeong, B.S. Han, J.H. Shin, J.H. Sung, K.S. Song, and I.J. Yu, 2007. Twenty-eight day inhalation toxicity study of silver nanoparticles in Sprague-Dawley rats. Inhal Toxicol.19:857-871.

Jo, H.J., J.W. Choi, S.H. Lee, and S.W. Hong, 2012. Acute toxicity of Ag and CuO nanoparticles suspensions against Daphnia magna: the importance of their dissolved fraction varying with preparation methods. J Haz Mat. 227-228, Pages 301-308.

Khan, F.R., Misra, S., García-Alonso, J., Smith B.D., Strekopytov, S., Rainbow,P., Luoma, N., and Valsami-Jones, E. 2012. Bioaccumulation Dynamics and Modeling in an Estuarine Invertebrate Following Aqueous Exposure to Nanosized and Dissolved Silver. Environ. Sci. Technol., 2012, 46 (14), pp 7621–7628

Kent, RD and Vikesland, PJ. 2011. Controlled Evaluation of Silver Nanoparticle Dissolution using Atomic Force Microscopy. Environ Sci Technol. Just Accepted Manuscript • DOI: 10.1021/es203475a • Publication Date (Web): 15 Dec 2011 Downloaded from http://pubs.acs.org on December 29, 2011.

Kim, J.S., K.S. Song, J.H. Sung, H.R. Ryu, B.G. Choi, H.S. Cho, J.K. Lee and I.J. Yu, 2012. Genotoxicity, acute oral and dermal toxicity, eye and dermal irritation and corrosion and skin sensitization evaluation of silver nanoparticles. Nanotoxicol. Early Online, 1-8 (DOI: 10.3109/17435390.2012.676099).

Kim, H.R., M.J. Kim, S.Y. Lee, S.M. Oh, and K.H. Chung, 2011a. Genotoxic effects of silver nanoparticles stimulated by oxidative stress in human normal bronchial epithelial (BEAS-2B) cells. Mut Res. 726: 129-135.

Kim, J., S. Kim, and S. Lee, 2011b. Differentiation of the toxicities of silver nanoparticles and silver ions to the Japanese medaka (Oryzias latipes) and the cladoceran daphnia magna. Nanotoxicology, Vol. 5, No. 2 , Pages 208-214.

Kim, J.S., Sung, J.H., Ji, J.H., Song, K.S., Lee, J.H., Kang, C.S., Yu, I.J. 2011c. In vivo Genotoxicity of Silver Nanoparticles after 90-day Silver Nanoparticle Inhalation Exposure. Safety and Health at Work. Volume 2, Issue 1, March 2011, Pages 34–38

Kim, Y.S., M.Y. Song, J.D. Park, K.S. Song, H.R. Ryu, Y.H. Chung, H.K. Chang, J.H. Lee, K.H. Oh, B.J. Kelman, I.K. Hwang, and I.J. Yu, 2010. Subchronic oral toxicity of silver nanoparticles. Particle Fibre Toxicol. 7: 20.

Kim, Y.S., S.I. Yang, and J.C. Ryu, 2010a. Cytotoxicity and genotoxicity of nano-silver in mammalian cell lines. Mol Cell Toxicol. 6:119-125.

Kim Y.S., J.S. Kim, H.S. Cho, D.S. Rha, J.D. Park, B.S. Choi, R. Lim, H.K. Chang, Y.H. Chung, I.H. Kwon, J. Jeong, B.S. Han, and I.J. Yu, 2008. Twenty-eight-day oral toxicity, genotoxicity, and gender-related tissue distribution of silver nanoparticles in Sprague-Dawley rats. Inhal Toxicol. 20: 575-583.

Korani, M., S.M. Rezayat, K. Gilani, S. A. Bidgoli, and S. Adeli. Acute and subchronic dermal toxicity of nanosilver in guinea pig. Int J Nanomed. 6: 855-862.

Kwok, K., Auffan, M, Badireddy A.R, Nelson C,M, Wiesner M,R, Chilkoti A, Liu J, Marinakw S.M., Hinton D.E. 2012. Uptake of silver nanoparticles and toxicity to early life stages of Japanese medaka (Oryzias latipes): Effect of coating materials. Aquatic toxicology, vol. 120-121, pp. 59-66.

Lee, Y.J., J. Kim, J. Oh, S. Bae, S. Lee, I.S. Hong, and S.H. Kim, 2012. Ion-Release Kinetics and Ecotoxicity Effects of Silver Nanoparticles. Environ Toxicol Chem. 31(1): 155-159.

Lee Y, Choi J, Kim P, et al. 2012 b. A Transfer of Silver Nanoparticles from Pregnant Rat to Offspring. Toxicological Research. 2012;28(3):139-141. doi:10.5487/TR.2012.28.3.139.

Li 2013. Cytotoxicity and genotoxicity assessment of silver nanoparticles in mouse.

Li 2013b. Cytotoxic and genotoxic effects of silver nanoparticles on primary Syrian hamster embryo (SHE) cells. J. Nanosci. Nanotechnol. 13(1), 161 – 168.

Li, Y., D.H. Chen, J. Yan, Y. Chen, R.A. Mittelstaedt, Y. Zhang, AlS. Biris, R.H. Heflich, and T. Chen, 2012. Genotoxicity of silver nanoparticles evaluated using the Ames test and in vitro micronucleus assay. Mutat Res. 745:4-10.

Li, T., B. Albee, M. Alemayehu, R. Diza, L. Ingham, S. Kamal, M. Rodriguez, S.W. Bishnoi, 2010. Comparative toxicity study of Ag, Au, and Ag–Au bimetallic nanoparticles on Daphnia magna. Anal Bioanal Chem. 398(2): 689-700.

Locht, L.J., M.O. Pedersen, S. Markholt, B.M. Bibby, A. Larsen, M. Penkowa, M.Stoltenberg, and J. Rungby, 2011. Metallic silver fragments cause massive tissue loss in the mouse brain, in Basic Clin Pharmacol Toxicol. Jul 109(1):1-10.

Loeschner, K., N. Hadrup, K. Qvortrup, A. Larsen, X. Gao, U. Vogel, Al Mortensen, H.R. Lam and E.H. Larsen, 2011. Distribution of silver in rats following 28 days of repeated oral exposure to silver nanoparticles or silver acetate, Particle Fibre Toxicol. 8:18.

Mahabady MK. (2012) African Journal of Pharmacy and Pharmacology Vol. 6(6), 419-424, February 2012.

Maneewattanapinyo, P., W. Banlunara, C. Thammacharoen, S. Ekgasit, and T. Kaewamatawong, 2011. An evaluation of acute toxicity of colloidal silver nanoparticles. J Vet med Sci. 73(11): 1417-1423.

McLaughlin, J., and J.C.J. Bonzongo, 2012. Effects of Natural Water Chemistry on Nanosilver Behavior and Toxicity to Ceriodaphnia Dubia and Pseudokirchneriella Subcapitata, Environ Toxicol Chem. 31 (1):168-175.

Mei, N., Y. Zhang, Y. Chen, X. Guo, W. Ding, S.F. Ali, A.S. Biris, P. Rice, M.M. Moore, and T. Chen. 2012. Silver nanoparticle-induced mutations and oxidative stress in mouse lymphoma cells. Environ and Mol Mut. 53:409-419.

Navarro, E., F. Piccapietra, B. Wagner, F. Marconi, R. Kaege, N. Okzak, L. Sigg, and R. Behra, 2008. Toxicity of silver nanoparticles to Chlamydomonoas reinhardtii. Environ Sci Technol. 42(23) :8959-8964.

Nymark, P. et al. (2013). Genotoxicity of polyvinylpyrrolidone-coated silver nanoparticles in BEAS 2B cells. Toxicology 313: 38 - 48.

Park, E.J., E. Bae, J. Yi, Y. Kim, K. Choi, S.H. Lee, J. Yoon, B.C. Lee, and K. Park, 2010. Repeated-dose toxicity and inflammatory responses in mice by oral administration of silver nanoparticles. Environ Toxicol and Pharmacol. 30: 162-168.

Philbrook, N.A., L.M. Winn, A.R.M. Nabiul Afrooz, N.B. Saleh, and V.K. Walker, 2011. The effect of TiO2 and Ag nanoparticles on reproduction and development of Drosophila melanogaster and CD-1 mice. Toxicol Appl Pharm. 257: 429-436.

QuantumSphere, Inc., MSDS for QSI-Nano Silver Posder, 05/24/07. Accessed 10/11/13, http://www.qsinano.com/pdf/MSDS_QSI_Nano_Silver_24_May_07.pdf.

RIVM, 2009. Nanomaterials under REACH, Nanosilver as a case study. Report 601780003, National Institute for Public Health and the Environment.

Sahu SC, Njoroge J, Bryce SM, Yourick JJ, Sprando RL. 2014. Comparative genotoxicity of nanosilver in human liver HepG2 and colon Caco2 cells evaluated by a flow cytometric in vitro micronucleus assay. J Appl Toxicol. 2014 Nov;34(11):1226-34.

Samberg, M.E., S.J. Oldenburg, and N.A. Monteiro-Riviere, 2010. Evaluation of Silver Nanoparticle Toxicity in Skin in vivo and Keratinocytes in vitro, Environ Health Pers. 118(3):407-413.

Shoults-Wilson, W.A., B.C. Reinsch, O.V. Tsyusko, P.M. Bertsch, G.V. Lowry, and J.M. Unrine, 2011. Effect of silver nanoparticle surface coating on bioaccumulation and reproductive toxicity in earthworms (Eisenia fetida). Nanotoxicol. September 5 (3): 432-444.

Skalska J., Frontczak-Baniewicz, M., Struz˙yn´ska, L. 2014. Synaptic degeneration in rat brain after prolonged oral exposure to silver nanoparticles. NeuroToxicology 46 (2015) 145–154.

Sohn, E.K Johari, S.A., Kim, T.G. et al., 2015. Aquatic Toxicity Comparison of Silver Nanoparticles and Silver Nanowires,” BioMed Research International, vol. 2015, Article ID 893049, 12 pages, 2015.

Song, K.S., J.H. Sung, J.H. Ji, J.H. Lee, J.S. Lee, H.R. Ryu, J.K. Lee, Y.H. Chung, H.M. Park, B.S. Shin, H.K. Chang, B. Kelman, and I.J. Yu, 2012. Recovery from silver-nanoparticle-exposure-induced lung inflammation and lung function changes in Sprague Dawley rats. Nanotoxicol. Early Online 1-12 (COI: 10.3109/17435390.2011.648223)

Stebounova, L.V., A. Adamcakova-Dodd, J.S. Kim, H. Park, P.T. O’Shaughnessy, V.H. Gassian, and P.S. Thorne, 2011. Nanosilver induces minimal lung toxicity or inflammation in a subacute murine inhalation model, Particle Fibre Toxicol. 8:5.

Sung, J.H., J.H. Ji, K.S. Song, J.H. Lee, K.H. Choi, S.H. Lee and I.J. Yu, 2011. Acute inhalation toxicity of silver nanoparticles, Toxicol Indust Health. 27(2): 149-154.

Sung. J.H., J.H. Ji, J.D. Park, J.U. Yoon, D.S. Kim, K.S. Jeon, M.Y. Song, J. Jeong, B.S. Han, J.H. Han, Y.H. Chung, H.K. Chang, J.H. Lee, M.H. Cho, B.J. Kelman, and I.J. Yu, 2009. Subchronic inhalation toxicity of silver nanoparticles. Toxicol Sci. 108(2):452-461.

Sung, J.H., J.H. Ji, J.U. Yoon, D.S. Kim, M.Y. Song, J.Jeong, B.S. Han, J.H. Han, Y.H. Chung, J. Kim, T.S. Kim, H.K. Chang, E.J. Lee, J.H. Lee, and I.J. Yu, 2008. Lung Function Changes in Sprague-Dawley Rats After Prolonged Inhalation Exposure to Silver Nanoparticles. Inhal Toxicol. 20(6): 567-574.

Völker C, Boedicker C, Daubenthaler J, Oetken M, Oehlmann J. Comparative Toxicity Assessment of Nanosilver on Three Daphnia Species in Acute, Chronic and Multi-Generation Experiments. Shankar SS, ed. PLoS ONE. 2013;8(10):e75026. doi:10.1371/journal.pone.0075026.

Wise, J.P., Sr., B.C. Goodale, S.S. Wise, G.A. Craig, A.F. Pongan, R.B. Walter, W.D. Thompson, A. Ng, A.Abouesissa, H. Mitani, M.J. Spalding, and M.D. Mason, 2010. Silver nanospheres are cytotoxic and genotoxic to fish cells. Aquat Toxicol. 97(1): 34-41.

Xu, L; Shao, A; Zhao, Y; Wang, Z; Zhang, C; Sun, Y; Deng, J; Chou, L 2015. Neurotoxicity of Silver Nanoparticles in Rat Brain After Intragastric Exposure. Journal of Nanoscience and Nanotechnology, Volume 15, Number 6, June 2015, pp. 4215-4223(9).

Yin. N., Yao, X., Zhou, Q., Faiola, F. 2015. Vitamin E attenuates silver nanoparticle-induced effects on body weight and neurotoxicity in rats. Biochemical and Biophysical Research Communications. Volume 458, Issue 2, 6 March 2015, Pages 405–410.

Zhao, C.M. and W.X. Wang, 2011. Comparison of acute and chronic toxicity of silver nanoparticles and sivler nitrate to Daphnia magna. Environ Toxicol Chem. 30(4): 885-892.

# APPENDIX A: Hazard Benchmark Acronyms

**(alphabetical order)**

**(AA) Acute Aquatic Toxicity**

**(AT) Acute Mammalian Toxicity**

**(B) Bioaccumulation**

**(C) Carcinogenicity**

**(CA) Chronic Aquatic Toxicity**

**(Cr) Corrosion/ Irritation (Skin/ Eye)**

**(D) Developmental Toxicity**

**(E) Endocrine Activity**

**(F) Flammability**

**(IrE) Eye Irritation/Corrosivity**

**(IrS) Skin Irritation/Corrosivity**

**(M) Mutagenicity and Genotoxicity**

**(N) Neurotoxicity**

**(P) Persistence**

**(R) Reproductive Toxicity**

**(Rx) Reactivity**

**(SnS) Sensitization- Skin**

**(SnR) Sensitization- Respiratory**

**(ST) Systemic/Organ Toxicity**

**Appendix B**

**Optional Hazard Summary Table**

| **Route** | **GreenScreen™Hazard Ratings: Nanosilver, metallic** | | | | | | | | | | | | | | | | | | | |
| --- | --- | --- | --- | --- | --- | --- | --- | --- | --- | --- | --- | --- | --- | --- | --- | --- | --- | --- | --- | --- |
|  | **Group I Human** | | | | | **Group II and II* Human** | | | | | | | | | **Ecotox** | | **Fate** | | **Physical** | |
|  | **C** | **M** | **R** | **D** | **E** | **AT** | **ST** | | **N** | | **SnS** | **SnR** | **IrS** | **IrE** | **AA** | **CA** | **P** | **B** | **RX** | **F** |
|  |  |  |  |  |  |  | _Single_ | _Repeated*_ | _Single_ | _Repeated*_ |  |  |  |  |  |  |  |  |  |  |
| o | DG | *M* | *L* | *L* | DG | **L** | DG | **M** | DG | *M* | **L** | DG | **L** | **L** | **vH** | **vH** | *vH* | **L** | *L* | *L* |
| d | DG |  | DG | DG |  | **L** | DG | DG^^[[13]](#footnote-13)^^ | DG |  |  |  |  |  |  |  |  |  |  |  |
| i | DG |  | DG | DG |  | DG4 | DG | **H** | DG |  |  |  |  |  |  |  |  |  |  |  |

1. Use GreenScreen® Assessment Procedure (Guidance) V1.2 [↑](#footnote-ref-1)
2. See GreenScreen Guidance V1.2 [↑](#footnote-ref-2)
3. Note any composition or hazard attributes of the chemical product relevant to how it is manufactured. For example, certain synthetic pathways or processes result in typical contaminants, by-products or transformation products. Explain any differences between the manufactured chemical product and the GreenScreen assessment of the generic chemical by CAS #. [↑](#footnote-ref-3)
4. See Appendix A for a glossary of hazard endpoint acronyms [↑](#footnote-ref-4)
5. See Appendix B for alternative GreenScreen Hazard Summary Table (Classification presented by exposure route) [↑](#footnote-ref-5)
6. For inorganic chemicals only, see GreenScreen Guidance V1.2 Section 14.4. (Exceptions for Persistence) [↑](#footnote-ref-6)
7. For Systemic Toxicity and Neurotoxicity, repeated exposure data are preferred. Lack of single exposure data is not a Data Gap when repeated exposure data are available. In that case, lack of single exposure data may be represented as NA instead of DG. See GreenScreen Guidance V1.2 Section 9.3. [↑](#footnote-ref-7)
8. See GreenScreen Guidance V1.2 Section 13 [↑](#footnote-ref-8)
9. A moiety is a discrete chemical entity that is a constituent part or component of a substance. A moiety of concern is often the parent substance itself for organic compounds. For inorganic compounds, the moiety of concern is typically a dissociated component of the substance or a transformation product. [↑](#footnote-ref-9)
10. The CPA “Red List” refers to chemicals 1. flagged as Benchmark 1 using the GreenScreen™ List Translator or 2. flagged as Benchmark 1 or 2 using the GreenScreen™ List Translator and further assessed and assigned as Benchmark 1. The most recent version of the GreenScreen™ List Translator should be used. [↑](#footnote-ref-10)
11. The way you conduct assessments for transformation products depends on the Benchmark Score of the parent chemical (See Guidance). [↑](#footnote-ref-11)
12. Ideally one would make mutagenic observations at concentrations below cytotoxicity. If high levels of cytotoxicity occur the confidence in the results decreases. [↑](#footnote-ref-12)
13. Data are available but not sufficient to assign a hazard rating. [↑](#footnote-ref-13)
