# Supplementary material for: Use of a modified GreenScreen tool to conduct a screening-level comparative hazard assessment of conventional silver and two forms of nanosilver
Source: Environ Health. 2016 Nov 8;15:105. doi: 10.1186/s12940-016-0188-y (PMC5101654; doi:10.1186/s12940-016-0188-y)
Supplement: Additional file 3: — GreenScreen for HeiQ AGS20 2015. (DOCX 93 kb) [file 12940_2016_188_MOESM3_ESM.docx]

**Chemical Hazard Assessment for *HeiQ AGS-20, a silver-silica nanocomposite containing silver nanoparticles imbedded in a matrix of amorphous silicon dioxide (CAS # N/A)***

**Modified from GreenScreen^®^ Version 1.2^[[1]](#footnote-1)^**

| - - 1. **Initial Modified GreenScreen^®^ Assessment Prepared By:** | **Initial Modified GreenScreen^®^ Assessment Quality Control Performed By:** |
| --- | --- |
| Name: Nancy Linde, Toxicologist  Brad Lampe, Toxicologist  Teresa McGrath, Supervising Toxicologist | Name: Caroline English, Ph.D., D.A.B.T. |
| Title: Senior Product Compliance Engineer | Title: Senior Toxicologist |
| Organization: NSF International | Organization: NSF International |
| Date: July 30, 2012  Revised: January 22, 2013 | Date: July 30, 2012  Revised: January 22, 2013 |
|  | **Updated by**: Eric Rosenblum, Ph.D., D.A.B.T.  Organization: Rosenblum Environmental LLC  Date: October 31, 2015 |

**Confirm application of the *Disclosure and Assessment Rules and Best Practice*^[[2]](#footnote-2)^:** (List disclosure threshold and any deviations)

*Yes, this GreenScreen™ review was performed on 100% AGS-20*

**Chemical Name (CAS #):** AGS-20 (CAS # N/A) is a silver-silica nanocomposite containing silver nanoparticles imbedded in a matrix of amorphous silicon dioxide. The silver metal content is 19.3%. It is manufactured by HeiQ Materials AG, Switzerland (EPA, 2009c).

**Also Called:**

N/A

**Suitable analogs or moieties of chemicals used in this assessment (CAS #’s):**

None

Note OPPT reported several silver-based nanomaterials as analogs for fulfilling data requirements for subchronic toxicity, reproductive and developmental toxicity, or genetic toxicity. Those analogs were pure nanosilver particles (18-19 nm) (Sung et al., 2009), pure nanosilver particles (60 nm) (Kim et al., 2008), nanosilver (size not disclosed) (Larese et al., 2009), and nano-silver coated wound dressing (no further info on size or auxiliary ingredients was reported) (Trop, 2006 and Viachou et al., 2007) (EPA 2010b). Transformation products of AGS-20 are unknown.

Based on antimicrobial properties of the product, release of Ag+ from AGS-20 can be expected, and anticipated transformation products include silver salts formed by precipitation of Ag+ in the environment. EPA anticipates that humans could also be exposed to silver nanoparticles that break away from the AGS-20 nanosilver-silica composite particles. These non-composite nanosilver products in textiles (nanoscale Ag0 and AgCl) have been assessed in a separate GreenScreen™ (Nanosilver, metallic CAS 7440-22-4). The current GreenScreen™ therefore addresses specifically the silicon-silver nanocomposite AGS-20.

**Chemical Structure(s):**

Not applicable – composite of silver nanoparticles imbedded in a matrix of amorphous silicon dioxide (SiO2).

**Notes related to production specific attributes^[[3]](#footnote-3)^:**

1. Particle size (e.g. silica of respirable size) – AGS-20 contains silver nanoparticles with a typical diameter of 1-10 nm, and the silver-silica nanocomposite is an aggregate of mixed particles with an average diameter of approximately 1 micron (Egger et al., 2009).

2. Structure (e.g. amorphous vs. crystalline) – the silicon dioxide component is amorphous (Egger et al., 2009).

3. Mobility (e.g. Water solubility, volatility) – water solubility of AGS-20 was reported at 210 mg/L at 20 °C (EPA, 2009a).

4. Bioavailability – as stated above, the silica reduces the mobility of the silver and limits the potential for release of the nanoparticles (Egger et al., 2009). (For AGS-20, released silver particles, or released silver ions)

In addition to the traditional information gathered on inorganic materials, the following data quality parameters specific to nanomaterials (Card and Magnuson, 2010) were examined for AGS-20:

5. Agglomeration and/or aggregation – see particle size discussion above.

6. Purity - not available

7. Shape - spherical

8. Surface area – not available

9. Surface charge - not available

10. Surface chemistry (including composition and reactivity) ––transmission electron micrograph images show the silver particles are dispersed throughout the silica (which prevents agglomeration of the silver particles), furthermore the silica reduces the mobility of the silver and limits the potential for release of the nanoparticles (Egger et al., 2009).

11. Whether any characterization was conducted in the relevant experimental media – not available

**Identify Applications/Functional Uses:**

**(e.g., Cleaning product, TV casing)**

1. preservative in textiles

**GreenScreen Benchmark Score and Hazard Summary Table:^[[4]](#footnote-4),^^[[5]](#footnote-5),^^[[6]](#footnote-6),^^[[7]](#footnote-7)^** Numerous data gaps exist for AGS-20, which earns a BM score of U (Unspecified).

Note: Hazard levels (Very High (vH), High (H), Moderate (M), Low (L), Very Low (vL)) in *italics* reflect estimated values, authoritative B lists, screening lists, weak analogues, and lower confidence. Hazard levels in **BOLD** font are used with good quality data, authoritative A lists, or strong analogues. Group II Human Health endpoints differ from Group II* Human Health endpoints in that they have four hazard scores (i.e., vH, H, M and L) instead of three (i.e., H, M and L), and are based on single exposures instead of repeated exposures.

**Environmental Transformation Products and Ratings^[[8]](#footnote-8)^:**

**Identify feasible and relevant environmental transformation products** **(i.e., dissociation products, transformation products, valence states)** **and/or moieties of concern**^[[9]](#footnote-9)^

| **Life Cycle Stage** | **Transformation Pathway** | **Transformation Products** | **CAS #** | **On CPA Red List^^[[10]](#footnote-10)^^?** | **GreenScreen™Rating^^[[11]](#footnote-11)^^** |
| --- | --- | --- | --- | --- | --- |
| Textile Production (masterbatch, fiber and fabric preparation, fabric padding process) | Degradation or dissociation followed by agglomeration | Pure Ag^0^ aggregates | 7440-22-4 | Yes | 1c (See note below) |
| Consumer use (release from textiles during wear and washing) |  |  |  |  |  |
| End of life (release, dissolution, aggregation, precipitation) |  |  |  |  |  |
| Consumer use (release from textiles during wear and washing) | In the presence of hypochlorite, elemental silver may oxidize and convert into silver chloride (EPA, 2010a). | Silver chloride (AgCl) | 7783-90-6 | No |  |
| End of life (release, dissolution, aggregation, precipitation) |  |  |  |  |  |
| Consumer use where release from textiles occurs during wear and washing | Release / degradation / dissociation | Silver ion (Ag+) | 14701-21-4 | No |  |
| End of life (release, dissolution, aggregation, precipitation) |  |  |  |  |  |
| End of life (release, dissolution, aggregation, precipitation) | Release / dissolution / aggregation / precipitation | Silver sulfide (Ag_2_S) | 21548-73-2 | No |  |
| End of life (release, dissolution, aggregation, precipitation) | Release / dissolution / aggregation / precipitation | Silver thiosulfate (Ag_2_H_2_O_3_S_2_) | 23149-52-2 | No |  |

Note: For the above noted transformation products, silver (CAS# 7440-22-4) was listed on the Japanese NITE list as GHS Category 1 for respiratory effects – Specific target organ/systemic toxicity following single exposure. This translates to *vH* concern for a Group II Human endpoint, and may result in a **BM 1** score when coupled with **vH** persistence. It may be further noted the NITE listing is based on the ACGIH (2001) reports pertaining to pulmonary edema developed after exposure to heated metallic silver fumes, and ATSDR (1997) reported irritation from occupational exposure to dust.

OPPT states “the SAP concluded that the hazards of silver ions would be the same, whether they came from conventional silver or from nanosilver particles (EPA 2010b).

**Introduction:**

See “‘Nanosilver’ as used in textiles Phase I: Scoping Project.” (English, 2012)

**Hazard Classification Summary Section:**

**For all hazard endpoints:**

- **Search all GreenScreen specified lists. Report relevant results either in each hazard endpoint section or attach to the end of the report.**
- **Always indicate if suitable analogs or models were used.**
- **Attach modeling results (See Appendix C).**
- **Include all references either in each hazard endpoint section or at the end of the report.**

**Group I Human Health Effects (Group I Human)** all Group 1 human health endpoints have no data and are assigned DG (data gap).

**Carcinogenicity (C) Score (H, M or L):** DG **(**No Data)

- Authoritative and Screening Lists
  - *Authoritative: Not present on any authoritative lists*
  - *Screening: Not present on any screening lists*

**Mutagenicity/Genotoxicity (M) Score (H, M or L):** DG (No Data)

Authoritative and Screening Lists

- - *Authoritative: Not on any authoritative lists*
  - *Screening: Not on any screening lists*

**Reproductive Toxicity (R) Score (H, M, or L):** DG (No Data)

- Authoritative and Screening Lists
  - *Authoritative: Not on any authoritative lists*
  - *Screening: Not on any screening lists*

**Developmental Toxicity incl. Developmental Neurotoxicity (D) Score (H, M or L):** DG (No Data)

- Authoritative and Screening Lists
  - *Authoritative: Not on any authoritative lists*
  - *Screening: Not on any screening lists*

**Endocrine Activity (E) Score (H, M or L):** DG (No Data)

- Authoritative and Screening Lists
  - *Authoritative: Not on any authoritative lists*
  - *Screening: Not on any screening lists*

**Group II and II* Human Health Effects (Group II and II* Human)**

*Note: Group II and Group II* endpoints are distinguished in the v 1.2 Benchmark system (the asterisk indicates repeated exposure). For Systemic Toxicity and Neurotoxicity, Group II and II* are considered sub-endpoints. When classifying hazard for Systemic Toxicity/Organ Effects and Neurotoxicity endpoints, repeated exposure results are required and preferred. Lacking repeated exposure results in a data gap. Lacking single exposure data does not result in a data gap when repeated exposure data are present (shade out the cell in the hazard table and make a note). If data are available for both single and repeated exposures, then the more conservative value is used.*

**Acute Mammalian Toxicity (AT) Group II Score (vH, H, M or L):** *M*

AGS-20 was assigned a score of **Moderate** for acute mammalian toxicity based on inhalation data. OPPT has evaluated acute oral, dermal and inhalation data for AGS-20 per FIFRA registration requirements. The public docket does not contain the full test reports, but the following conclusions reported below are published in the docket. The confidence level is reported as low based on poorly reported study data reported in a secondary source.

- Authoritative and Screening Lists
  - *Authoritative: Not on any authoritative lists*
  - *Screening: Not on any screening lists*

**Summary of available toxicity data for AGS- 20.**

| **Toxicity Endpoint** | **Reported LD/LC50** (EPA 2009b) | **OPPT assigned category for AGS-20** (EPA 2010b) | **OPPT category values** (http://www.epa.gov/oppfead1/labeling/lrm/chap-07.pdf). | **GreenScreen™rating** (H, M, L, or DG) |
| --- | --- | --- | --- | --- |
| **Acute oral** | >2,000 mg/kg | III | LD50 >500 – 5,000 mg/kg | L |
| **Acute Dermal** | >2,000 mg/kg | III | LD50 >2,000 – 5,000 mg/kg | L |
| **Acute Inhalation** | >1.081 mg/L (MMAD 1.42 um and 1.1 um) | III | 4 hr LC50 >0.5 – 2 mg/L | M |
| **Primary Eye Irritation** | No abnormal findings in treated eyes after 7 days | III | Corneal involvement or other eye irritation clearing in 7 days or less | M (equivalent to GHS 2B) |
| **Primary Skin Irritation** | Not irritating | IV | Mild or slight irritation at 72 hours (no irritation or slight erythema) | L |
| **Dermal Sensitization** | No sensitization was observed | Non-sensitizer | N/A | L |

**Systemic Toxicity/Organ Effects incl. Immunotoxicity (ST)**

**(ST-single) Group II Score (single dose: vH, H, M or L);** DG (no data)

- Authoritative and Screening Lists
  - *Authoritative: Not on any authoritative lists*
  - *Screening: Not on any screening lists*

**(ST-repeat) Group II* Score (repeated dose: H, M, L):** DG (no data)

- Authoritative and Screening Lists
  - *Authoritative: Not on any authoritative lists*
  - *Screening: Not on any screening lists*

**Neurotoxicity (N)**

**(N-single) Group II Score (single dose: vH, H, M or L)** DG (no data)

- Authoritative and Screening Lists
  - *Authoritative: Not on any authoritative lists*
  - *Screening: Not on any screening lists*

**(N-repeat) Group II* Score (repeated dose: H, M, L):** DG (no data)

- Authoritative and Screening Lists
  - *Authoritative: Not on any authoritative lists*
  - *Screening: Not on any screening lists*

**Skin Sensitization (SnS) Group II* Score (H, M or L):** *L*

AGS-20 was assigned a score of Low for skin sensitization (see Summary of available toxicity data for AGS- 20 within the acute toxicity section). The confidence level is reported as low based on poorly reported study data reported in a secondary source.

- Authoritative and Screening Lists
  - *Authoritative: Not on any authoritative lists*
  - *Screening: Not on any screening lists*

**Respiratory Sensitization (SnR) Group II* Score (H, M or L):** DG (no data)

- Authoritative and Screening Lists
  - *Authoritative: Not on any authoritative lists*
  - *Screening: Not on any screening lists*

**Skin Irritation/Corrosivity (IrS) Group II Score (vH, H, M or L):** *L*

AGS-20 was assigned a score of Low for Skin Irritation/Corrosivity (see Summary of available toxicity data for AGS- 20 within the acute toxicity section). The confidence level is reported as low based on poorly reported study data reported in a secondary source.

- Authoritative and Screening Lists
  - *Authoritative: Not on any authoritative lists*
  - *Screening: Not on any screening lists*

**Eye Irritation/Corrosivity (IrE) Group II Score (vH, H, M or L):** *M*

AGS-20 was assigned a score of Moderate for Eye Irritation/Corrosivity (see Summary of available toxicity data for AGS- 20 within the acute toxicity section). The confidence level is reported as low based on poorly reported study data reported in a secondary source.

- Authoritative and Screening Lists
  - *Authoritative: Not on any authoritative lists*
  - *Screening: Not on any screening lists*

**Ecotoxicity (Ecotox)**

**Acute Aquatic Toxicity (AA) Score (vH, H, M or L):** DG (no data)

- Authoritative and Screening Lists
  - *Authoritative: Not on any authoritative lists*
  - *Screening: Not on any screening lists*

**Chronic Aquatic Toxicity (CA) Score (vH, H, M or L):** DG (no data)

- Authoritative and Screening Lists
  - *Authoritative: Not on any authoritative lists*
  - *Screening: Not on any screening lists*

**Environmental Fate (Fate)**

**Persistence (P) Score (vH, H, M, L, or vL):** *vH*

HeiQ AGS-20 was assigned a score of very high for persistence based on professional judgement.Silicon dioxide and silver are expected to be persistent because they are inorganic, however there was no data on how long AGS-20 would remain in composite form if released into the environment. The confidence level is reported as low due to the use of professional judgement in score determination.

- Authoritative and Screening Lists
  - *Authoritative: Not on any authoritative lists*
  - *Screening: Not on any screening lists*

**Bioaccumulation (B) Score (vH, H, M, L, or vL):** DG (no data)

- Authoritative and Screening Lists
  - *Authoritative: Not on any authoritative lists*
  - *Screening: Not on any screening lists*

**Physical Hazards (Physical)**

**Reactivity (Rx) Score (vH, H, M or L): L**

AGS-20 was assigned a score of Low for Reactivity. The US EPA has waived data requirements for reactivity, explodability, and flammability for AGS-20 due to low concern (i.e. contains no oxidizing or reducing agent, does not contain combustible liquid, and is not potentially explosive) (EPA 2010b). The confidence level is reported as high based on the conclusion was made under US regulatory review.

- Authoritative and Screening Lists
  - *Authoritative: Not on any authoritative lists*
  - *Screening: Not on any screening lists*

**Flammability (F) Score (vH, H, M or L): L**

AGS-20 was assigned a score of Low for Flammability. The US EPA has waived data requirements for reactivity, explodability, and flammability for AGS-20 due to low concern (i.e. contains no oxidizing or reducing agent, does not contain combustible liquid, and is not potentially explosive) (EPA 2010b). The confidence level is reported as high based on the conclusion was made under US regulatory review.

- Authoritative and Screening Lists
  - *Authoritative: Not on any authoritative lists*
  - *Screening: Not on any screening lists*

**References**

Egger, S., R.P. Lehmann, , M.J. Height, , M.J. Loessner, and M. Schuppler, , 2009. Antimicrobial Properties of a Novel Silver-Silica Nanocomposite Material. Appl Environ Microbiol. May:2973-2976.

English, J.C. 2012. ‘Nanosilver’ as used in textiles Phase I: Scoping Project. NSF International. Feb 23, 2012. 9126858-tr01.

EPA, 2010a. HeiQ AGS-20: The Speciation of Silver Nanoparticles in Antimicrobials Fabric, March 5, 2010. EPA-HQ-OPP-2009-1012-0015.pdf.

EPA 2010b. Proposed Decision Document for the Registration of HeiQ AGS-20 as a Materials Preservative in Textiles, August 12, 2010. EPA-HQ-OPP-2009-1012-0020.pdf.

EPA, 2009a. Product Chemistry Review, HeiQ AGS-20, June 30, 2009. EPA-HQ-OPP-2009-1012-0013.pdf.

EPA 2009b. Memorandum, Acute Toxicity Review for EPA Reg. No.: 85249-R, Product Name: HeiQ AGS-20 DP Barcode: D364588, July 2, 2009. EPA-HQ-OPP-2009-1012-0005.pdf.

EPA, 2009c. Literature Review of EPA File Symbol 85249-R, HeiQ AGS 20 DP Barcode: 366658, July 14, 2009. EPA-HQ-OPP-2009-1012-0009.pdf.

# APPENDIX A: Hazard Benchmark Acronyms

**(alphabetical order)**

**(AA) Acute Aquatic Toxicity**

**(AT) Acute Mammalian Toxicity**

**(B) Bioaccumulation**

**(C) Carcinogenicity**

**(CA) Chronic Aquatic Toxicity**

**(Cr) Corrosion/ Irritation (Skin/ Eye)**

**(D) Developmental Toxicity**

**(E) Endocrine Activity**

**(F) Flammability**

**(IrE) Eye Irritation/Corrosivity**

**(IrS) Skin Irritation/Corrosivity**

**(M) Mutagenicity and Genotoxicity**

**(N) Neurotoxicity**

**(P) Persistence**

**(R) Reproductive Toxicity**

**(Rx) Reactivity**

**(SnS) Sensitization- Skin**

**(SnR) Sensitization- Respiratory**

**(ST) Systemic/Organ Toxicity**

**Appendix B**

**Optional Hazard Summary Table**

| **Route** | **GreenScreen™Hazard Ratings: AGS-20 (silver-silica nanocomposite containing silver nanoparticles imbedded in a matrix of amorphous silicon dioxide)** | | | | | | | | | | | | | | | | | | | |
| --- | --- | --- | --- | --- | --- | --- | --- | --- | --- | --- | --- | --- | --- | --- | --- | --- | --- | --- | --- | --- |
|  | **Group I Human** | | | | | **Group II and II Human** | | | | | | | | | **Ecotox** | | **Fate** | | **Physical** | |
|  | **C** | **M** | **R** | **D** | **E** | **AT** | **ST** | | **N** | | **SnS** | **SnR** | **IrS** | **IrE** | **AA** | **CA** | **P** | **B** | **RX** | **F** |
|  |  |  |  |  |  |  | _Single_ | _Repeated_ | _Single_ | _Repeated_ |  |  |  |  |  |  |  |  |  |  |
| o | DG | DG | DG | DG | DG | *L* | DG | DG | DG | DG | *L* | *DG* | *L* | *M* | DG | DG | *vH* | DG | **L** | **L** |
| d | DG |  | DG | DG |  | *L* | DG | DG | DG | DG |  |  |  |  |  |  |  |  |  |  |
| i | DG |  | DG | DG |  | *M* | DG | DG | DG | DG |  |  |  |  |  |  |  |  |  |  |

1. Use GreenScreen® Assessment Procedure (Guidance) V1.2 [↑](#footnote-ref-1)
2. See GreenScreen Guidance V1.2 [↑](#footnote-ref-2)
3. Note any composition or hazard attributes of the chemical product relevant to how it is manufactured. For example, certain synthetic pathways or processes result in typical contaminants, by-products or transformation products. Explain any differences between the manufactured chemical product and the GreenScreen assessment of the generic chemical by CAS #. [↑](#footnote-ref-3)
4. See Appendix A for a glossary of hazard endpoint acronyms [↑](#footnote-ref-4)
5. See Appendix B for alternative GreenScreen Hazard Summary Table (Classification presented by exposure route) [↑](#footnote-ref-5)
6. For inorganic chemicals only, see GreenScreen Guidance V1.2 Section 14.4. (Exceptions for Persistence) [↑](#footnote-ref-6)
7. For Systemic Toxicity and Neurotoxicity, repeated exposure data are preferred. Lack of single exposure data is not a Data Gap when repeated exposure data are available. In that case, lack of single exposure data may be represented as NA instead of DG. See GreenScreen Guidance V1.2 Section 9.3. [↑](#footnote-ref-7)
8. See GreenScreen Guidance V1.2 Section 13 [↑](#footnote-ref-8)
9. A moiety is a discrete chemical entity that is a constituent part or component of a substance. A moiety of concern is often the parent substance itself for organic compounds. For inorganic compounds, the moiety of concern is typically a dissociated component of the substance or a transformation product. [↑](#footnote-ref-9)
10. The CPA “Red List” refers to chemicals 1. flagged as Benchmark 1 using the GreenScreen™ List Translator or 2. flagged as Benchmark 1 or 2 using the GreenScreen™ List Translator and further assessed and assigned as Benchmark 1. The most recent version of the GreenScreen™ List Translator should be used. [↑](#footnote-ref-10)
11. The way you conduct assessments for transformation products depends on the Benchmark Score of the parent chemical (See Guidance). [↑](#footnote-ref-11)
